# Calcium channelopathies and intellectual disability: a systematic review

**DOI:** 10.1186/s13023-021-01850-0

**Published:** 2021-05-13

**Authors:** Miriam Kessi, Baiyu Chen, Jing Peng, Fangling Yan, Lifen Yang, Fei Yin

**Affiliations:** 1 Department of Pediatrics, Xiangya Hospital, Central South University, Changsha, 410008 Hunan China; 2 Hunan Intellectual and Developmental Disabilities Research Center, Changsha, Hunan China; 3 Kilimanjaro Christian Medical University College, Moshi, Tanzania; 4 Mawenzi Regional Referral Hospital, Moshi, Tanzania

**Keywords:** Intellectual disability, Global developmental delay, Epilepsy, Calcium channelopathies, Genes, Variants, Cerebellar atrophy, Review

## Abstract

**Background:**

Calcium ions are involved in several human cellular processes including corticogenesis, transcription, and synaptogenesis. Nevertheless, the relationship between calcium channelopathies (CCs) and intellectual disability (ID)/global developmental delay (GDD) has been poorly investigated. We hypothesised that CCs play a major role in the development of ID/GDD and that both gain- and loss-of-function variants of calcium channel genes can induce ID/GDD. As a result, we performed a systematic review to investigate the contribution of CCs, potential mechanisms underlying their involvement in ID/GDD, advancements in cell and animal models, treatments, brain anomalies in patients with CCs, and the existing gaps in the knowledge. We performed a systematic search in PubMed, Embase, ClinVar, OMIM, ClinGen, Gene Reviews, DECIPHER and LOVD databases to search for articles/records published before March 2021. The following search strategies were employed: ID and calcium channel, mental retardation and calcium channel, GDD and calcium channel, developmental delay and calcium channel.

**Main body:**

A total of 59 reports describing 159 cases were found in PubMed, Embase, ClinVar, and LOVD databases. Variations in ten calcium channel genes including *CACNA1A, CACNA1C*, *CACNA1I, CACNA1H, CACNA1D*, *CACNA2D1*, *CACNA2D2*, *CACNA1E*, *CACNA1F*, and *CACNA1G* were found to be associated with ID/GDD. Most variants exhibited gain-of-function effect. Severe to profound ID/GDD was observed more for the cases with gain-of-function variants as compared to those with loss-of-function. *CACNA1E*, *CACNA1G*, *CACNA1F*, *CACNA2D2* and *CACNA1A* associated with more severe phenotype. Furthermore, 157 copy number variations (CNVs) spanning calcium genes were identified in DECIPHER database. The leading genes included *CACNA1C*, *CACNA1A*, and *CACNA1E*. Overall, the underlying mechanisms included gain- and/ or loss-of-function, alteration in kinetics (activation, inactivation) and dominant-negative effects of truncated forms of alpha1 subunits. Forty of the identified cases featured cerebellar atrophy. We identified only a few cell and animal studies that focused on the mechanisms of ID/GDD in relation to CCs. There is a scarcity of studies on treatment options for ID/GDD both in vivo and in vitro.

**Conclusion:**

Our results suggest that CCs play a major role in ID/GDD. While both gain- and loss-of-function variants are associated with ID/GDD, the mechanisms underlying their involvement need further scrutiny.

**Supplementary Information:**

The online version contains supplementary material available at 10.1186/s13023-021-01850-0.

## Introduction

Intellectual disability (ID) is characterized by limitations in both intellectual functioning and adaptive behaviour, which manifests before the age of 18 [1]. Global developmental delay (GDD) is defined as a profound delay of ≥ 2 standard deviations below the mean in ≥ 2 developmental domains [2]. GDD and ID represent clinically defined and recognized symptoms that are related but not necessarily synonymous. GDD is used for the children aged below 5 years while ID is for those aged 5 years and above [3]. Two-third of the children meriting the diagnosis of GDD in the preschool years, when reassessed later at school age, they continue to meet the diagnostic criteria for ID [4, 5]. Moreover, many older children diagnosed with ID currently, were initially diagnosed with GDD. Therefore, these entities have common features and both represent defects or disorders in learning [6]. Hence, they have a common approach in terms of evaluation and understanding of their etiology. The prevalence of ID/GDD world-wide is 10.37/1000 population according to meta-analysis [7]. It can occur in isolation or in combination with other neurological conditions such as epilepsy, autism spectrum disorders, attention deficit hyperactivity disorder, sensory impairment or congenital malformations. This condition incurs huge cost for the provision of adequate services in the society, and it is associated with stigma, mental and physical complications [7]. Notably, 30% of the cases with ID/GDD have comorbid mental health problems [5]. Neuropsychological tests are used to diagnose ID/GDD, however, the diagnosis is often initially formulated based on clinical judgment rather than on formal standardized assessments especially for the young patients [6] because those tests are limited by the age.

Approximately 65% of the cases with moderate-to-severe ID/GDD have genetic etiologies such as chromosome structural abnormalities, chromosome aneusomies, genomic disorders, and monogenic diseases [8–11]. With the introduction of next generation sequencing technologies, new ID/GDD genes are now being identified rapidly of which unpin the pathophysiology and provide new targets for treatment. Up to the present time, 450 genes have been implicated in ID/GDD; 400 genes for syndromic ID/GDD and 50 genes for non-syndromic ID [12]. Some of these genes participate in calcium signaling pathway. Prenatally, calcium-facilitated depolarization regulates neural proliferation, migration, and differentiation during the formation of the cerebral cortex [13]. Postnatally, calcium ions modulate cellular excitability by modelling synapses and sensory neural circuits [13]. Calcium ions also contribute to the membrane potential and function as an important signalling molecule [14]. Several processes in humans, including mitochondrial functions [15, 16], transcription, release of neurotransmitters, neurite outgrowth, and activation of some enzymes [14] depend on calcium ions. Noteworthy, mitochondria play a major role in regulating calcium-signalling processes [15, 16]. Besides, ATP is important for regulation of membrane excitability, synaptic transmission, transcription, and apoptosis [15].

Voltage-gated calcium channels belong to the family of 4-domain ion channels. Ten genes encode voltage-gated calcium channels. Each is categorized into one of two major groups: high voltage activated (HVA; R-, P/Q-, N- and L-types), and low voltage activated (LVA; T-type) [14, 17]. The HVA calcium channels are heteromultimeric protein complexes consisting of the pore-forming Cavα1, Cavδ, Cavβ and Cavα2δ subunits [14, 17]. By contrast, low voltage-activated calcium channels are comprised of only the Cavα1. Cav1, Cav2, and Cav3 are subfamilies [14, 17]. All Cavα1 subunits have four major transmembrane domains, and each consist of six membrane-spanning helices (termed S1–S6) [14]. Calcium ions enter into the cell when the channel is open and the opposite happens when it is closed. The movement of calcium ions in and out of the cell is regulated by calcium- and voltage-dependent inactivation of calcium ion channels [14]. S4 segment is positively charged, thus responsible for controlling voltage-dependent activation. The loop between S5 and S6 consists of negatively charged residues (glutamate or aspartate) that form the selectivity filter [14]. Large cytoplasmic linker connects the chief membrane domains regions and are within cytoplasmic N and C termini. Noteworthy, these cytoplasmic domain regions are important for modulating channel function via second messenger and protein–protein interaction [14, 17]. The most common type of mutations in this voltage-gated calcium channels is missense followed by deletion (according to this review). Consequently, mutations in S4 domain between S5 and S6, and in cytoplasmic linker that connects the chief membrane domains can alter the ability of the channels to regulate calcium influx/efflux. Overall, mutations in calcium channels can; (1) decrease channel function (loss-of function) or expression, (2) increase channel function (gain-of function) or expression, and (3) produce a disease without altering the electrophysiological properties of the channels [14]. Table 1 summarizes the general information related to the channels including the name of the gene, type of the current produced, neuronal localization, distribution, and pharmacology.

**Table 1:** An overview of calcium channel subunits

Gene	Name	Subunit	Current type	Neuronal localization	Distribution	Role	Function	Pharmacology	References
*CACNA1A*	Calcium voltage-gated channel subunit alpha1 A	Cav2.1	P/Q-type	Pre-synaptic region	Cerebral cortex, thalamus, hypothalamus, hippocampus, and cerebellum	Form the conducting pore	Involved in muscle contraction, hormone or neurotransmitter release, and gene expression	Can be blocked by omega-agatoxin-IVA	[215, 216]
*CACNA1B*	Calcium voltage-gated channel subunit alpha1 B	Cav2.2	N-type	Pre-synaptic region	Midbrain, cerebellar cells, spinal cord motor neurons and cholecystokinin‐expressing interneurons	Form the conducting pore	Involved in muscle contraction, hormone or neurotransmitter release, gene expression, cell motility, cell division and cell death as well as neuronal firing	Can be blocked by omega-conotoxin-GVIA and omega-agatoxin-IIIAS	[217, 218]
*CACNA1C*	Calcium voltage-gated channel subunit alpha1 C	Cav1.2	L-type	Neuronal synapses and dendrites	Brain and cardiac muscles	Form the conducting pore	Maintain synaptic plasticity, neuronal survival and fear conditioning	Can be blocked by dihydropyridine and lead	[217, 219, 220]
*CACNA1D*	Calcium voltage-gated channel subunit alpha1 D	Cav1.3	L-type	Post-synaptic	Brain (dendritic spines), inner hair cell and organ of Corti and heart	Mediate the entry of calcium ions into excitable cells	Regulates contraction, secretion, and neurotransmission and gene expression	Can be blocked by dihydropyridine	[217, 221]
*CACNA1E*	Calcium voltage-gated channel subunit alpha1 E	Cav2.3	R-type	Both pre-synaptic and post-synaptic	Hippocampus, kidney, retina, spleen and pancreatic islet cells	Mediate the entry of calcium ions into excitable cells	Involved in neurotransmitter release and long-term potentiation	Can be blocked by SNX-482	[222]
*CACNA1F*	Calcium voltage-gated channel subunit alpha1 F	Cav1.4	L-type	Pre-synaptic region	Hippocampus, cerebellum and retina	Mediate the entry of calcium ions into excitable cells	Involved in neurotransmitter release	Can be blocked by dihydropyridine	[217]
*CACNA1G*	Calcium voltage-gated channel subunit alpha1 G	Cav3.1	T-type	Post-synaptic region	Cerebellum, hippocampus, thalamus and heart	Mediate the entry of calcium ions into excitable cells	Muscle contraction, hormone or neurotransmitter release, gene expression, cell motility, cell division, and cell death	Can be blocked by mibefradil Hybrid of dearomatized isoprenylated acylphloroglucinol (DIAP) and monoterpenoid, hypatone A (agonist and antagonist)	[186, 223]
*CACNA1H*	Calcium voltage-gated channel subunit alpha1 H	Cav3.2	T-type	Plasma membrane	Brain cortex, amygdala, caudate nucleus, putamen, kidney, liver, and heart	Forms the pore	Regulates contraction, secretion, neurotransmission and gene expression	Can be blocked by efonidipine, felodipine, isradipine, and nitrendipine	[224]
*CACNA1I*	Calcium voltage-gated channel subunit alpha 1I	Cav3.3	T-type	Plasma membrane	Cerebellum, thalamus, cerebral cortex, adrenal gland and thyroid gland	Forms the pore	Regulates muscle contraction, hormone or neurotransmitter release, gene expression, cell motility, cell division and cell death	Can be blocked by nickel and mibefradil	[223]
*CACNA1S*	Calcium Voltage-Gated Channel subunit alpha 1S	Cav1.1	L-type	Plasma membrane	Muscles, brain cortex	Forms the pore	Regulates muscle contraction	Can be blocked by dihydropyridine	[217]

Calcium channelopathies are associated with several neurological disorders including ID/GDD, epilepsy, migraine, and ataxia [14]. Our hypothesis was that loss-of-function mutations are expected to induce ID/GDD, gain-of-function mutations are expected to enhance learning and memory. Similarly, gain-of-function and not loss-of-function mutations are expected to cause epilepsy. Surprisingly, studies showed that both gain- and loss-of-function mutations in genes encoding calcium channels could lead to epilepsy [18–23]. These mutations can dysregulate intrinsic gating processes and cell signalling pathways, which are involved in regulating channel activity and calcium trafficking through the plasma membrane [24]. As a result, these mutations can impair mitochondrial function, neurotransmitter release, and synaptic plasticity.

Our recent review revealed that potassium channelopathies contribute largely to the development of ID/GDD, and both gain- and loss-of-function variants were involved [25]. We observed that potassium channelopathies play an important role in the development of ID/GDD [25]. However, the role of calcium channelopathies in ID/GDD is unknown. Besides, it is unclear whether both gain- and loss-of-function mutations in calcium channel genes can lead to ID/GDD, and what are the possible underlying mechanisms.

Approximately 30% (range, 2–80%) of the cases with ID/GDD have nonspecific brain abnormalities according to magnetic resonance imaging (MRI), and computed tomography [26]. Nevertheless, the contribution of neuroimaging studies in understanding the underlying etiology of this condition range from 0.2 to 2.2% only [27]. Noteworthy, for some genes, brain changes are age-dependent, therefore, normal conventional MRI can be found at an early age [28], however, with an increase of age, malformations can be noticed [29, 30]. Cases with normal conventional MRI can have concealed malformations, which can be detected by advanced brain imaging methods. For instance, functional MRI (fMRI) can detect brain abnormalities that conventional MRI cannot spot in temporal lobe epilepsy and autism [31, 32]. Similarly, proton magnetic resonance spectroscopy (H-MRS) can detect abnormalities in cases diagnosed with neuronopathic Gaucher’s disease (NGD) [33]. Thanks to the advanced technology, the fMRI and H-MRS can also predict the severity of the disease even at an early age of which might be correlated with genotypes. Razek AA et al. revealed in their study that in children with NGD, there is a correlation between choline/creatine ratio and modified disease severity scoring system and genotypes [33]. Although abnormal standardized neuropsychological tests are enough to establish the diagnosis of ID/GDD, those tests can be affected by patient age. Fortunately, some neuroimaging studies can overcome the age limitation; alteration of metabolites revealed by H-MRS and diffusion-weighted magnetic resonance imaging (DWI) can foretell the severity of the cognitive dysfunction. In one study, neuropsychological test results correlated with apparent diffusion coefficient value and metabolic change for the children diagnosed with minimal hepatic encephalopathy with liver cirrhosis signifying that altered metabolic changes and cerebral edema were responsible for cognitive changes [34]. Whether there is a link between mutations in calcium channel genes, brain malformations, metabolic changes, and ID/GDD is yet to be found. Early detection of metabolic and other brain changes can aid in prevention of further cognitive decline.

Based on the important roles of calcium ions in the development of the neural cortex and signalling processes, we hypothesised that calcium channelopathies might contribute to the development of ID/GDD. To prove this, we listed all calcium channel gene variants previously reported in association with ID/GDD. Each mutation was considered in context of the associated degree of severity, current knowledge about possible mechanisms (gain- or loss-of-function), relevant advancements in animal models, treatments, and existing gaps in knowledge. We further aimed to investigate morphological brain anomalies associated with ID/GDD in patients with calcium channelopathies. We also discuss the possible relationship between calcium channelopathies, mitochondria dysfunction, epileptic discharges, cerebellar morphological changes, and ID/GDD. This review will help future studies on the mechanisms of ID/GDD to develop novel treatment strategies for this condition. Although previous narrative reviews summarised the relationship between calcium channelopathies and epilepsy as well as autism spectrum disorder [17, 35–37] to the best of our knowledge, this is the first systematic review to explore the relationship between calcium channelopathies and ID/GDD.

## Methods

### Literature search and selection

The review was conducted according to the Preferred Reporting Items for Systematic Reviews and Meta-Analyses statement [38]. An extensive literature search was conducted in PubMed, Embase, ClinVar, LOVD, OMIM, ClinGen, Gene Reviews and DECIPHER databases to find any relevant study/record published for all years until March 2021. The following search strategies were employed: ID and calcium channel, mental retardation and calcium channel, global developmental delay (GDD) and calcium channel, developmental delay (DD) and calcium channel (Additional file 1). The search strategies were created in consultation with a librarian and were used by three independent reviewers to select papers that met our review objectives.

This review included several kinds of clinical and epidemiological studies such as cohorts, case-controls, cross-sectionals, case series, and case reports. We selected studies that included cases with ID/GDD and calcium-channel gene variants. We excluded papers involving cases of ID/GDD with other types of channelopathies (sodium, potassium, and chloride) or other gene variants. Moreover, we did not include studies that documented patients with calcium channelopathies but no information related to ID/GDD. Lastly, we excluded all non-English papers, abstracts, reviews, patents, book chapters, and conference papers. The reference lists of retrieved studies were hand-searched to identify additional relevant reports.

### Data extraction

Two independent reviewers screened the titles and abstracts of candidate papers and subsequently read the entire content of those that apparently met our inclusion criteria. The accuracy of the retrieved information was determined through discussion and consensus among the authors. We collected from articles that met inclusion criteria information related to calcium channel gene variants, phenotype associated with ID/GDD, degree of ID (mild, moderate, severe, and profound), electrophysiological results (gain- or loss-of-function), brain MRI results if reported, and the corresponding references. All identified candidate genes were further researched using the OMIM, ClinVar, Embase, LOVD, and PubMed databases to determine their function, expression profile, any related information gleaned from animal and functional cell studies, available treatments, and how they could contribute to possible mechanisms underlying ID/GDD.

## Results

**Fig. 1:**
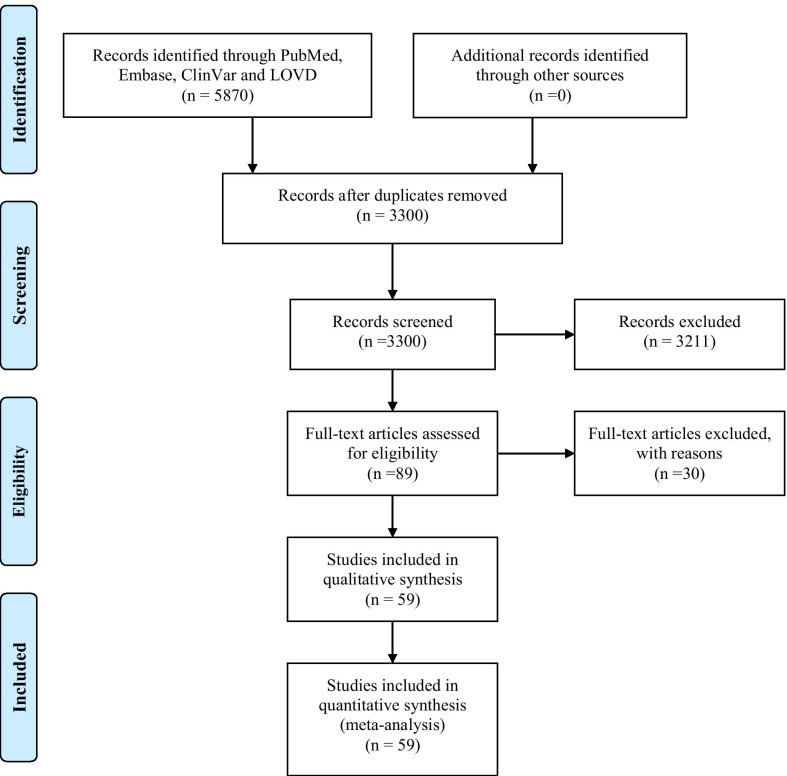
A summary of the steps used for the literature selection

Five thousand eight hundred and seventy articles were retrieved from the initial search. Fifty-nine full-text articles met our inclusion criteria after we excluded non-English papers, abstracts, reviews, patents, book chapters, conference papers, and irrelevant papers on other channelopathies (Fig. 1). Thus, we identified 159 cases documented in 59 papers. Epilepsy was reported in 51.6% (82/159) of the cases. Ten calcium channelopathies related to ID/GDD were identified involving the following genes: *CACNA1A* [18, 19, 39–66], *CACNA1C* [67–73], *CACNA1I* [74], *CACNA1H* [75, 76]*, CACNA2D2* [77–80], *CACNA2D1* [20, 81], *CACNA1D* [21, 22, 82–84], *CACNA1E* [85], *CACNA1F* [86], and *CACNA1G* [23, 87]. The underlying mechanisms included gain- and/ or loss-of-function, alteration in kinetics (activation, inactivation) and dominant-negative effects of truncated forms of alpha1 subunits.

**Fig. 2:**
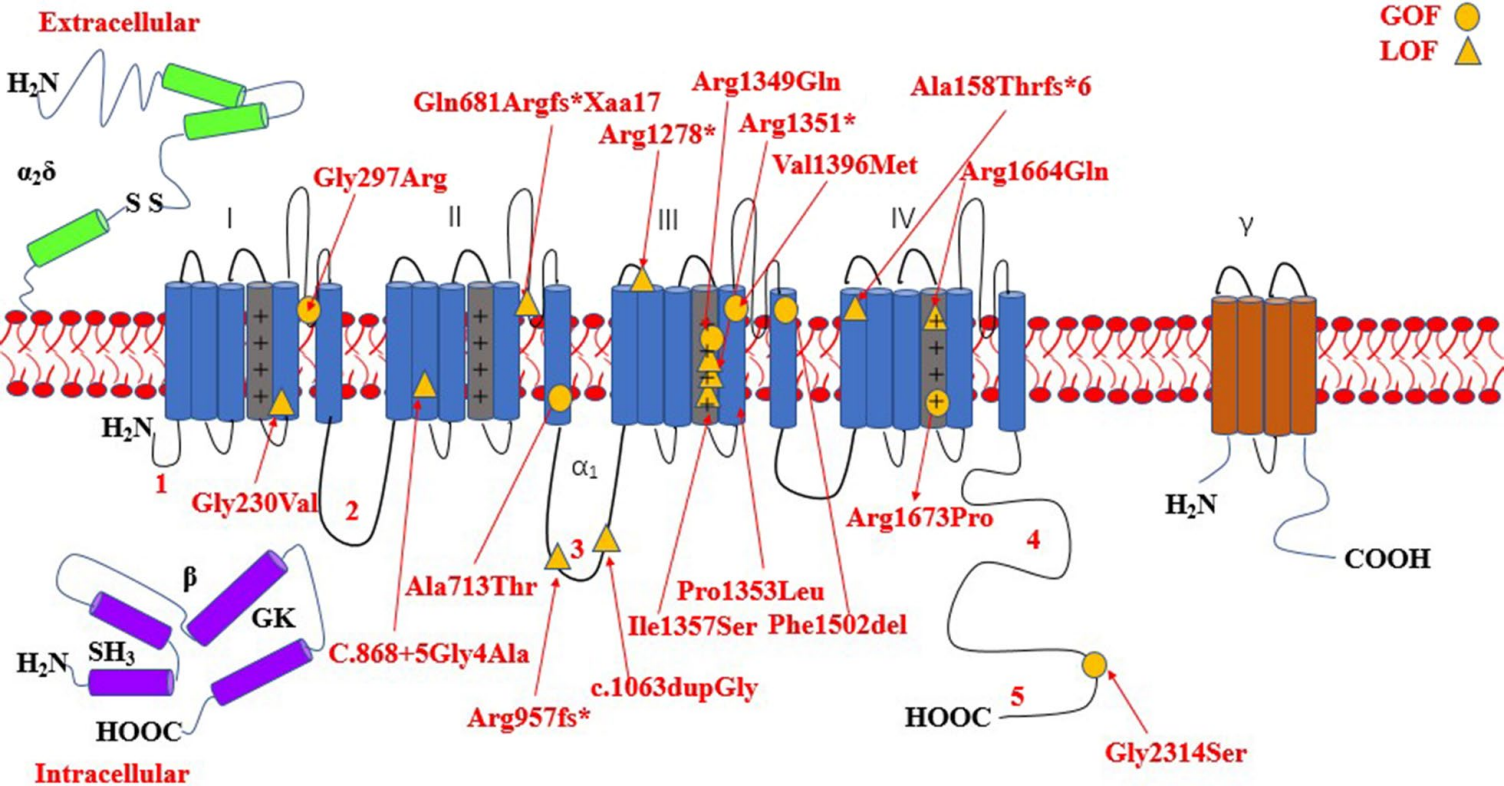
Effects and locations of genetic aberrations for *CACNA1A.* There is a cluster of four critical residues in S4 transmembrane segment of domain III. Round yellow dots represent gain-of- function variants. Triangular yellow dots represent loss-of-function variants

**Fig. 3:**
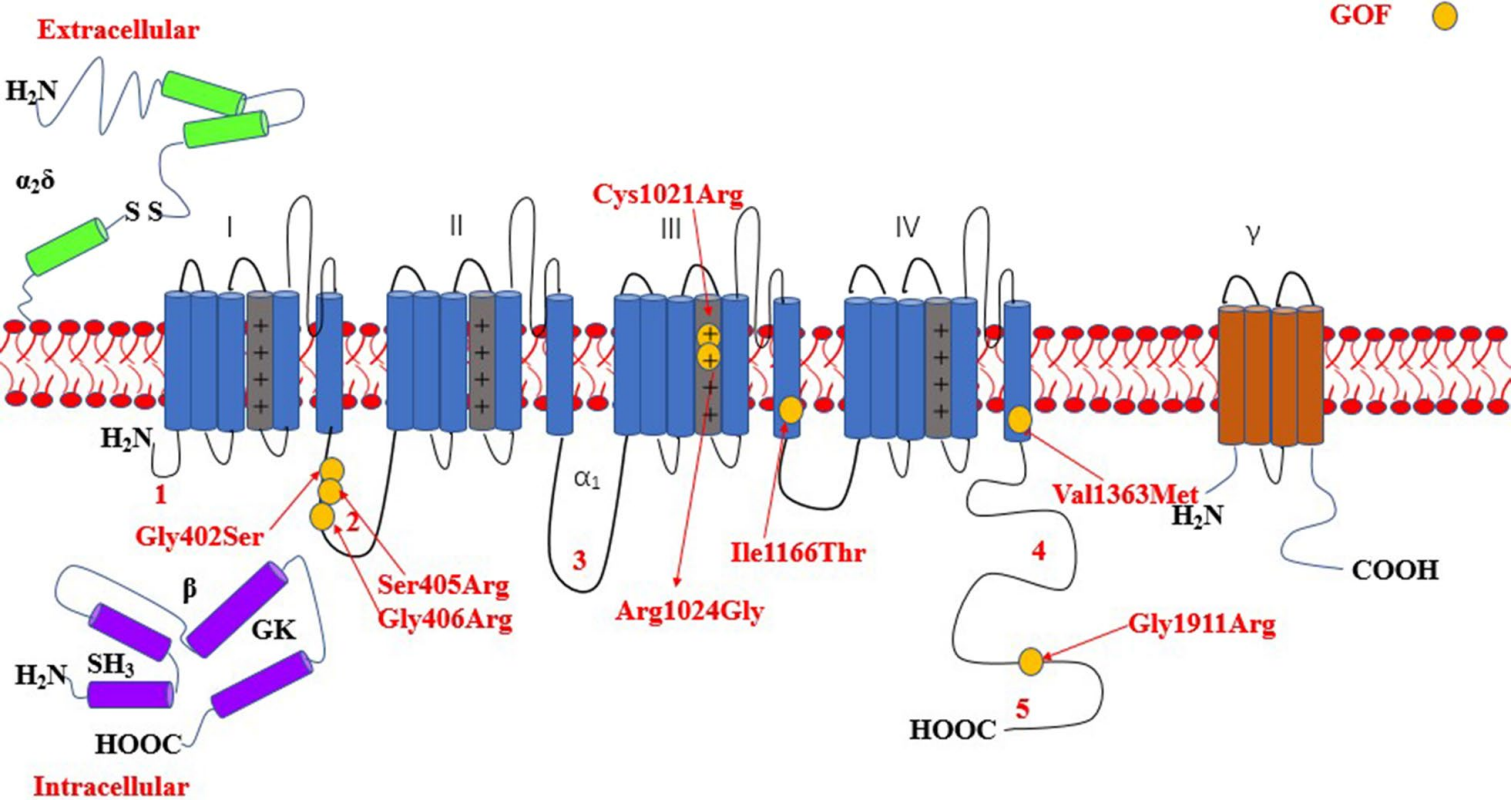
Location of the identified *CACNA1C* amino acid substitutions. There is a cluster of four critical residues in the DI/D II intracellular interlinker. Round yellow dots represent gain-of- function variants

**Fig. 4:**
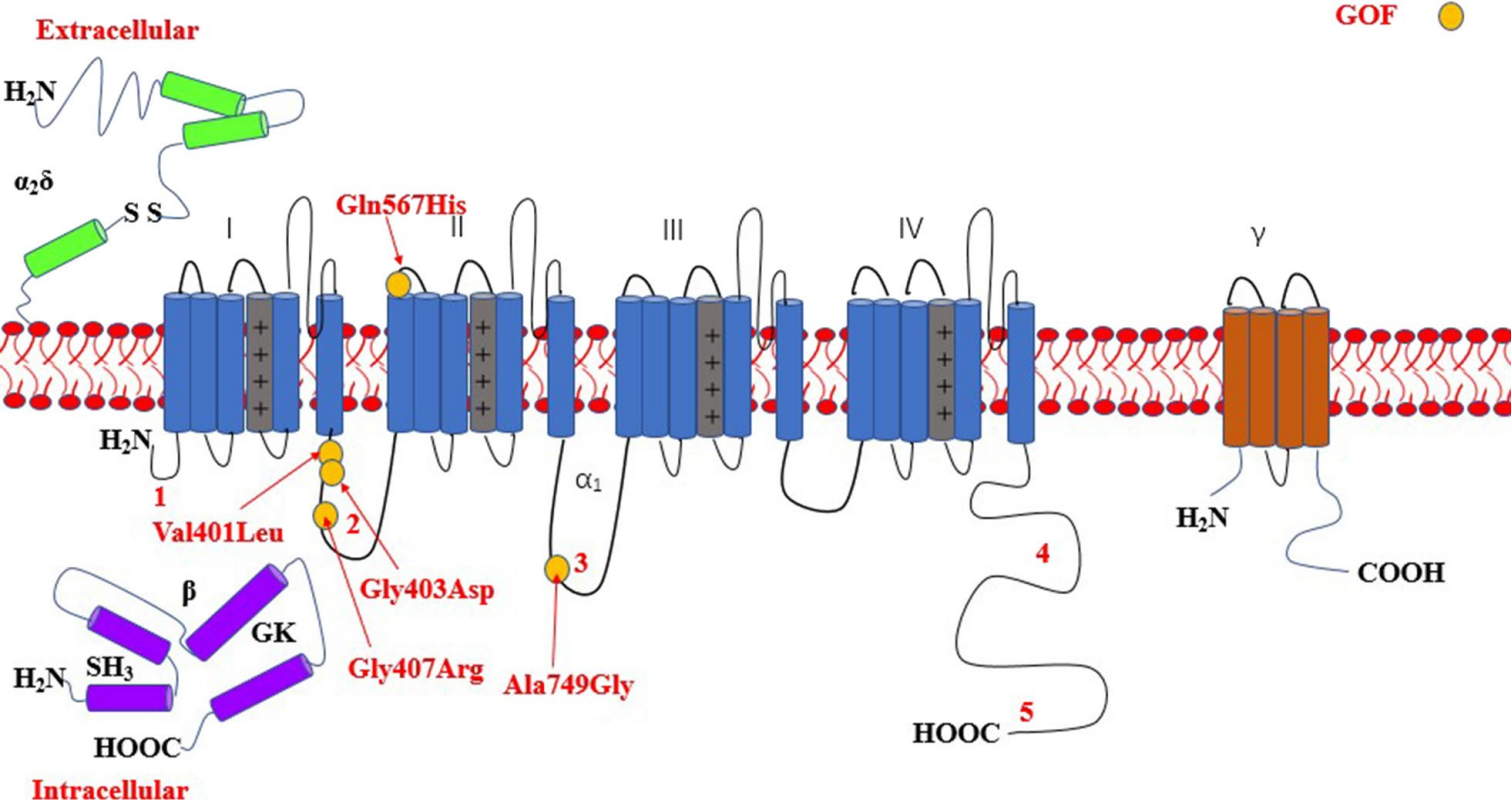
Location of the identified *CACNA1D* amino acid substitutions. There is a cluster of three critical residues in the domain I/domain II intracellular interlinker. Round yellow dots represent gain-of- function variants

**Fig. 5:**
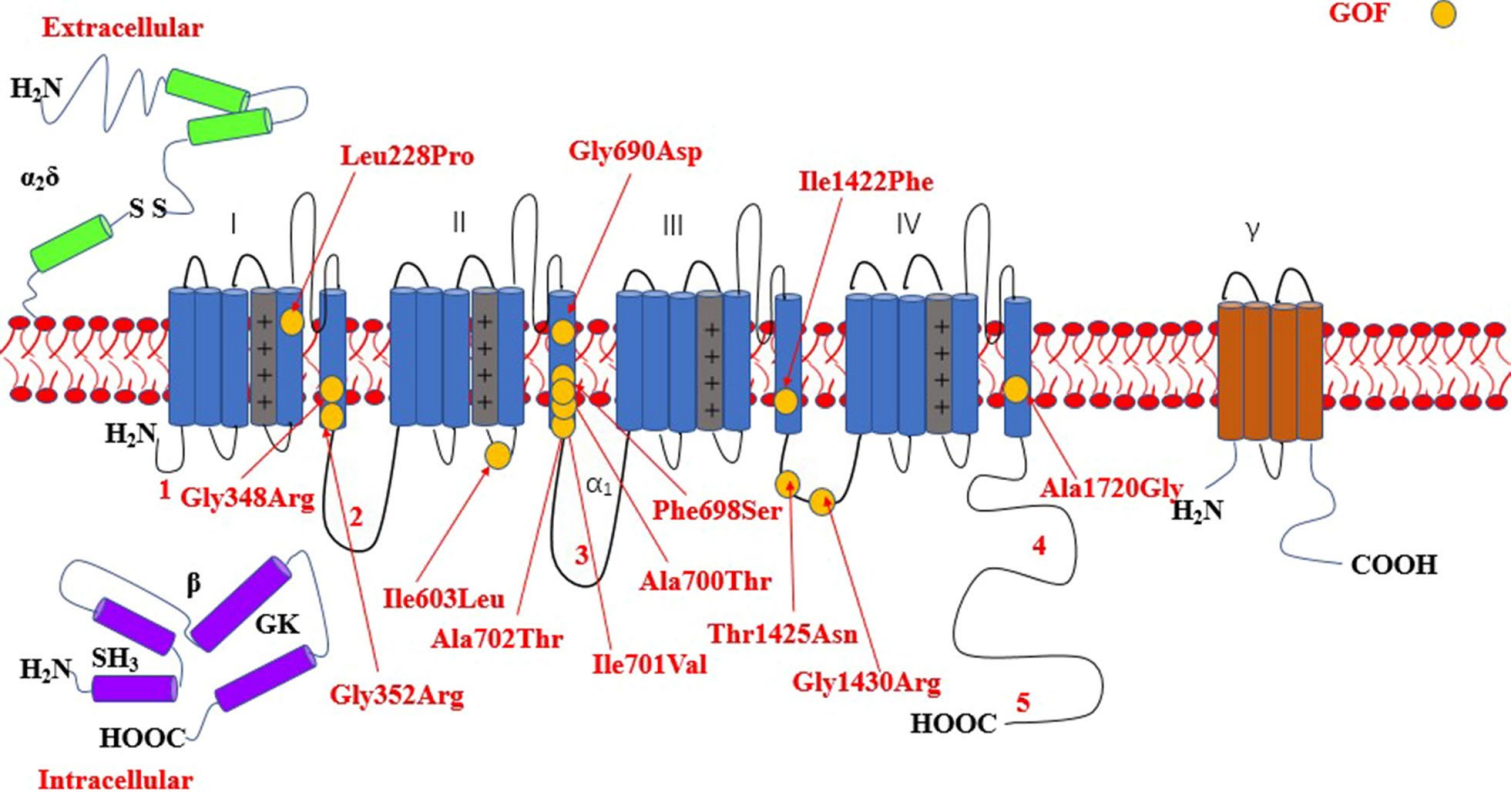
Location of the identified *CACNA1E* amino acid substitutions. There is a cluster of five critical residues important for gating in S6 transmembrane segment of domain II. Round yellow dots represent gain-of- function variants

**Fig. 6:**
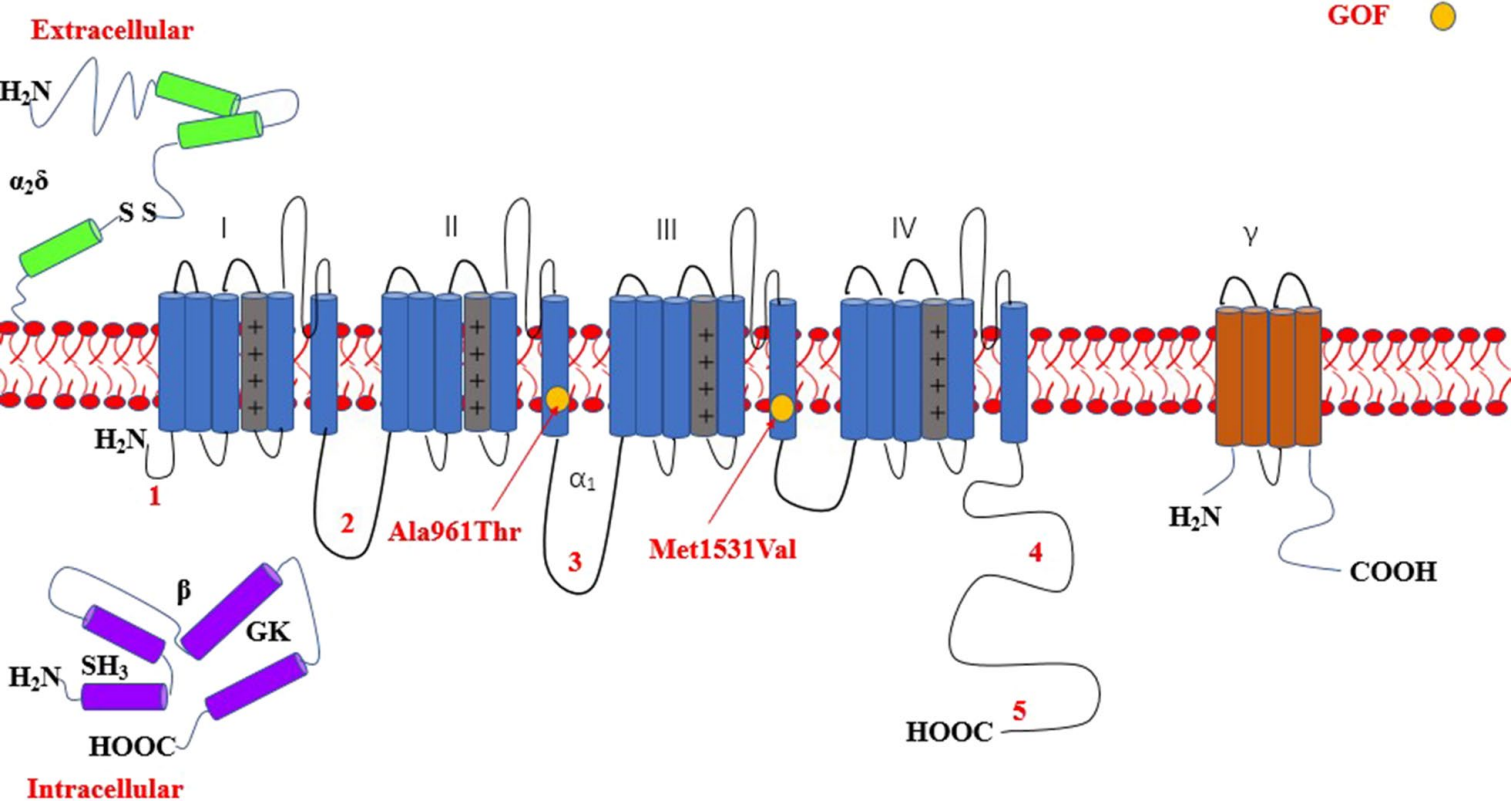
Location of the identified *CACNA1G* amino acid substitutions. Round yellow dots represent gain-of- function variants

**Fig. 7:**
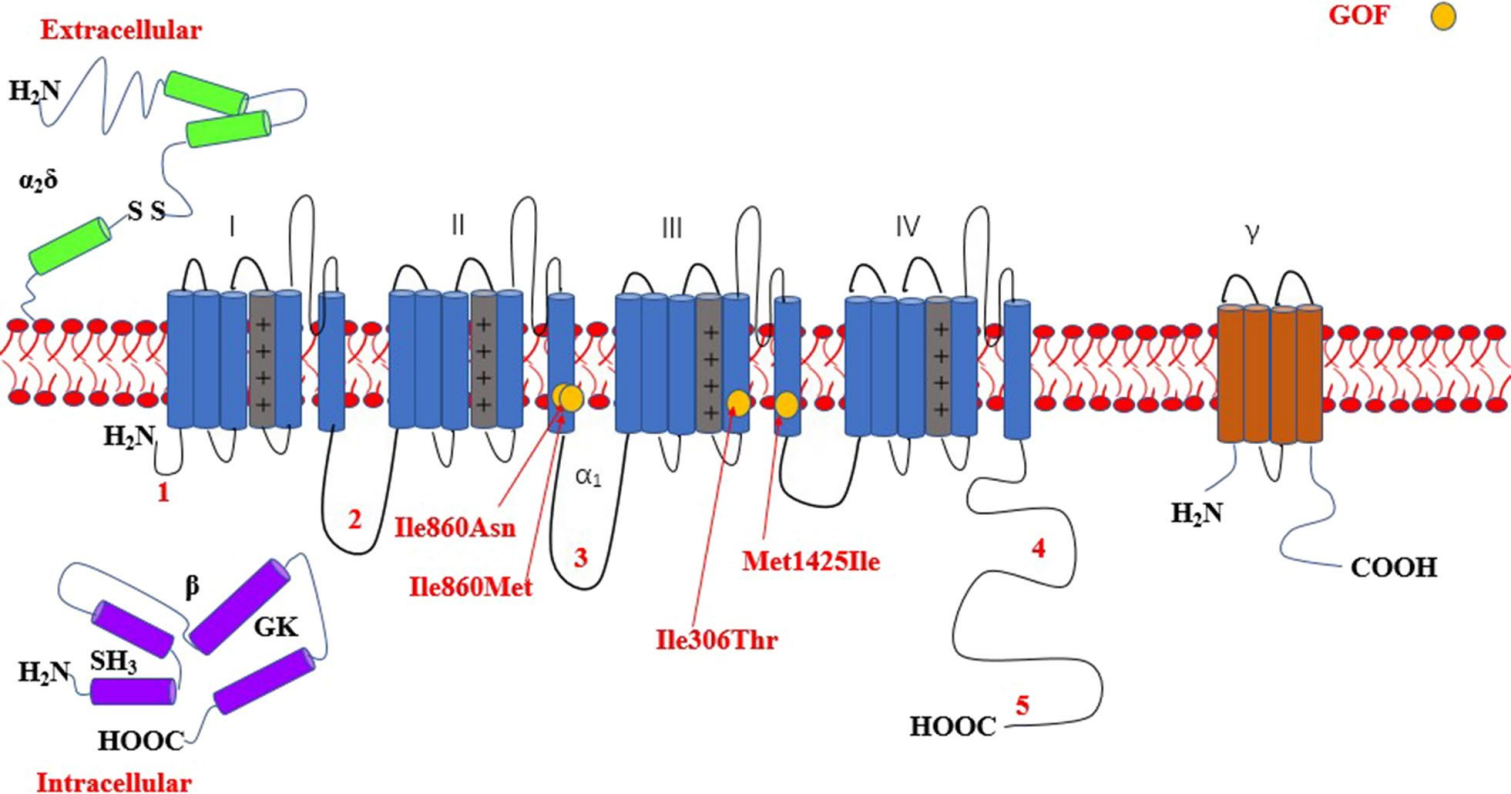
Location of the identified *CACNA1H* amino acid substitutions. Round yellow dots represent gain-of- function variants

**Fig. 8:**
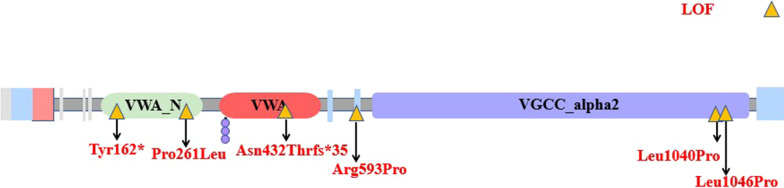
Location of the identified *CACNA2D2* amino acid substitutions. Triangular yellow dots represent loss-of-function variants

The most common affected calcium genes were *CACNA1A, CACNA1E, CACNA1C* and *CACNA1D.* Most variants exhibited gain-of-function effect. Severe to profound ID/GDD was observed more for the cases with gain-of-function variants as compared to those with loss-of-function variants. *CACNA1E*, *CACNA1G*, *CACNA1F*, *CACNA2D2* and *CACNA1A* associated with more severe phenotype (Additional file 2: Table S1). Figures 2, 3, 4, 5, 6, 7 and 8 summarize the effects of genetic aberrations. The S4 transmembrane segment of domain III was the hotspot for *CACNA1A*-related ID/GDD (Fig. 2), domain I/domain II intracellular interlinker for *CACNA1C* (Fig. 3), S6 transmembrane segment of domain II for *CACNA1E* (Fig. 5), and the domain I/domain II intracellular interlinker for *CACNA1D* (Fig. 4). The detailed genotype–phenotype list can be found in Additional file 3.

Moreover, 157 copy number variations (CNVs) spanning calcium genes were identified in DECIPHER database but it was difficult to include them here due to consent issues. The leading CNVs were those encompassing *CACNA1C*, *CACNA1A*, *CACNA1E*, *CACNA1F* and *CACNA1G*. More details can be found in DECIPHER database (decipher@sanger.ac.uk).

Cerebellar atrophy was reported in 25% (40) of the identified cases, cortical atrophy in 8.8% (14), optic atrophy in 1.3% (2), white matter changes in 5% (8), and other anomalies of the central nervous system in 5% (8). Normal brain magnetic resonance imaging findings accounted for 24.5% (39) of the identified cases and 34.6% (55) of the cases had no information related to brain imaging results. Two cases with gain-of-function variants who underwent muscle biopsy showed mitochondrial dysfunction: decreased mitochondrial complex I and III activity for the case with *CACNA1C* variant and partial deficits in complexes II and III for the case carrying *CACNA1A* variant (Additional file 2: Table S1). There is scarcity of cell and animal models for ID/GDD. Several modulators and pathways have been proposed for other calcium channel-related conditions. The commonest involved pathway is apoptotic followed by autophagic (Table 2, 3).

**Table 2:** Genes reported to associate with ID and availability of animal models, modulators and pathways

Gene	Name	Subunit	Current type	Animal model for ID	Other available animal models	Modulators	Disease pathway
*CACNA1A*	Calcium voltage-gated channel subunit alpha1 A	Cav2.1	P/Q-type	Conditional Ca(V)2.1 knock-out model [225]	Animal models of ataxia [144, 165, 226, 227]. Mouse models of migraine [223, 228–230]. Drosophila model of Spinocerebellar Ataxia Type 6 [231]. Genetic models of epilepsy in mice [232–234].Tottering-6j mice for absence seizures and motor dysfunctions [235, 236]. Cacna1a-mutant GRY rat for absence seizures [143]. Mice model of peripheral nerve injury [237]. The Rolling mouse Nagoya (Cacna1a (tg-rol)) for autosomal dominant cerebellar ataxia (SCA6), familial hemiplegic migraine and episodic ataxia type-2 [238]	Seizures were inhibited by ethosuximide and valproic acid, but not by phenytoin in Tottering-6j mice [235]. Seizures were inhibited by ethosuximide and valproic acid but not phenytoin in Cacna1a-mutant GRY rat for absence seizures [143]. Acetazolamide could abolish stress-induced ataxia in mice [144]	Apoptotic pathway in ataxia [152]
*CACNA1C*	Calcium voltage-gated channel subunit alpha1 C	Cav1.2	L-type	Genetic Cacna1c rat model [239]. Cacna1c knockout model [240]. Mouse line with an inactivation of the Cav1.2 gene in the hippocampus and neocortex (Cav1.2 (HCKO)) [241]. Conditional knockout mice [242]	Mouse model of autism spectrum disorder [243, 244]. Mouse model of Timothy syndrome [245]. Neuropsychiatric disorders animal model [246, 247]. Mouse model of Huntington's disease [248]. Cacna1c HET mice for depression-related behaviors [249]. PLZF knock-out mice for type 2 diabetes mellitus [250]. Cardiac hypertrophy model [148]	Diltiazem could inhibit influenza A virus infection in vivo and in vitro [147]. MiR-135b could inhibit cardiomyocyte hypertrophy [148]. Nifedipine can block Cav1.2 current [146]	Oxidative stress pathway for affective disorders [156] Apoptotic pathway in Timothy syndrome [153] Apoptotic pathway in dilated cardiomyopathy [154]
*CACNA1D*	Calcium voltage-gated channel subunit alpha1 D	Cav1.3	L-type	None	Zebrafish larvae for schizophrenia [251]. Animal model of epilepsy [252]	None	Oxidative stress pathway in hearing loss [157]
*CACNA1E*	Calcium voltage-gated channel subunit alpha1 E	Cav2.3	R-type	Mouse model of Fragile X Syndrome [253]	Sprague–Dawley rats for epilepsy [254]. Cancer pain mouse model [255]. Mouse model of chronic neuropathic pain [256]	None	None
*CACNA1F*	Calcium voltage-gated channel subunit alpha1 F	Cav1.4	L-type	None	Cacna1f (nob2) mice for vision [257, 258]. Cacna1f loss of function model of congenital stationary night blindness [259, 260]. IT mouse line that harbors the gain-of-function mutation for the congenital stationary night blindness type 2 [261]	None	None
*CACNA1G*	Calcium voltage-gated channel subunit alpha1 G	Cav3.1	T-type	None	Mouse model for drowsiness [262]. Mouse model for spinocerebellar ataxia 42 [263]. Tremor model [264, 265]. Mouse model of epilepsy [266–268]. Mouse model of autoimmune encephalomyelitis [269]. Cardiac arrhythmias [270]	None	None
*CACNA1I*	Calcium voltage-gated channel subunit alpha1 I	Cav 3.3	T-type	None	None	Niflumic acid can block Cav3.3 current [149]	None
*CACNA1H*	Calcium voltage-gated channel subunit alpha1 H	Cav3.2	T-type	None	Polygenic rat model of absence epilepsy [271]. Mouse model of visceral hypersensitivity and in irritable bowel syndrome [272]. Mouse model of inflammatory and neuropathic pain [273]	(2S)-6-prenylnaringenin can block Cav3.2 current [150]	Apoptotic pathway in myocardial cells [155] Autophagy pathway [158]
*CACNA2D1*	Calcium voltage-gated channel auxiliary subunit alpha2delta 1	Cav1.3	L-type	None	Conventional knockout mouse using a construct targeting exon 2 of alpha (2)/delta-1 [274]. α2 δ1 Tg model for neuropathic pain [275, 276]	None	None
*CACNA2D2*	Calcium voltage-gated channel auxiliary subunit alpha2delta 2	Cav1.3	L-type	None	The “ducky’ du (2 J) mouse model of ataxia and absence epilepsy [277]. Mouse model of prostate cancer [151]	Gabapentin is a ligand of Cav1.3 [151]	Apoptotic pathway [278]

**Table 3:** Gene reported to associate with ID and availability of functional cell models, modulators and pathways

Gene	Name	Subunit	Current type	Functional model (neuronal stem cells or other cell lines) for ID	Other available functional models	Modulators	Disease pathway
*CACNA1A*	Calcium voltage-gated channel subunit alpha1 A	Cav2.1	P/Q-type	Primary hippocampal rat cultures, HEK293T cells and TsA201 for synaptic plasticity [109]	HEK293 cells for developmental epileptic encephalopathies [19]. Purkinje cells for spinocerebellar ataxia type 6 [279, 280]. Induced pluripotent stem cells (ZZUi0017-A) for spinocerebellar ataxia type 6 [281]. COS-7 cells for spinocerebellar ataxia type 2 [164]. Pheochromocytoma (pc12) cells for cerebellar disorders [282]. HEK293T cells for epilepsy [283]. SH-SY5Y for familial hemiplegic migraine type 1 and episodic ataxia [159]. Purkinje cells for absence epilepsy, dyskinesia, and ataxia phenotypes [284]. Cell-specific mouse mutant lines that suffer from impaired purkinje cell output (Pcd), purkinje cell potentiation (L7-Pp2b), molecular layer interneuron output (L7-Δγ2), and granule cell output (α6-Cacna1a) for cerebellar control of gait and interlimb coordination [285]. HEK293 cells for stroke-like episodes and ataxia [286]. HEK 293 cells for migraine with aura [287, 288]. Induced pluripotent stem cell line (ZZUi0018-A) for spinocerebellar ataxia type 6 [289]. HEK293 cells for episodic ataxia with epilepsy [290]. Human neuronal cell line (SK-N-SH) and HEK293 cells for spinocerebellar ataxia type 6 [160]. HEK cells for spinocerebellar ataxia type 6 [291]. Cultured hippocampal neurons and HEK cells for migraine [292]. HEK-293 and PC12 cells for spinocerebellar ataxia type 6 [191]. SH‑SY5Y cells for progressive myoclonic epilepsy [161]	AAV9-mediated delivery of miR-3191-5p in mice rescued ataxia, motor deficits, and purkinje cell degeneration [165] MiRNA-3191-5p prevented the hyperacute disease in hyperacute model of spinocerebellar ataxia type 6 mice [226] SIS-RNAi could rescue spinocerebellar ataxia type 6 [160] Barbiturate pentobarbital could block Cav2.1 current [166] Acetazolamide had no effect on HEK cells carrying mutation for episodic ataxia type 2 [145]	Apoptotic pathway in progressive myoclonic epilepsy [161] Apoptotic pathway in ataxia [191]
*CACNA1C*	Calcium voltage-gated channel subunit alpha1 C	Cav1.2	L-type	None	Human induced pluripotent stem cell-derived cardiomyocyte (hiPSC-CM) for long-QT syndrome phenotype [162]. Induced pluripotent stem cell (iPSC) knockout resource for autism spectrum disorder [163]. Huh7 cells for severe fever with thrombocytopenia syndrome [171]. Pancreatic insulinoma RIN-m5f β-cells for type 2 diabetes mellitus [250]. SA-201 and HEK293 cells for sudden unexplained death in the young [293]. OCI-ly7, OCI-ly8, and OCI-ly3 DLBCL cell lines for lymphoma [172]. Hippocampal HT22 cells for affective disorders [156]. Induced pluripotent stem cell (iPSC) line for long QT syndrome type 8 [294, 295]. H9C2 and rat myocytes in long QT syndrome type 2 [296]. Timothy syndrome cardiomyocytes for cardiac arrhythmias [174]	Micro-RNA-137 targets the CACNA1C in mice and human neuroblastoma cells for Alzheimer's disease [167] MiR-221 and -222 inhibit Cav1.2 current in HL-1 cells [168] Nifedipine and benidipine hydrochloride inhibited severe fever with thrombocytopenia syndrome in Huh7 cells and in humanized mouse model [171] MiR-153 inhibitors upregulate the expression of Cacna1c mRNA and protein [169] Bay K8644 can prevent age related bone loss [173] MiR-103 can suppress expression of Cav1.2 and thus inhibit osteoblast proliferation [170] Estradiol can upregulates expression of Cav1.2 [148] Roscovitine could rescue cardiac arrhythmias [174] Stac2 and Stac3 can modulate CaV1.2 function [175] Azelnidipine reduces the expression of Cav1.2 [176]	Wnt pathway for age‐related osteoporosis [173] Apoptotic pathway in lymphoma [172] Apoptotic pathway in melanoma [192] Autophagic pathway in autism [197]
*CACNA1D*	Calcium voltage-gated channel subunit alpha1 D	Cav1.3	L-type	TsA-201 cells for ID and autism [82, 84]	TsA-201 cells in hearing [297]. Rat hippocampal and HEK293 cells for Alzheimer's disease [298]	Isradipine could block Cav1.3 current in vitro [84]	Apoptotic pathway [127]
*CACNA1E*	Calcium voltage-gated channel subunit alpha1 E	Cav2.3	R-type	TsA201 cells for ID and epilepsy [85]	None	Sipatrigine, eugenol, and lamotrigine could block Cav2.3 current [177, 178]	None
*CACNA1F*	Calcium voltage-gated channel subunit alpha1 F	Cav1.4	L-type	None	HEK293 and tsA-201 cells for congenital stationary night blindness type-2 [299]	None	None
*CACNA1G*	Calcium voltage-gated channel subunit alpha1 G	Cav3.1	T-type	None	COS-7 and rat islet cells for diabetes mellitus [300]. HEK293T cells for autosomal-dominant cerebellar ataxia [301]. Induced pluripotent stem cells (iPSCs) and HEK293T cells for spinocerebellar ataxia [302]	Endostatin, zonisamide, clozapine, roscovitine, mibefradil, iron and zinc can block Cav3.1 channel [179–185] Dearomatized isoprenylated acylphloroglucinol and monoterpenoid, hypatone A could rescue pathological gating properties for spinocerebellar ataxia 42 [186]	Autophagic pathway in melanoma cells [198] Apoptotic pathway in [193–195] Ras-ERK signaling pathway [200, 201]
*CACNA1H*	Calcium voltage-gated channel subunit alpha1 H	Cav3.2	T-type	None	TsA-201 cell for amyotrophic lateral sclerosis [303]	KYS-05090S can block Cav3.2 current [187]	Apoptotic pathway in myocardial cells [155]. Autophagy pathway [158]
*CACNA1I*	Calcium Voltage-Gated Channel Subunit Alpha1 I	Cav 3.3	T-type	HEK293T and mouse chromaffin cells for ID and epilepsy [74]	None	Zinc modulates Cav3.3 channel gating [181]	None
*CACNA2D1*	Calcium voltage-gated channel auxiliary subunit alpha2delta 1	Cav1.3	L-type	None	MKN74 cells (human gastric cancer cell line) [190]	MicroRNA-107 can inhibit expression of Cav1.3 in cancer [188, 189] Amlodipine can block Cav1.3 in cancer [190]	CXCR3/ERK1/2 signaling pathway [203] Ras/Raf/MEK/ERK signaling pathway [202]
*CACNA2D2*	Calcium voltage-gated channel auxiliary subunit alpha2delta 2	Cav1.3	L-type	None	LNCaP, DU145 and PC3 cell lines for prostate cancer [151]	None	Apoptotic pathway [196] Autophagic pathway [199]

## Discussion

Overall, this condition seems to be progressive, however, most primary authors provided less information on the course of the disease. Many of the reported cases with electrophysiological studies had gain-of- function variants. Severe to profound ID/GDD was more predominant for the cases with gain-of-function variants as compared to those with loss-of-function. *CACNA1E*, *CACNA1G*, *CACNA1F*, *CACNA2D2* and *CACNA1A* associated with more severe phenotype. The possible reasons as why these genes associated with more severe phenotype include (1) the neuronal location of the genes; all of them are located in the pre-synaptic membrane, (2) brain distribution; most of them are distributed in the brain cortex and/or hippocampus and/or cerebellum, (3) function of the genes; they all regulate the release of neurotransmitter, and (4) the effect of the variants; most of the reported variants in these genes had gain-of-function property. This review has also revealed some hotspots for future research.

## Summary of the clinical features

Calcium channels are widely spread in the human body (Table 1). Therefore, on top of brain, other organs such as eyes, heart, skeletal muscles, endocrine, and kidney can be affected too. In addition to ID/GDD, most cases with *CACNA1A* variants present with ataxia, epilepsy, attention deficit hyperactive disorder, autism spectrum disorder, dysmorphic features and eye abnormalities such as nystagmus, paroxysmal tonic upgaze, dysmetric saccades, blindness, myoclonus, ocular apraxia, exophthalmos and bilateral esotropia. Schizophrenia, anxiety, depression, hemiplegic migraine, coma, conductive deafness, vertigo attacks, dysarthria, tremors, athetosis, optic nerve glioma, abnormal behaviors such as aggression, sleeping problems can also be noticed. Cases carrying *CACNA1C* variants mostly present with Timothy syndrome, which is characterized by ID/GDD, autism, facial abnormalities, heart conditions such as atrioventricular block and patent ductus arteriosus, syndactyly and hypoglycemia. However, some cases can present with only ID/GDD, epilepsy, attention deficit hyperactive disorder plus congenital cardiac anomalies and dysmorphic features without autism. Cases with *CACNA1E* variants mostly present with profound ID/GDD accompanied with spastic dystonic quadriplegia, hypotonia, macrocephaly, and dystonia. ID/GDD, epilepsy, ataxia, and motor impairment, hypotonia, oculomotor apraxia, hyperopia, strabismus and multiple congenital anomalies can be seen for the cases carrying *CACNA1G* variants. Besides ID/GDD, epilepsy, autism, spastic quadriplegia, cortical blindness, lebers congenital amaurosis, klinefelters and retinitis pigmentosa, congenital nystagmus, rod cone dystrophy and myopia can be observed in those carrying *CACNA1F* mutations*.*

Cortical blindness, severe proximal muscular hypotonia, distal muscular hypertonia, epilepsy and ID/GDD can be noticed for the cases carrying *CACNA1I* variants. ID/GDD and ventral septal defect are the major clinical features for the cases with *CACNA1H* mutations. Dyskinesia such as choreiform movements, erratic limb movements, tremor, restlessness, sleep disturbance, dysmorphic features, oculo-motor apraxia, strabismus, nystagmus, axial and leg hypertonia, head tonic extension, brisk symmetric reflexes, hyperglycemia, glycosuria, and epilepsy are additional clinical features that can be observed for the cases with ID/GDD and yet carrying *CACNA2D2* variants. Whereas, for the *CACNA2D1* variants, epilepsy, autism, attention deficit hyperactive disorder, ataxia, facial dysmorphism, clinodactyly, brachymetacarpy, abnormal skin, short stature, transient diabetes with hyperinsulinemia, hearing impairment, aggressiveness, agitation, stereotypic hand movements, primary aldosteronism, heart defects and hypotonia can be seen in additional to ID/GDD.

Brain malformations including cerebellar, cortical and optic nerve atrophy were common in all ID-related calcium channelopathies. For two cases with gain-of-functions variants and underwent muscle biopsy showed evidence of mitochondrial dysfunction.

### The pathomechanisms

In normal physiological conditions, calcium ions enter neurons via calcium channels (Cav1, Cav2 and Cav3). Most of the calcium ions enter mitochondria for ATP synthesis, which is crucial for synaptic plasticity. The remaining calcium ions in the cytosol stimulate transcription, facilitate release of neurotransmitters, promote neurite outgrowth, and activate some enzymes, which are important for synaptic plasticity.

#### Calcium effects on synapses

The calcium channel Cav1.2, which is encoded by *CACNA1C,* regulates gene expression by activating the cyclic adenosine monophosphate (cAMP) response element-binding protein (CREB) and brain-derived neurotrophic factor (BDNF), both of which are essential for long-term potentiation [88, 89]. The increased expression of Cavα2δ subunit encoded by *CACNA2D1* facilitates synapses to make more efficient use of calcium influx to activate neurotransmitter release [90]. In addition, this subunit interacts with big potassium (BK) channels and N-methyl-D-aspartate receptors (NMDARs) [91]. Cav2 channels including Cav2.1 and Cav2.3 encoded by *CACNA1A* and *CACNA1E* genes, respectively, form large signalling complexes in the presynaptic nerve terminal, which regulate the calcium entry and in turn facilitate neurotransmitter release and short-term plasticity [92, 93]. There are more than 100 proteins, which interact with Cav2.1 and Cav2.2 channels in presynaptic terminals and are involved in the release of neurotransmitters [92]. Cav1 channels including Cav1.2, Cav1.3, and Cav1.4 encoded by *CACNA1C*, *CACNA1D,* and *CACNA1F*, respectively, form signalling complexes in postsynaptic dendrites as well as dendritic spines, in which calcium entry induces long-term plasticity [92]. Cav3.1 channel that is encoded by *CACNA1G* is responsible for postsynaptic calcium signaling too and thus contribute to long-term potentiation [94, 95]. They may enhance dendritic depolarization or, on the other hand, can stimulate calcium-activated potassium currents, resulting in membrane hyperpolarizations [96]. The co-activation of Cav3.3 and GluN2B-containing NMDA receptors mediates long-term potentiation at thalamoreticular inputs [97].

Cav2.1 current facilitates short term synaptic plasticity through activation of neuronal calcium sensor proteins (CaS) [92, 98]. It is hypothesised that short-term plasticity is regulated by the SNARE complex that acts as the effector of synaptic vesicle exocytosis [99]. SNARE complex is comprised of SNAP-25, syntaxin, and synaptobrevin, also known as VAMP [100]. These proteins interact with the synaptic protein interaction (synprint) site present on the Cav2.1 calcium channels [101]. In addition, synaptotagmin 1 and 7 (calcium sensor protein), which regulates SNARE function has been implicated in short-term synaptic plasticity [102–104]. Cav3.2 current are responsible for retrieval of memory [105], and plays a major role in short-term plasticity [106]. Mutations in the identified calcium genes related to ID/GDD can affect neurotransmitter release [107–111].

#### Calcium effects on learning and long-term potentiation

Long-term potentiation occurs in two phases. Phase 1 includes increase in the expression of alpha-amino-3-hydroxy-5-methyl-4-isoxazolepropionic acid receptors (AMPAR) while phase 2 involves activation of transcription and protein synthesis (formation of more AMPARs and dendrites for new synapse formation via secretion of growth factors) [112]. Glutamate released from the presynaptic membrane binds to the AMPARs and NMDARs, which are present on the postsynaptic membrane [112]. The binding of glutamate to AMPARs allows sodium ions influx leading to membrane depolarization [112]. When the membrane is depolarized, the magnesium blockage in NMDARs is removed, thereby, allowing the calcium ions to enter into the cell [112]. Calcium influx through NMDARs leads to calmodulin-dependent activation of Ca^2+^/calmodulin-dependent protein kinase (CaMKII)[112] and Kv4.2 internalization [113]. The activation of CaMKII leads to a rapid surge in the number of AMPARs at synapses [112]. In addition, CaMKII phosphorylates major and auxiliary subunits of AMPARs [114] including serine 831, the carboxyl-terminal of GluA1 [115] stargazin, and transmembrane AMPAR regulatory proteins (TARPs) [116]. CaMKII also plays a role in expanding and consolidating the synapse [114]. The CaMKII/NMDAR complex acts like a switch that regulates synaptic strength [117, 118]. Synaptic plasticity is chiefly facilitated by variations in the number of synaptic AMPARs, which are regulated by auxiliary subunits (stargazin and TARPs) that control channel gating and AMPAR trafficking [116]. Stargazin controls both AMPAR function and calcium channels [116, 119] and its dysfunction affects long-term potentiation [120].

Cav1 channels form signalling complexes in postsynaptic dendrites as well as in dendritic spines, in which calcium influx induces long-term potentiation [92]. CaMKII plays a role in activating the calcium-regulated protein kinase (CaMKIV), which in turn activates the transcription factor CREB [121]. The influx of calcium via Cav1.2 channels leads to transcription, translation, and consequently protein synthesis [122] leading to memory stabilisation [123]. Protein synthesis is very crucial for long-term memory as its inhibitors such as anisomycin, puromycin, acetoxycycloheximide, and cycloheximide were shown to affect long term potential and not initial attainment of task [124]. Beta-2 adrenergic receptors interact with Cav1.2 channels to control the long-term postsynaptic plasticity and the activity of the calcium channel [125, 126]. The Cav3.3 interacts with GluN2B-containing NMDA receptors to induce long-term potentiation [97].

#### Calcium channels and mitochondria

In this study, we found the underlying mechanisms for the reported variants included gain- and/ or loss-of-function, alteration in kinetics (activation, inactivation) and dominant-negative effects of truncated forms of alpha1 subunits. The contribution of gain- and loss-of-function variants to ID might be attributed to the mitochondrial dysfunction [127]. Mitochondria not only depend on calcium releasing sites such as endoplasmic reticulum (ER) but also interact with calcium channels (Cav2) present on the plasma membrane. Calcium ions can enter through the calcium channels when there is depletion of ER calcium stores [16]. When the cell is activated, there is high accumulation of calcium ions in the mitochondria as compared to the cytosol [15].

Despite the fact that mitochondria are responsible for production of energy, they also regulate cellular signalling (calcium signalling), cell defence, and cell death [16]. Besides, mitochondrial calcium regulates calcium currents in the cell for signalling process, ATP synthesis, and initiation of cell death. Thus, mitochondrial function and calcium homeostasis are entwined processes that regulate each other [16]. Noteworthy, ATP is important for regulation of membrane excitability, synaptic transmission, transcription, and apoptosis [15]. Neurons require ATP to carry out their activity at synapse and the mitochondrial calcium uniporter (MCU) present in these cells allows calcium ions to enter neurons for different activities [15]. Calcium ions can activate the release of BDNF for growth and repair of neurons [128]. Mitochondria can affect synaptic plasticity in several ways; in conjunction with BDNF, they can supply ATP for synaptic connections and they can prune those connections away [128]. Furthermore, mitochondria can also produce neurosteroids that can determine how calcium ions enter the neuron [129].

**Fig. 9:**
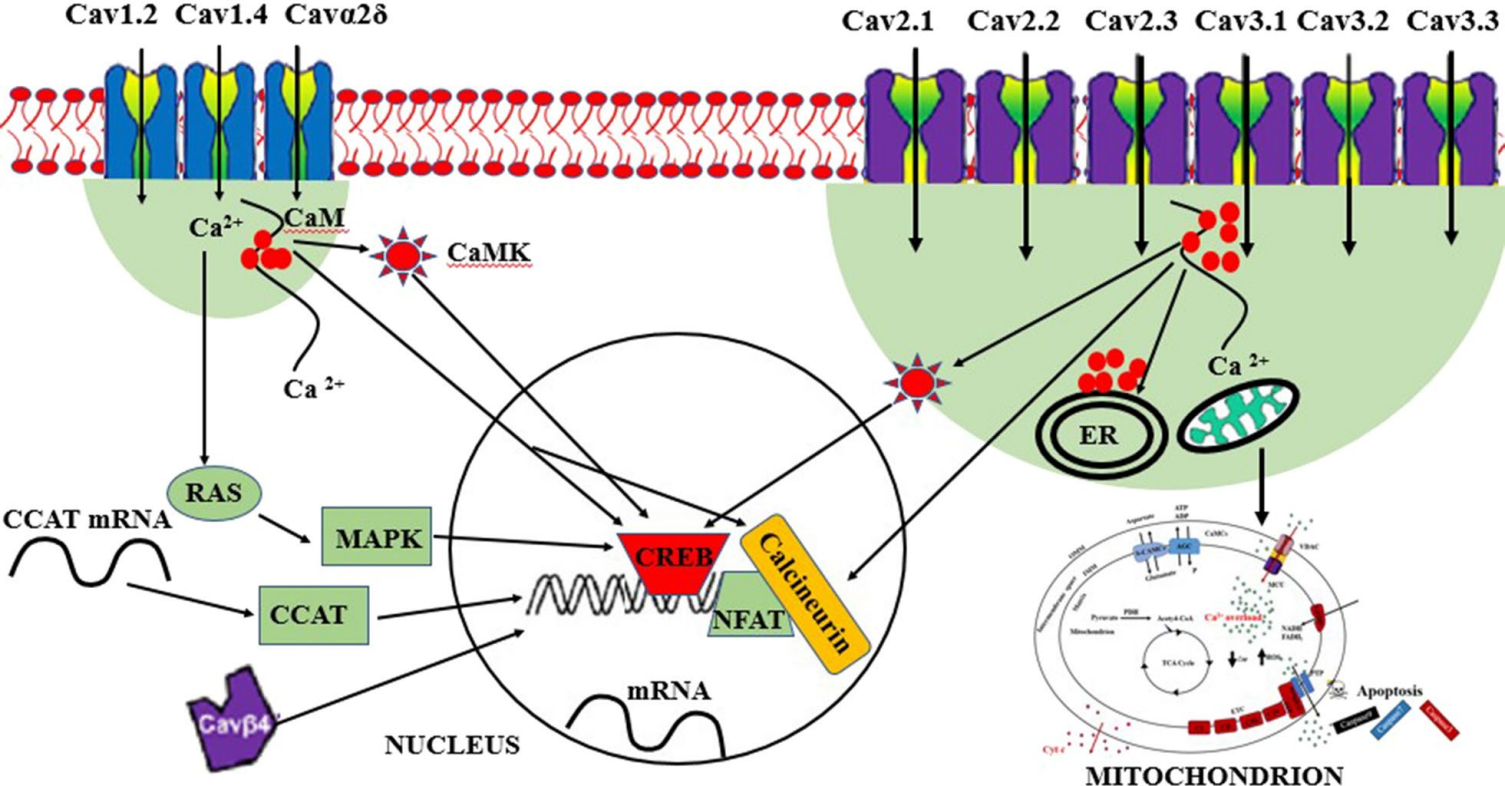
The mechanism of how gain-of-function variants can lead to ID/GDD. Calcium ions can enter into neuronal cell via Cav1.2, Cav1.4, Cavα2δ, Cav2.1, Cav2.2, Cav2.3, Cav3.1, Cav3.2 and Cav3.3. In normal physiology, some of the calcium ions go to the nucleus to initiate gene transcription, translation and protein synthesis essential for learning and memory, some go to mitochondria for ATP synthesis (essential for learning and memory) and some to the endoplasmic reticulum (ER) for storage. Gain-of- function variants can allow excessive influx of calcium ions inside the cells. This will reduce the amount of ATP production while contributing to the accumulation of reactive oxygen species (ROS) and release of cytochrome C that induces apoptosis of neuronal cell. Many red and blue solid circles stand for high calcium levels

**Fig. 10:**
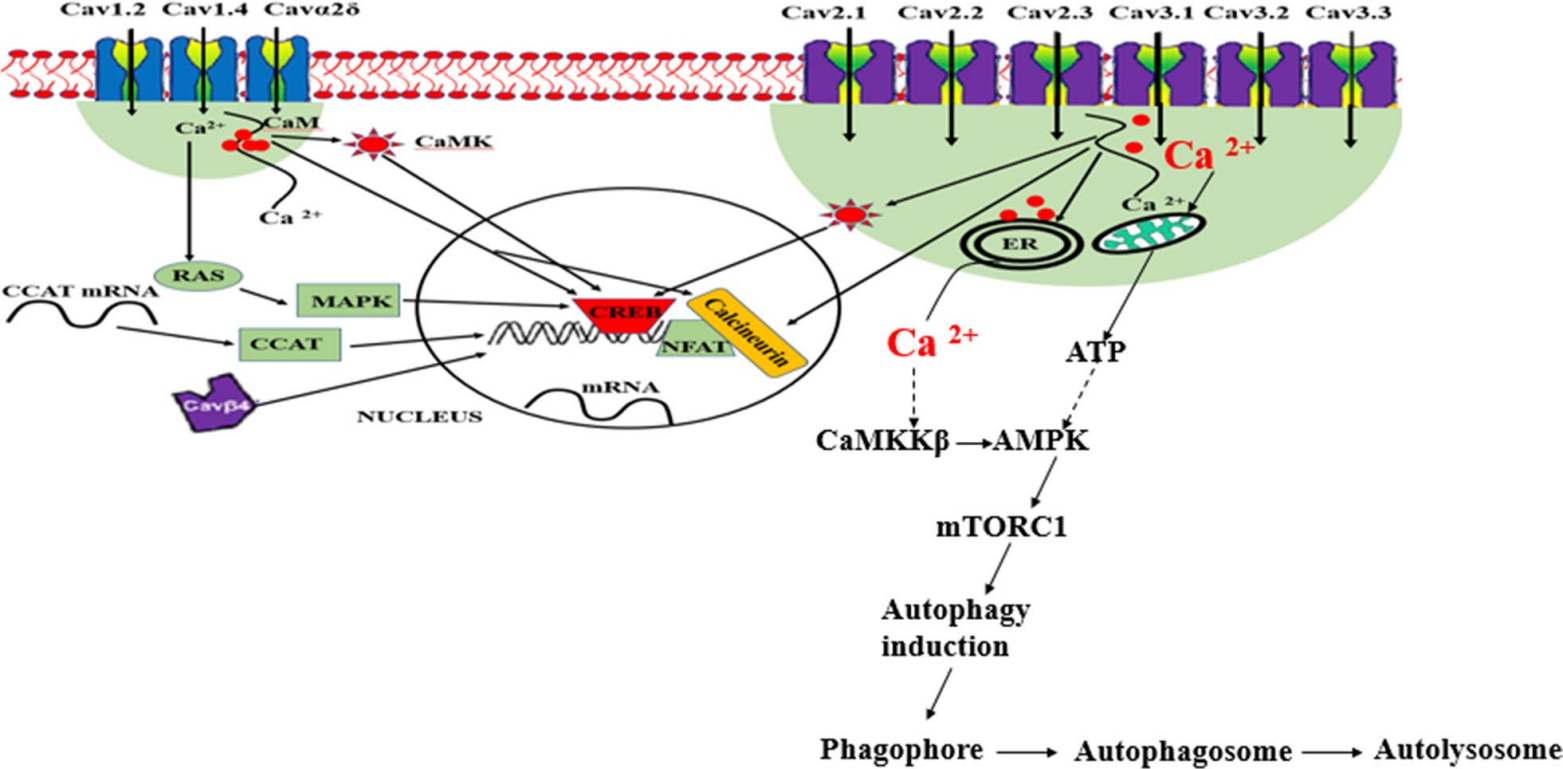
A summary of how loss-of- function variants can lead to autophagy. Calcium ions can enter into neuronal cell via Cav1.2, Cav1.4, Cavα2δ, Cav2.1, Cav2.2, Cav2.3, Cav3.1, Cav3.2 and Cav3.3. In normal physiology, some of the calcium ions go to the nucleus to initiate gene transcription, translation and protein synthesis essential for learning and memory, some go to mitochondria for ATP synthesis (essential for learning and memory) and some to the endoplasmic reticulum for storage. Calcium stored in the endoplasmic reticulum (ER) is used when there is minimal/no influx of calcium ions inside the cells. Autophagy occurs when there is metabolic stress such as low ATP and nutrient starvation. Low levels of calcium ions inside the neuronal cell being due to loss-of- function of calcium channels or due to depletion in ER can activate autophagy pathway. Low calcium entrance in the mitochondria will lead to low production of ATP which will activate the AMP-activated protein kinase (AMPK, a sensor of energy levels) and mTOR complex 1 (mTORC1) which in turn induce autophagy. Likewise, low calcium levels from the ER can activate calmodulin-dependent protein kinase kinase β (CaMKKβ) and then AMPK leading to autophagy. Dotted arrows signify low levels. Few red solid circles stand for low calcium ions levels

Calcium overload in the mitochondria activates apoptotic cascade leading to cell death [130, 131]. Figure 9 summarizes the mechanism. Conversely, low levels of calcium in mitochondria can induce autophagy [132]. Figure 10 summarizes the mechanism. Moreover, excessive accumulation of calcium ions in the mitochondria of the neuron can lead to excessive neuronal firing like in epileptic seizure, thereby, leading to neuronal death [129]. Mitochondrial calcium buffering is very important and its impairment can cause several diseases. For example, calcium overload due to failure of the mitochondrial buffering system in skeletal muscles leads to amyotrophic lateral sclerosis [133]. In summary, excitable cells including neurons require proper regulation of calcium and mitochondrial homeostasis. Whereas diminished mitochondrial calcium influx can result in loss of neuronal function, excessive mitochondrial calcium can induce neuronal damage and death. Mitochondria supply energy for proper brain functioning, enhancing synaptic plasticity, production of hormones and signalling molecules, and regulating neurotransmitters, and its dysfunction can lead to several diseases including ID [129]. Thus, we speculate that gain-of-function variants can induce neuronal apoptosis (Fig. 9), whereas, loss-of-function mutations can activate autophagy (Fig. 10) resulting in ID. Nevertheless, this hypothesis needs further studies for confirmation. Noteworthy, for the few cases that received muscle biopsy tests, evidence of mitochondrial dysfunction was noticed for two cases carrying gain-of-function variants; decreased mitochondrial complex I and III activity for the case with *CACNA1C* variant [73] and partial deficits in complexes II and III for the case carrying *CACNA1A* variant [63].

Excessive calcium influx through NMDARs can induce neurotoxicity via activation of neuronal nitric oxide synthase (nNOS) [127] and neuronal NADPH oxidase-2 (NOX2) pathways [134]. Excessive mitochondrial accumulation of calcium ions in neurons can lead to excessive neuronal hyperexcitabilty, similar to that observed in epilepsy, thus, leading to neuronal death [129].

#### Calcium channels and epilepsy

We found that more than half of the reported cases had concomitant epilepsy or epileptic encephalopathy. It is unclear whether epileptic activity played a role in the development of ID. Rodent studies have shown that epileptic encephalopathies, frequent seizures, and/or interictal epileptic discharges can lead to synaptic reorganization, abnormal neurogenesis, or disruption of the developing neural circuits, which can cause ID [135]. However, some of the underlying aetiologies for epilepsy can also cause ID independently [135, 136]. Noteworthy, some of the antiepileptic drugs can exacerbate ID [135, 136]. A previous study on sodium channelopathy (*SCN1A* alleles) revealed that seizure frequency and electroencephalography abnormalities do not correlate with the degree of ID and behavioural disturbances [137]. Besides, *Scn1a* knockout mice demonstrated cognitive impairment even without seizures [138]. However, no similar study has been performed on calcium channelopathies. Based on our present knowledge about the existing cerebro-cerebellar circuits [139] we speculate that ID and EP might occur independently. Calcium channelopathies can impair the development of the cerebellum leading to abnormalities in different cerebro-cerebellar circuits accounting for ID and EP separately.

#### Calcium channels and cerebellum

The cerebellum plays a major role in the planning and execution of movement as well as in language and attention [140]. In our study, cerebellar atrophy was observed in 25% of the retrieved cases. There are important cerebro-cerebellar circuits responsible for learning and memory [139]. Calcium channels are crucial for the development of the brain including the cerebellum [23]. Therefore, calcium channelopathies can cause cerebellar morphological changes leading to several neurodevelopmental disorders including ID, autism, epilepsy, and attention deficit hyperactivity disorder [139, 141]. Abnormal cerebellar development and/or early cerebellar damage can affect behaviour via the closed-loop circuits connecting the cerebellum with multiple areas in the cerebral cortex. Behavioural changes depend on the affected cerebro-cerebellar circuits [139]. Six out of 12 cases with *CACNA1A* variants and ID showed statistically significant association with cerebellar atrophy according to one study [45]. Besides, only two cases in that study presented with epilepsy, thus, questioning the role of epilepsy in developing ID for the cases with *CACNA1A* variants [45]. In another study, abnormal cerebellar development caused by *CACNA1G* alleles was hypothesised as the possible cause of cognitive impairment [23]. A rodent study demonstrated the participation of the cerebellum in cognitive function [142]. Our review consolidates evidence for the theory that the cerebellum might be involved in learning and memory, thus, supporting our speculation that calcium channelopathies lead to cerebellar atrophy that can cause ID via abnormal cerebro-cerebellar circuits.

Noteworthy, conventional MRI was used as a method to detect abnormalities for the identified cases. Thus, there is a possibility that other cases were missed due to the limitation of this modality. Advanced imaging methods including the fMRI and H-MRS revealed brain abnormalities (including metabolic changes) that could not be detected with conventional MRI for the cases diagnosed with temporal lobe epilepsy and autism [31, 32], NGD [33], and minimal hepatic encephalopathy with liver cirrhosis [34]. Besides, there was a correlation between choline/creatine ratio and cognitive deficits and genotypes for the cases diagnosed with NGD [33]. Furthermore, abnormal cognitive results correlated with apparent diffusion coefficient value and metabolic changes for the children diagnosed with minimal hepatic encephalopathy with liver cirrhosis signifying that altered metabolic changes and cerebral edema were responsible for cognitive changes [34]. Consequently, fMRI and H-MRS imaging modalities are recommended for the ID/GDD cases. Early detection of metabolic changes can aid clinicians to minimize cognitive decline.

### Available animal models, modulators, and pathways for ID/GDD related to calcium channels defects

A comprehensive review of literature was also carried out for animal models of all reported calcium channel genes related to ID. Unfortunately, only a few calcium channel genes have animal models for ID**.** Many authors focused more to study migraine, ataxia and epilepsy through animal models. There are several interventions used for aforementioned conditions with available animal models. For instance, seizures were inhibited by ethosuximide and valproic acid in Cacna1a Tottering-6j mice [215] and Cacna1a-mutant GRY rat [143]. Acetazolamide could abolish stress-induced ataxia in Cacna1a mice [144] but had no effect on HEK cells carrying mutation for episodic ataxia type 2 [145]. Cav1.2 current could be inhibited by nifedipine [146], diltiazem [147] and miR-135b [148]. Niflumic acid could block Cav3.3 current [149] while 6-prenylnaringenin could block Cav3.2 current [150]. Gabapentin is a ligand of Cav1.3 [151]. The commonest affected pathway for the available studies is the apoptotic pathway [152–155] followed by oxidative stress pathway [156, 157] and autophagy [158]. More details can be found in Table 2. Therefore, we argue future studies on ID/GDD involving animal models to be conducted. Moreover, studies focused on treatments aiming to mitigate cognitive impairment should be carried out.

### Available other functional models, modulators and pathways for ID/GDD related to calcium channels defects

Upon intensive review of literature, we found that tsA-201, HEK293T and mouse chromaffin cells are being used to study ID, epilepsy and autism [74, 82, 84, 85]. Besides, SH-SY5Y human neuroblastoma cells was used for familial hemiplegic migraine type 1 and episodic ataxia [159, 160] and progressive myoclonic epilepsy [161]. Human induced pluripotent stem cell-derived cardiomyocyte (hiPSC-CM) was used for long-QT syndrome phenotype [162]. Induced pluripotent stem cell (iPSC) knockout resource was used for autism spectrum disorder [163]. COS-7 cells was used for spinocerebellar ataxia type 2 [164]. Modulators for Cav2.1 current in functional models include miR-3191-5p [165], SIS-RNAi [160] and barbiturate pentobarbital [166]. Cav1.2 current can be modulated by micro-RNA-137, -221, -153, -103 and -222 [167–170], nifedipine and benidipine hydrochloride [171], rituximab [172], bay K8644 [173], estradiol [148], roscovitine [174], stac2 and stac3 [175] and azelnidipine [176]. Sipatrigine, eugenol, and lamotrigine can block Cav2.3 current [177, 178]. Endostatin, zonisamide, clozapine, roscovitine, mibefradil, iron and zinc can block Cav3.1 channels [179–185]. Dearomatized isoprenylated acylphloroglucinol and monoterpenoid, hypatone A could rescue pathological gating properties for spinocerebellar ataxia 42 [186]. KYS-05090S could block Cav3.2 current [187]. Cav1.3 current could be blocked by microRNA-107 [188, 189] and amlodipine [190]. The commonest affected pathway is apoptotic pathway [127, 155, 161, 172, 191–196] followed by autophagic pathway [158, 197–199], Ras/Raf/MEK-ERK signaling pathway [200–203] and Wnt pathway [173]. Further details can be found in Table 3.

### Evaluation and investigations

The aforementioned clinical features (multisystem abnormalities) should guide clinicians in suspecting mutations in calcium genes. In addition, progressive cerebellar, cerebral and optic atrophy in the brain imaging are important clues. Therefore, we recommend brain imaging and next generation sequencing diagnostic methods to be used whenever there is a suspicion. Since there is an evidence of many CNVs encompassing calcium genes and yet relate to ID/GDD, we suggest microarray tests to be considered for the cases that present with multiple congenital anomalies. We recommend intensive metabolic tests in urine, blood and cerebrospinal fluid. Those tests can check the levels of pyruvate, lactic acid and others as per consensus based expert recommendations for evaluation of mitochondrial disease [204]. Molecular genetic tests for mitochondria can be carried out but muscle biopsy is recommended even if the results becomes negative. Tests that can detect biochemical signs of neurotransmitter abnormalities are recommended. Advanced neuroimaging modalities including fMRI and H-MRS are recommended.

### Treatments

Studies on treatment focusing on calcium channelopathies for ID/GDD are not available. Available studies focused more on epilepsy, ataxia and migraine, and those drugs do not have beneficial effects on cognition. Verapamil and acetazolamide are good for migraine [205]. Acetazolamide has been reported to be effective for ataxia to some cases [206] and ineffective for others [207]. Ethosuximide and valproic acid are effective for absence epilepsy [208]. Otherwise, there are some treatable ID/GDD, especially those caused by inborn errors of metabolisms (n = 81). These include 19 disorders of organic acids, lysosomes (n = 12), amino acids (n = 12), hyperhomocysteinemia (n = 7), vitamins/co-factors (n = 8), urea cycle (n = 7), neurotransmission (n = 7), creatine (n = 3), cholesterol and bile acid (n = 2), fatty aldehydes (n = 1), glucose homeostasis and transport (n = 2), metals (n = 3), mitochondria (n = 2), peroxisomes (n = 1), and pyrimidines (n = 2)[209]. Diazoxide choline controlled-release tablets have been reported to be useful in controlling hyperphagia, obesity and aggressive behaviors in cases with Prader-Willi syndrome [210, 211]. This drug has also been reported to prevent the aggravation of the pre-existing ID/GDD for the cases with syndromes known to be accompanied with hyperinsulinaemic hypoglycaemia such as Beckwith-Wiedemann, Sotos, Kabuki and Turner [212]. The early initiation of the diazoxide within 3 months of the onset of symptoms is associated with normal intelligence [213].

### Intervention strategies

Upcoming studies should focus on developing animal models for ID/GDD aiming to explore the possible underlying mechanisms for ID/GDD and possible treatment options. If possible, future studies should focus on identifying the effect of calcium blockers and openers both in vivo and in vitro. Additionally, future studies should focus on exploring the relationship between calcium channelopathies, mitochondria, and ID/GDD as well as the role of cerebellar morphological changes in ID/GDD. Lastly, future studies can explore whether epileptic activity in calcium channelopathies can cause ID/GDD or whether ID/GDD and epilepsy occur independently. Expanding the understanding of mechanisms underlying the development of ID/GDD will help to improve the treatment strategies for ID/GDD.

### Comparison of our review with other reviews

Previous narrative reviews summarised the relationship between calcium channelopathies and epilepsy as well as autism spectrum disorder [17, 35–37]. To the best of our knowledge, this is the first systematic review to explore the relationship between calcium channelopathies and ID/GDD. Our review has revealed variations in ten genes that relate to ID/GDD including *CACNA1A, CACNA1C*, *CACNA1I, CACNA1H, CACNA1D*, *CACNA2D1*, *CACNA2D2*, *CACNA1E*, *CACNA1F*, and *CACNA1G*. Most variants exhibited gain-of-function effect. Severe to profound ID/GDD was observed more for the cases with gain-of-function variants as compared to those with loss-of-function. *CACNA1E*, *CACNA1G*, *CACNA1F*, *CACNA2D2* and *CACNA1A* associated with more severe phenotype. In another review, both gain- and loss-of-function variants in *CACNA1A*, gain-of-function variants in *CACNA1H****,*** and variants in *CACNA1G* were linked to epilepsy [35, 36, 214]. Calcium overload resulting to mitochondrial dysfunction, oxidative stress, and cell damage was concluded as a possible pathomechanism important for the development of acquired epilepsies [36]. In the review regarding autism spectrum disorder, *CACNA1A*, *CACNA1B*, and *CACNA1C* (gain-of-function), *CACNA1D* (gain-of-function), *CACNA1E* and *CACNA1F* (gain-of-function), *CACNA1G* and *CACNA1H* (loss-of-function), *CACNA1I*, *CACNB1*, and *CACNB2* (gain-of-function effect) as well as *CACNA2D3* and *CACNA2D4* (loss-of-function effect) were reported as candidate genes [37]. Our review has highlighted *CACNA1C*, *CACNA1F*, *CACNA1I*, *CACNA2D1* and *CACNA2D2* as additional genes for epilepsy, and *CACNA2D1* for autism spectrum disorder. Furthermore, our review has revealed *CACNA1A*, *CACNA1C* and *CACNA2D1* as the candidate genes for attention deficit hyperactive disorder.

### Merits of the study

This review has revealed that calcium channelopathies contribute to the development of ID/GDD. Variations in 10 genes that relate to ID/GDD including *CACNA1A, CACNA1C*, *CACNA1I, CACNA1H, CACNA1D*, *CACNA2D1*, *CACNA2D2*, *CACNA1E*, *CACNA1F*, and *CACNA1G* were found, and most variants exhibited gain-of-function effect. It has unveiled that severe to profound ID/GDD is observed more for the cases with gain-of-function variants as compared to those with loss-of-function. Notably, *CACNA1E*, *CACNA1G*, *CACNA1F*, *CACNA2D2* and *CACNA1A* correlated with more severe phenotype. Our review has further revealed variants in *CACNA1C*, *CACNA1F*, *CACNA1I*, *CACNA2D1* and *CACNA2D2* as additional genes related to epilepsy. Besides, *CACNA2D1* is related to autism spectrum disorder while *CACNA1A*, *CACNA1C* and *CACNA2D1* are candidate genes for attention deficit hyperactive disorder. Our study has showed the existence of the relationship between calcium channelopathies, mitochondria dysfunction, cerebellar morphological changes, and ID/GDD. We have summarized the information related to available animal, and functional cell models, modulators, and pathways, evaluation, investigations, treatments and intervention strategies for ID/GDD related to calcium channels defects. Our review will help future studies on the mechanisms of ID/GDD to develop novel treatment strategies for this condition.

### Study limitations

Our study has several limitations. We could not discuss the relationship between ID/GDD and other channelopathies (sodium, potassium and chloride) in detail, as the breadth of content that a review of that scope would provide exceeds the capacity of one article. There is no advanced neuroimaging modalities including fMRI and H-MRS were done for the reported cases; therefore, it is difficult to comment on brain metabolic changes.

### Conclusions

In summary, calcium channelopathies can cause ID/GDD. There is a scarcity of animal studies on the mechanisms of ID/GDD in relation to calcium channelopathies. Studies on treatment options for cognitive impairment are lacking. The underlying mechanisms for the reported variants include gain- and/ or loss-of-function, alteration in kinetics (activation, inactivation) and dominant-negative effects of truncated forms of alpha1 subunits. While both gain- and loss-of-function variants are associated with ID/GDD, the mechanisms underlying their involvement are unclear.

## Supplementary Information

**Additional file 1.** Search strategies utilized.

**Additional file 2: Table S1.** Specific calcium channel genes, their mutations, OMIM number, functional significance, protein/enzyme activity change, type of change, phenotype, electrophysiology results, and MRI results.

**Additional file 3.**

## Data Availability

All data generated or analysed during this study are included in this published article [and its supplementary information files.

## Abbreviations

cAMP: Cyclic adenosine monophosphate
ID: Intellectual disability
GDD: Global developmental delay
HVA: High voltage activated
LVA: Low voltage activated
*CACNA1A*: Calcium voltage-gated channel subunit alpha1 A
*CACNA1B*: Calcium voltage-gated channel subunit alpha1 B
CACNB1: Calcium voltage-gated channel auxiliary subunit beta 1
CACNB2: Calcium voltage-gated channel auxiliary subunit beta 1
CACNA2D3: Calcium voltage-gated channel auxiliary subunit alpha2delta 3
CACNA2D4: Calcium voltage-gated channel auxiliary subunit alpha2delta 4
*CACNA1C*: Calcium voltage-gated channel subunit alpha1 C
*CACNA1D*: Calcium voltage-gated channel subunit alpha1 D
*CACNA1E*: Calcium voltage-gated channel subunit alpha1 E
*CACNA1F*: Calcium voltage-gated channel subunit alpha1 F
*CACNA1H*: Calcium voltage-gated channel subunit alpha1 H
*CACNA1I*: Calcium voltage-gated channel subunit alpha1 I
CACNA1G: Calcium voltage-gated channel subunit alpha1
*CACNA2D1*: Calcium voltage-gated channel auxiliary subunit alpha2delta 1
*CACNA2D2*: Calcium voltage-gated channel auxiliary subunit alpha2delta 2
CREB: Cyclic adenosine monophosphate (cAMP) response element-binding protein
BDNF: Brain-derived neurotrophic factor
BK: Big potassium
NMDARs: N-methyl-D-aspartate receptors
AMPAR: Alpha-amino-3-hydroxy-5-methyl-4-isoxazolepropionic acid receptors
CaMKII: Calmodulin-dependent activation of Ca^2^^+^/calmodulin-dependent protein kinase II
nNOS: Neuronal nitric oxide synthase
NOX2: Neuronal NADPH oxidase-2
MCU: Mitochondrial calcium uniporter
