# Supplementary material for: Calcium channelopathies and intellectual disability: a systematic review
Source: Orphanet J Rare Dis. 2021 May 13;16:219. doi: 10.1186/s13023-021-01850-0 (PMC8120735; doi:10.1186/s13023-021-01850-0)
Supplement: Supplementary file 1 — Additional file 1. Search strategies utilized. [file 13023_2021_1850_MOESM1_ESM.docx]

**Search strategies which were used.**

**PubMed**

1. MeSH Terms: calcium channels; intellectual disability

Additional terms: intellectual; intellectual disability; channel; channels; calcium; disability; calcium channel; calcium channels.

1. MeSH Terms: calcium channels; intellectual disability

Additional terms: intellectual; intellectual disability; calcium channels; mental; calcium channel; retardation; mental retardation; calcium; channel; channels; disability

1. MeSH Terms: calcium channels; internationality

Additional terms: calcium; globals; globalism; globalize; delaying; developmental; globalizes; internationality; globalizing; delays; calcium channels; delay; global; globalization; globalized; delayed; developmentally; globally; channels

1. MeSH Terms: calcium channel

Additional terms: channels; calcium; delay; delaying; delays; calcium channels; calcium channel; channel; developmental; developmentally; delayed

**EMBASE**

1. ('calcium channel'/exp OR 'calcium channel' OR (('calcium'/exp OR calcium) AND channel)) AND ('intellectual disability'/exp OR 'intellectual disability' OR (intellectual AND ('disability'/exp OR disability)))
2. ('calcium channel'/exp OR 'calcium channel' OR (('calcium'/exp OR calcium) AND channel)) AND ('mental retardation'/exp OR 'mental retardation')
3. ('calcium channels'/exp OR 'calcium channels' OR (('calcium'/exp OR calcium) AND channels)) AND ('global developmental delay'/exp OR 'global developmental delay' OR (global AND developmental AND delay))
4. ('calcium channel'/exp OR 'calcium channel' OR (('calcium'/exp OR calcium) AND channel)) AND ('developmental delay'/exp OR 'developmental delay')
