# Supplementary material for: Calcium channelopathies and intellectual disability: a systematic review
Source: Orphanet J Rare Dis. 2021 May 13;16:219. doi: 10.1186/s13023-021-01850-0 (PMC8120735; doi:10.1186/s13023-021-01850-0)
Supplement: Supplementary file 2 — Additional file 2: Table S1. Specific calcium channel genes, their mutations, OMIM number, functional significance, protein/enzyme activity change, type of change, phenotype, electrophysiology results, and MRI results. [file 13023_2021_1850_MOESM2_ESM.docx]

**Additional file 2: Table S1. Calcium channel genes reported to associate with global developmental delay/ intellectual disability**

| **Names of the gene** | **Syndrome/**  **phenotype** | **Other clinical features/ organs affected** | **OMIM number** | **Inheritance/nucleotide or protein change** | **Type of mutation** | **Altered protein function** | **Silico modeling prediction/ACMG classification** | **MRI results** | **Biochemical signs of mitochondrial/ neurotransmitter abnormalities** | **Severity of the GDD/ID** | **Disease course** | **Reference** |
| --- | --- | --- | --- | --- | --- | --- | --- | --- | --- | --- | --- | --- |
| *CACNA1A* | ID, EP, ataxia and headache | Nystagmus | 601011 | De novo  Ile712Val | Missense | Unknown | Pathogenic | Cerebellar and cerebral atrophy | Negative metabolic tests | Severe ID | Progressive | ^1^ |
| *CACNA1A* | EIEE | Not clear | 601011 | De novo  p.Val1812Ala | Missense | Unknown | Pathogenic | Unknown | Not clear | Mild ID | Not clear | ^2^ |
| *CACNA1A* | ID and episodic ataxia | Psychotic symptoms | 601011 | Not clear  p.Arg822Profs*246 | Nonsense | Unknown | Deleterious | Unknown | Not clear | ID | Not clear | ^3^ |
| *CACNA1A* | ID and episodic ataxia | None | 601011 | Inherited (mother)c.3698+1Gly>Ala | Aberrant splicing | Unknown | Pathogenic | Unknown | Nor clear | ID | Not clear | ^3^ |
| *CACNA1A* | ID and episodic ataxia | None | 601011 | Inherited (mother)c.3698+1Gly>Ala | Aberrant splicing | Unknown | Pathogenic | Unknown | Not clear | ID | Not clear | ^3^ |
| *CACNA1A* | ID, cerebellar ataxia and migraine | Depression | 601011 | Not done  p.Val581Leu | Missense | Unknown | Pathogenic | Unknown | Not done | ID | Progressive | ^4^ |
| *CACNA1A* | GDD, congenital ataxia and hemiplegic migraine with cerebral edema. | None | 601011 | De novo  p.Phe1502del | Deletion | Hyperpolarizing shift in the voltage dependent activation | Pathogenic | Cerebellar and cerebral atrophy | Partial deficits in complexes II, III and IV were found in the muscles. | GDD | Progressive | ^5^ |
| *CACNA1A* | ID, EP, prolonged attacks of migraine with hemiplegia, ataxia, and coma | None | 601011 | De novo  Tyr1385Cys | Missense | Unknown | Deleterious, | Cerebellar and cerebral atrophy | Negative metabolic tests | ID | Progressive | ^6^ |
| **Names of the gene** | **Syndrome/**  **phenotype** | **Other clinical features/ organs affected** | **OMIM numbers** | **Inheritance/Nucleotide or Protein change** | **Type of mutation** | **Altered protein function** | **Silico modeling prediction/ACMG classification** | **MRI results** | **Biochemical signs of mitochondrial/ neurotransmitter abnormalities** | **Severity of the GDD/ID** | **Disease course** | **Reference** |
| *CACNA1A* | ID and ataxia | Telangiectasia, nystagmus and exophthalmos | 601011 | Unknown  p.Gly682Trp | Missense | Unknown | Deleterious | Normal | Not clear | ID | Not clear | ^7^ |
| *CACNA1A* | GDD and ataxia | Nystagmus | 601011 | Inherited (father)  p.Thr666Met | Missense | Unknown | Pathogenic | Cerebellar atrophy | Not done | Moderate GDD | Non progressive | ^8^ |
| *CACNA1A* | GDD | Bilateral esotropia | 601011 | De novo  p.Pro1353Leu | Missense | 95% reduction in peak current density | Pathogenic | Normal | Negative metabolic tests | GDD | Not clear | ^9^ |
| *CACNA1A* | EE | None | 601011 | Sister  p.Arg1351* | Nonsense | LOF | Pathogenic | Unknown | Not clear | GDD | Not clear | ^10^ |
| *CACNA1A* | EOEE | None | 601011 | Inherited  p.Arg279Cys | Missense | Unknown | Deleterious, | Cerebellar atrophy | Negative metabolic tests | GDD | Not clear | ^11^ |
| *CACNA1A* | GDD, early-onset cerebellar ataxia and dysarthria | Nystagmus and moderate conductive deafness | 601011 | Inherited  p.Arg279Cys | Missense | Unknown | Deleterious | Cerebellar atrophy | Negative metabolic tests | Mild ID | Not clear | ^11^ |
| *CACNA1A* | ID, early-onset cerebellar ataxia and dysarthria | Nystagmus | 601011 | Inherited  p.Arg279Cys22 | Missense | Unknown | Deleterious | Cerebellar atrophy | Negative metabolic tests | Mild ID | Not clear | ^11^ |
| *CACNA1A* | GDD and EOEE | Blind and mild dysmorphic features | 601011 | Inherited  p.Arg158Thrfs*6 | Nonsense | Truncation of the protein in the exon three | Pathogenic | Progressive cerebral, cerebellar, and optic nerve atrophy | Not clear | Profound GDD | Progressive | ^12^ |
| **Names of the gene** | **Syndrome/**  **phenotype** | **Other clinical features/ organs affected** | **OMIM numbers** | **Inheritance/Nucleotide or Protein change** | **Type of mutation** | **Altered protein function** | **Silico modeling prediction/ACMG classification** | **MRI results** | **Biochemical signs of mitochondrial/ neurotransmitter abnormalities** | **Severity of the GDD/ID** | **Disease course** | **Reference** |
| *CACNA1A* | GDD, EOEE, migraine, vertigo attacks | Blind and psychiatric symptoms | 601011 | Inherited  p.Ala158Thrfs*6 | Nonsense | Truncation of the protein in the exon three | Pathogenic | Progressive cerebral, cerebellar, and optic nerve atrophy | Not clear | Profound GDD | Progressive | ^12^ |
| *CACNA1A* | ID, EP and adult-onset ataxia | Myoclonus | 601011 | De novo  p.Ala713Thr | Missense | Unknown | Deleterious/Likely pathogenic | Unknown | Not clear | ID | Not clear | ^13^ |
| *CACNA1A* | GDD, progressive cerebellar ataxia, coma, and hemiplegia | Paroxysmal tonic upgaze | 601011 | Unknown  Arg1349Gln | Missense | Increased open probability of the channel | Disease causing | Cerebellar atrophy | Not clear | GDD | Progressive | ^14^ |
| *CACNA1A* | GDD, progressive cerebellar ataxia, coma, hemiplegia | Paroxysmal tonic upgaze | 601011 | Unknown  Arg1349Gln | Missense | Increased open probability of the channel | Disease causing | Cerebellar atrophy | Not clear | GDD | Progressive | ^14^ |
| *CACNA1A* | ID and migraine | Paroxysmal tonic upgaze | 601011 | Unknown  Arg1666His | Missense | Unknown | Disease causing | Normal | Not clear | ID | Progressive | ^14^ |
| *CACNA1A* | GDD, progressive cerebellar ataxia and ADHD | Dysmetric saccades and esotropic trabismus | 601011 | Unknown  Arg583Gln | Missense | Unknown | Disease causing | Normal | Not clear | GDD | Progressive | ^14^ |
| *CACNA1A* | GDD and cerebellar ataxia | Dysmetric saccades | 601011 | Unknown  Asp1337Tyr | Missense | Unknown | Disease causing | Cerebellar vermis  atrophy | Not clear | GDD | Progressive | ^14^ |
| **Names of the gene** | **Syndrome/**  **phenotype** | **Other clinical features/ organs affected** | **OMIM numbers** | **Inheritance/Nucleotide or Protein change** | **Type of mutation** | **Altered protein function** | **Silico modeling prediction/ACMG classification** | **MRI results** | **Biochemical signs of mitochondrial/ neurotransmitter abnormalities** | **Severity of the GDD/ID** | **Disease course** | **Reference** |
| *CACNA1A* | GDD, episodic ataxia and migraine | Paroxysmal tonic upgaze | 601011 | Unknown  p.Gly297Arg | Missense | Unknown | Disease causing | Normal | Not clear | GDD | Progressive | ^14^ |
| *CACNA1A* | GDD, episodic ataxia and migraine | Alternatin, non-accommodative esotropic strabismus | 601011 | Unknown  Ser218Leu | Missense | Unknown | Disease causing | Unknown | Not clear | GDD | Progressive | ^14^ |
| *CACNA1A* | ID, static cerebellar signs, hypotonia, and coordination difficulties | Benign paroxysmal tonic  upgaze | 601011 | Unknown  p.Gln681Argfs*Xaa17 | Nonsense | LOF | Pathogenic | Normal | Not clear | Moderate ID | Progressive | ^15^ |
| *CACNA1A* | ID and episodic ataxia | Benign paroxysmal tonic upgaze | 601011 | Unknown  p.Gln681Argfs*Xaa17 | Nonsense | LOF | Pathogenic | Cerebellar vermis atrophy | Not clear | Mild ID | Progressive | ^15^ |
| *CACNA1A* | ID and episodic ataxia | None | 601011 | Unknown  p.Thr1458Met | Missense | Unknown | Pathogenic | Cerebellar vermis atrophy | Not clear | Moderate ID | Progressive | ^15^ |
| *CACNA1A* | ID and episodic ataxia | Nystagmus | 601011 | Unknown  p.Gly677Arg | Missense | Unknown | Pathogenic | Cerebellar vermis atrophy | Not clear | Mild ID | Progressive | ^15^ |
| *CACNA1A* | ID, congenital ataxia, and dyskinesia | None | 601011 | De novo  Arg1350Gln | Missense | Unknown | Damaging | Cerebellar vermis atrophy | Normal | ID | Not clear | ^16^ |
| *CACNA1A* | ID and comatose episodes | Blindness and nystagmus | 601011 | Unknown  Ser218Leu | Missense | Unknown | Disease causing | Cerebellar atrophy | High pyruvate | ID | Not clear | ^17^ |
| *CACNA1A* | ID and comatose episodes | None | 601011 | Unknown  Ser218Leu | Missense | Unknown | Disease causing | Cerebellar atrophy | Not clear | ID | Progressive | ^18^ |
| **Names of the gene** | **Syndrome/**  **phenotype** | **Other clinical features/ organs affected** | **OMIM numbers** | **Inheritance/Nucleotide or Protein change** | **Type of mutation** | **Altered protein function** | **Silico modeling prediction/ACMG classification** | **MRI results** | **Biochemical signs of mitochondrial/ neurotransmitter abnormalities** | **Severity of the GDD/ID** | **Disease course** | **Reference** |
| *CACNA1A* | ID, comatose episodes and hemiplegia | Nystagmus | 601011 | Inherited  ThrT666Met | Missense | Unknown | Pathogenic | Unknown | Not clear | ID | Progressive | ^19^ |
| *CACNA1A* | ID, comatose episodes and hemiplegia | Nystagmus | 601011 | Unknown  p.Gly5361Thr | Missense | Unknown | Disease causing | Hemispheric edema | Not clear | ID | Progressive | ^20^ |
| *CACNA1A* | ID, comatose episodes and hemiplegia | Nystagmus | 601011 | Unknown  p.Gly5361Thr | Missense | Unknown | Disease causing | Normal | Not clear | ID | Progressive | ^20^ |
| *CACNA1A* | ID, EP, ataxia and nystagmus | Nystagmus | 601011 | Unknown  p.Arg1278* | Stop-gain mutation | LOF | Pathogenic | Hippocampal asymmetry | Not clear | Severe ID | Progressive | ^21^ |
| *CACNA1A* | ID, ataxia and nystagmus | Nystagmus | 601011 | Unknown  p.Arg957fs* | Frameshift mutation | LOF | Pathogenic | Unknown | Not clear | Mild ID | Progressive | ^21^ |
| *CACNA1A* | ID, EP, ataxia and ADHD | Nystagmus | 601011 | Unknown  p.Arg957fs* | Frameshift mutation | LOF | Pathogenic | Unknown | Not clear | Mild ID | Progressive | ^21^ |
| *CACNA1A* | ID, EP, ataxia and ADHD | Nystagmus | 601011 | Unknown  c.868+5Gly4Ala | Splice-site | LOF | Pathogenic | T2 Hyperintensity of left globus pallidus | Not clear | Mild ID | Progressive | ^21^ |
| *CACNA1A* | ID, ataxia and ADHD | Nystagmus | 601011 | Inherited  Del19p13.13 | Deletion | LOF | Pathogenic | Unknown | Not clear | Mild ID | Progressive | ^21^ |
| *CACNA1A* | ID, ataxia and ADHD | Nystagmus | 601011 | Inherited  Del19p13.13 | Deletion | LOF | Pathogenic | Normal | Not clear | Mild-moderate ID | Progressive | ^21^ |
| **Names of the gene** | **Syndrome/**  **phenotype** | **Other clinical features/ organs affected** | **OMIM numbers** | **Inheritance/Nucleotide or Protein change** | **Type of mutation** | **Altered protein function** | **Silico modeling prediction/ACMG classification** | **MRI results** | **Biochemical signs of mitochondrial/ neurotransmitter abnormalities** | **Severity of the GDD/ID** | **Disease course** | **Reference** |
| *CACNA1A* | ID and ataxia | None | 601011 | Unknown  c.959Gly>Ala | Missense | Unknown | Pathogenic | Unknown | Not clear | Low IQ | Progressive | ^22^ |
| *CACNA1A* | ID and ataxia | Schizophrenia | 601011 | Unknown  c.3102+2Thr>Cys | Missense | Unknown | Pathogenic | Unknown | Not clear | Low IQ | Progressive | ^22^ |
| *CACNA1A* | ID and ataxia | Anxiety disorder | 601011 | Unknown  c.3603dup | Duplication | Unknown | Pathogenic | Unknown | Not clear | Low IQ | Progressive | ^22^ |
| *CACNA1A* | ID and ataxia | Depression | 601011 | Unknown  Arg198Gln | Missense | Unknown | Pathogenic | Unknown | Not clear | Low IQ | Progressive | ^22^ |
| *CACNA1A* | ID and ataxia | None | 601011 | Unknown  Ser218Leu | Missense | Unknown | Pathogenic | Unknown | Not clear | Low IQ | Progressive | ^22^ |
| *CACNA1A* | ID and ataxia | None | 601011 | Unknown  Gly540Arg | Missense | Unknown | Pathogenic | Unknown | Not clear | Low IQ | Progressive | ^22^ |
| *CACNA1A* | ID and ataxia | None | 601011 | Unknown  Trp670Cys | Missense | Unknown | Pathogenic | Unknown | Not clear | Low IQ | Progressive | ^22^ |
| *CACNA1A* | ID and ataxia | None | 601011 | Unknown  Arg1668Trp | Missense | Unknown | Pathogenic | Unknown | Not clear | Low IQ | Progressive | ^22^ |
| **Names of the gene** | **Syndrome/**  **phenotype** | **Other clinical features/ organs affected** | **OMIM numbers** | **Inheritance/Nucleotide or Protein change** | **Type of mutation** | **Altered protein function** | **Silico modeling prediction/ACMG classification** | **MRI results** | **Biochemical signs of mitochondrial/ neurotransmitter abnormalities** | **Severity of the GDD/ID** | **Disease course** | **Reference** |
| *CACNA1A* | Epileptic encephalopathy and ID | None | 601011 | De novo  p.Gly230Val | Missense | Reduced whole-cell current densities and decreased channel expression at the cell membrane | Pathogenic | Normal | Not clear | Moderate ID | Progressive | ^23^ |
| *CACNA1A* | ID, ataxia and LGS | Tremors | 601011 | De novo  p.Ala713Thr | Missense | Increased whole-cell currents and facilitated current activation (hyperpolarized shift | Pathogenic | Normal | Not clear | Severe ID | Progressive | ^23^ |
| *CACNA1A* | ID, EE and ataxia | Nystagmus | 601011 | De novo  p.Ile1357Ser | Missense | Reduced whole-cell current densities and decreased channel expression at the cell membrane | Pathogenic | Cerebellar atrophy | Not clear | Severe ID | Progressive | ^23^ |
| *CACNA1A* | GDD, EE and ataxia | Optic nerve glioma and tremors | 601011 | De novo  p.Val1396Met and  p.Gly2314Ser | Missense | Increased whole-cell currents and facilitated current activation (hyperpolarized shift)) | Pathogenic | Normal | Not clear | Moderate GDD | Progressive | ^23^ |
| **Names of the gene** | **Syndrome/**  **phenotype** | **Other clinical features/ organs affected** | **OMIM numbers** | **Inheritance/Nucleotide or Protein change** | **Type of mutation** | **Altered protein function** | **Silico modeling prediction/ACMG classification** | **MRI results** | **Biochemical signs of mitochondrial/ neurotransmitter abnormalities** | **Severity of the GDD/ID** | **Disease course** | **Reference** |
| *CACNA1A* | ID, migraine and ataxia | Nystagmus | 601011 | Inherited  c.1063dupGly | Frameshift and a premature stop codon | Unknown | Disease causing | Unknown | Not clear | Severe ID | Progressive | ^24^ |
| *CACNA1A* | GDD and ataxia | Abnormal behaviors, hyperreflexia and eye movement disorder | 601011 | De novo  p.Arg1664Gln | Missense | LOF | Pathogenic | Normal | Not clear | GDD | Not clear | ^25^ |
| *CACNA1A* | GDD and ataxia | Esotropia,  hyperopia, a tented upper lip and prominent jaw | 601011 | De novo  p.Arg1673Pro | Missense | GOF but later it was revealed to be LOF | Pathogenic | Cerebellar atrophy, thinning of the body and splenium of the corpus callosum; mild delay in deep white matter myelination | Not clear | GDD | Not clear | ^25,26^ |
| *CACNA1A* | GDD and ataxia | Difficulty sleeping, sensory processing disorder, strabismus, myopia, and astigmatism | 601011 | De novo  p.Arg1664Gln | Missense | LOF | Pathogenic | Thin corpus callosum | Not clear | GDD | Not clear | ^25^ |
| **Names of the gene** | **Syndrome/**  **phenotype** | **Other clinical features/ organs affected** | **OMIM numbers** | **Inheritance/Nucleotide or Protein change** | **Type of mutation** | **Altered protein function** | **Silico modeling prediction/ACMG classification** | **MRI results** | **Biochemical signs of mitochondrial/ neurotransmitter abnormalities** | **Severity of the GDD/ID** | **Disease course** | **Reference** |
| *CACNA1A* | GDD and ataxia | Attention deficit disorder/executive dysfunction, hyporeflexic, alternating strabismus and esotropia | 601011 | De novo  p.Arg1664Gln | Missense | LOF | Pathogenic | Atrophy of cerebellar vermis | Not clear | GDD | Not clear | ^25^ |
| *CACNA1A* | GDD and ataxia | Aggression and ocular apraxia | 601011 | De novo  p.Arg1664Gln | Missense | LOF | Pathogenic | Atrophy of  cerebellar vermis | Not clear | GDD | Not clear | ^25^ |
|  |  |  |  |  |  |  |  |  |  |  |  |  |
| *CACNA1A* | GDD, EP, and ASD | None | 601011 | p.Pro2312_Q2313ins | Insertion | Unknown | Unknown | Partial agenesis of the corpus callosum | Not clear | GDD | Not clear | ^27^ |
| *CACNA1A* | Atypical Rett Syndrome | Tremors and ataxia | 601011 | De novo  p.Ala710Thr | Missense | Unknown | Likely pathogenic | Mild hypo-myelination | Normal | ID | Progressive | ^28^ |
| *CACNA1A* | ID, EP and ataxia | None | 601011 | De novo  p.Ser1799Leu | Missense | Unknown | Pathogenic | Cerebellar  atrophy | Not clear | ID | Not clear | ^29^ |
| *CACNA1A* | ID, episodic ataxia, and ADHD | Strabismus | 601011 | Inherited  p.Gln1154* | Nonsense | Unknown | Deleterious | Normal | None | Mild to moderate ID | Progressive | ^30^ |
| **Names of the gene** | **Syndrome/**  **phenotype** | **Other clinical features/ organs affected** | **OMIM numbers** | **Inheritance/Nucleotide or Protein change** | **Type of mutation** | **Altered protein function** | **Silico modeling prediction/ACMG classification** | **MRI results** | **Biochemical signs of mitochondrial/ neurotransmitter abnormalities** | **Severity of the GDD/ID** | **Disease course** | **Reference** |
|  |  |  |  |  |  |  |  |  |  |  |  |  |
| *CACNA1A* | EIMFS | Strabismus, nystagmus, ataxia, tremor, and athetosis. | 601011 | De novo  p.Glu101Gln | Missense | Unknown | Pathogenic | Normal | None | Severe ID | Non progressive | ^31^ |
| *CACNA1A* | EOEE | Strabismus, nystagmus, ataxia, tremor, and athetosis. | 601011 | Unknown  p.Ser218Leu | Missense | Unknown | VOUS | Hypoxic lesion | None | Severe ID | Non progressive | ^31^ |
| *CACNA1A* | EOEE | Strabismus, nystagmus, ataxia, tremor, and athetosis. | 601011 | De novo  p.Ala713Thr | Missense | Unknown | Pathogenic | Bimesial temporal lobe increased T2 signal | None | Severe ID | Non progressive | ^31^ |
| *CACNA1A* | EOEE | Strabismus, nystagmus, ataxia, tremor, and athetosis. | 601011 | Mosaic mother  p.Ala713Thr | Missense | Unknown | Pathogenic | Normal | None | Severe ID | Non progressive | ^31^ |
| *CACNA1A* | EOEE | Strabismus, nystagmus, ataxia, tremor, and athetosis. | 601011 | De novo  p. Ala1511Ser | Missense | Unknown | Pathogenic | Normal | None | Moderate ID | Non progressive | ^31^ |
| *CACNA1A* | ID and ataxia | None | 601011 | De novo  c.4503-4505delCTT | Non sense | Unknown | Deleterious | Cerebellar atrophy | None | Severe ID | Progressive | ^32^ |
| **Names of the gene** | **Syndrome/**  **phenotype** | **Other clinical features/ organs affected** | **OMIM numbers** | **Inheritance/Nucleotide or Protein change** | **Type of mutation** | **Altered protein function** | **Silico modeling prediction/ACMG classification** | **MRI results** | **Biochemical signs of mitochondrial/ neurotransmitter abnormalities** | **Severity of the GDD/ID** | **Disease course** | **Reference** |
| *CACNA1C* | TS | Atrioventricular block, patent ductus arteriosus, syndactyly, hypoglycemia and facial abnormalities | 114205 | De novo  Gly402Ser | Missense | Complete loss of voltage-dependent channel inactivation | Pathogenic | Unknown | Not clear | GDD | Not clear | ^33,34^ |
| *CACNA1C* | TS | Atrioventricular block, syndactyly, hypoglycemia and facial abnormalities | 114205 | De novo  Gly406Ser | Missense | Complete loss of voltage-dependent channel inactivation | Pathogenic | Unknown | Not clear | GDD | Not clear | ^33,34^ |
| *CACNA1C* | TS | Syndactyly | 114205 | De novo  Ser405Arg | Missense | Unknown | Pathogenic | Unknown | Not clear | GDD | Not clear | ^33^ |
| *CACNA1C* | TS | Syndactyl, facial abnormalities and suspected immune disorder | 114205 | De novo  Gly406Ser | Missense | Complete loss of voltage-dependent channel inactivation | Pathogenic | Unknown | Not clear | GDD | Not clear | ^33,34^ |
| *CACNA1C* | TS | Atrioventricular block, ventral septal defect, syndactyly, hypoglycemia and facial abnormalities | 114205 | De novo  p.Gly406Ser | Missense | Complete loss of voltage-dependent channel inactivation | Pathogenic | Unknown | Not clear | GDD | Not clear | ^33,34^ |
| **Names of the gene** | **Syndrome/**  **phenotype** | **Other clinical features/ organs affected** | **OMIM numbers** | **Inheritance/Nucleotide or Protein change** | **Type of mutation** | **Altered protein function** | **Silico modeling prediction/ACMG classification** | **MRI results** | **Biochemical signs of mitochondrial/ neurotransmitter abnormalities** | **Severity of the GDD/ID** | **Disease course** | **Reference** |
| *CACNA1C* | TS | Atrioventricular block, hypoglycemia and facial abnormalities | 114205 | Inherited (father)  p.Gly406Arg | Missense | Complete loss of voltage-dependent channel inactivation | Pathogenic | Unknown | Not clear | GDD | Not clear | ^33,34^ |
| *CACNA1C* | TS | Syndactyly | 114205 | Unknown  Gly402Ser | Missense | Unknown | Pathogenic | Unknown | Not clear | GDD | Not clear | ^33^ |
| *CACNA1C* | TS | None | 114205 | De novo  Gly402Arg | Missense | Unknown | Pathogenic | Unknown | Not clear | GDD | Not clear | ^33^ |
| *CACNA1C* | TS | Atrioventricular block, patent ductus arteriosus, syndactyly, hypoglycemia and facial abnormalities | 114205 | Inherited (mother)  p.Gly406Arg | Missense | Complete loss of voltage-dependent channel inactivation | Pathogenic | Unknown | Not clear | GDD | Not clear | ^33,34^ |
| *CACNA1C* | TS | Syndactyly and facial abnormalities | 114205 | De novo  p.Gly406Arg | Missense | Complete loss of voltage-dependent channel inactivation | Pathogenic | Unknown | Not clear | GDD | Not clear | ^33,34^ |
| *CACNA1C* | TS | Syndactyly, hypoglycemia and facial abnormalities | 114205 | De novo  Cys1021Arg | Missense | Unknown | Pathogenic | Unknown | Not clear | GDD | Not clear | ^33^ |
| **Names of the gene** | **Syndrome/**  **phenotype** | **Other clinical features/ organs affected** | **OMIM numbers** | **Inheritance/Nucleotide or Protein change** | **Type of mutation** | **Altered protein function** | **Silico modeling prediction/ACMG classification** | **MRI results** | **Biochemical signs of mitochondrial/ neurotransmitter abnormalities** | **Severity of the GDD/ID** | **Disease course** | **Reference** |
| *CACNA1C* | TS | Atrioventricular block, dysmorphic facial features, and syndactyly | 114205 | De novo  p.Gly406Arg | Missense | Complete loss of voltage-dependent channel inactivation | Pathogenic | Unknown | Not clear | GDD | Not clear | ^35^ |
| *CACNA1C* | TS and infantile spasms | Atrioventricular block, severe asymmetric septal hypertrophy, left atrial enlargement., dysmorphic facial features, and syndactyly | 114205 | De novo  p.Ile1166Thr | Missense | Loss of current density and a gain-of-function shift in activation, leading to an increased window current | Pathogenic | Progressive cerebellar and cerebral atrophy | Not clear | GDD | Progressive | ^36^ |
| *CACNA1C* | TS | Lethal arrhythmia, webbing of fingers and toes, congenital heart disease, immune deficiency and intermittent hypoglycemia | 114205 | De novo  p.Gly406Arg | Missense | Complete loss of voltage-dependent  inactivation | Pathogenic | Unknown | Not clear | GDD | Progressive | ^37^ |
| *CACNA1C* | TS 2 (GDD without autism) | Dysmorphic features, torsade de pointes and hypoglycemia | 114205 | Unknown  p.Gly406Arg | Missense | Delays of calcium channel  inactivation | Pathogenic | Normal | Not clear | GDD | Non progressive | ^38^ |
| **Names of the gene** | **Syndrome/**  **phenotype** | **Other clinical features/ organs affected** | **OMIM numbers** | **Inheritance/Nucleotide or Protein change** | **Type of mutation** | **Altered protein function** | **Silico modeling prediction/ACMG classification** | **MRI results** | **Biochemical signs of mitochondrial/ neurotransmitter abnormalities** | **Severity of the GDD/ID** | **Disease course** | **Reference** |
| *CACNA1C* | GDD and EP | Syndactyly a, pulmonary hypertension, postnatal growth failure, joint contractures and multiple congenital anomalies | 114205 | De novo  p.Arg1024Gly | Missense | Unknown | Pathogenic | Enlarged perivascular space and spikes at right parietal region | Metabolic tests were negative | GDD | Non progressive | ^39^ |
| *CACNA1C* | GDD, attention deficit and late-onset EP | Congenital cardiac anomalies and dysmorphic features | 114205 | Inherited (father)  3717+1_3717 +2insA | Insertion | Unknown | Disease causative | Unknown | Not clear | GDD | Progressive | ^40^ |
| *CACNA1C* | GDD and EP | Hypotonia, dysmorphic features, laryngomalacia, syndactyly, camptodactyly, and anal stenosis | 114205 | De novo  p.Val1363Met | Missense | Unknown | Likely pathogenic | Unknown | Not clear | Mild GDD | Non progressive | ^40^ |
| **Names of the gene** | **Syndrome/**  **phenotype** | **Other clinical features/ organs affected** | **OMIM numbers** | **Inheritance/Nucleotide or Protein change** | **Type of mutation** | **Altered protein function** | **Silico modeling prediction/ACMG classification** | **MRI results** | **Biochemical signs of mitochondrial/ neurotransmitter abnormalities** | **Severity of the GDD/ID** | **Disease course** | **Reference** |
| *CACNA1C* | GDD and EP | Prolonged QTC interval, arrhythmias, microcephaly, short stature, lower extremity weakness and atrophy with hyperreflexia, spastic diplegia, multiple dental caries and episodes of rhabdomyolysis | 114205 | Unknown  p.Gly1911Arg | Missense | GOF | Pathogenic | Unknown | Decreased mitochondrial complex I and III activity by enzymology assay and quantitative immunoblot. | GDD | Non progressive | ^41^ |
| *CACNA2D2* | GDD and EP | Axial hypotonia and choreiform movements | 607082 | p.Leu1040Pro | Missense | Dysfunction of alpha2delta2, resulting in reduced current density and slow inactivation in neuronal calcium channels | Pathogenic | Cerebellar atrophy | Not clear | Severe GDD | Progressive | ^42^ |
| **Names of the gene** | **Syndrome/**  **phenotype** | **Other clinical features/ organs affected** | **OMIM numbers** | **Inheritance/Nucleotide or Protein change** | **Type of mutation** | **Altered protein function** | **Silico modeling prediction/ACMG classification** | **MRI results** | **Biochemical signs of mitochondrial/ neurotransmitter abnormalities** | **Severity of the GDD/ID** | **Disease course** | **Reference** |
| *CACNA2D2* | GDD and EP | Axial hypotonia and choreiform movements | 607082 | p.Leu1040Pro | Missense | Dysfunction of alpha2delta2resulting in reduced current density and slow inactivation in neuronal calcium channels | Pathogenic | Cerebellar atrophy | Not clear | Severe GDD | Progressive | ^42^ |
| *CACNA2D2* | GDD and EP | Axial hypotonia and choreiform movements | 607082 | p.Leu1040Pro | Missense | Dysfunction of alpha2delta2resulting in reduced current density and slow inactivation in neuronal calcium channels | Pathogenic | Cerebellar atrophy | Not clear | Severe GDD | Progressive | ^42^ |
| *CACNA2D2* | EOEE | Hypotonia and abnormal eye movements, tremor and ataxia | 607082 | Inherited  p.Pro261Leu and  p.Leu1046Pro | Missense | Unknown | Deleterious/Disease causative | Cerebellar atrophy | Negative | GDD | Progressive | ^43^ |
| **Names of the gene** | **Syndrome/**  **phenotype** | **Other clinical features/ organs affected** | **OMIM numbers** | **Inheritance/Nucleotide or Protein change** | **Type of mutation** | **Altered protein function** | **Silico modeling prediction/ACMG classification** | **MRI results** | **Biochemical signs of mitochondrial/ neurotransmitter abnormalities** | **Severity of the GDD/ID** | **Disease course** | **Reference** |
| *CACNA2D2* | ID and EP | Dyskinesia, dysmorphic features, oculo-motor apraxia, strabismus, nystagmus, axial and leg hypertonia, head tonic extension, erratic limb movements, tremor, brisk symmetric reflexes, hyperglycaemia and glycosuria | 607082 | Inherited  p.Asn432Thrfs∗35 | Nonsense | LOF | Pathogenic | Cerebellar atrophy | Negative | Severe GDD | Progressive | ^44^ |
| *CACNA2D2* | EOEE and ataxia | Restlessness, sleep disturbance and dysmorphic features | 607082 | Inherited  p.Arg593Pro | Missense | Unknown | Likely damaging | Cerebellar atrophy and mild cerebral atrophy | Not clear | GDD | Progressive | ^45^ |
| *CACNA2D2* | EOEE | None | 607082 | Inherited  p.Tyr162* | Nonsense | LOF | Pathogenic | Global  cerebral and cerebellar atrophy | Not clear | GDD | Progressive | ^45^ |
| **Names of the gene** | **Syndrome/**  **phenotype** | **Other clinical features/ organs affected** | **OMIM numbers** | **Inheritance/Nucleotide or Protein change** | **Type of mutation** | **Altered protein function** | **Silico modeling prediction/ACMG classification** | **MRI results** | **Biochemical signs of mitochondrial/ neurotransmitter abnormalities** | **Severity of the GDD/ID** | **Disease course** | **Reference** |
| *CACNA2D1* | EP and ID | None | 114204 | De novo  46,X,t(X;7)(p10;q21.2) | Nonsense | Haploinsufficiency |  | Bilateral frontotemporal polymicrogyria, mega cisterna  magna and a cyst in the cavum veli interpositi, under the splenium of the corpus callosum expanding posterior to the supravermian cistern. | Not clear | Mild ID | Not clear | ^46^ |
| *CACNA2D1* | EP and ID | Facial dysmorphism, clinodactyly V, brachymetacarpy and -tarsy V 1 | 114204 | De novo  7.5Mb deletion chr7.hg19:g.  (79,314,673_79,337,679)_(86,790 harboring 14 genes. | Deletion | Haploinsufficiency | Disease causing | Global cortical atrophy. | Not clear | Mild ID | Not clear | ^46^ |
| **Names of the gene** | **Syndrome/**  **phenotype** | **Other clinical features/ organs affected** | **OMIM numbers** | **Inheritance/Nucleotide or Protein change** | **Type of mutation** | **Altered protein function** | **Silico modeling prediction/ACMG classification** | **MRI results** | **Biochemical signs of mitochondrial/ neurotransmitter abnormalities** | **Severity of the GDD/ID** | **Disease course** | **Reference** |
| *CACNA2D1* | EP, ID and ataxia | Dysmorphic features, abnormal skin, short stature, transient diabetes with hyperinsulinemia | 14204 | Inherited (mother) 2.72Mb deletion Chr7: 81,217,070-83,933,784) containing 5 genes | Deletion | Haploinsufficiency | Disease causing | Normal | Not clear | Severe ID | Not clear | ^46^ |
| *CACNA2D1* | ID, EP and autistic behavior | None | 114204 | De novo  3.3 Mb deletion at 7q21.11 harboring 7 genes. | Deletion | Haploinsufficiency | Damaging | Normal | Not clear | ID | Progressive | ^47^ |
| *CACNA1D* | GDD, EP, autistic behavior and hyperactivity | Moderate hearing impairment | 114204 | Unknown  p.Gln567His | Missense | Reduction in Cav1.3 current density and suppresses voltage-dependent inactivation of the channel | Disease causing | Normal | Not clear | Mild ID | Not clear | ^48^ |
| *CACNA1D* | GDD, EP, and ASD | Aggressiveness, motoric agitation  and stereotypic hand movements | 114204 | De novo  p.Val401Leu | Missense | Reduction in Cav1.3 current density and suppresses voltage-dependent inactivation of the channel | Disease causing | Normal | Not clear | Profound GDD | Not clear | ^49^ |
| **Names of the gene** | **Syndrome/**  **phenotype** | **Other clinical features/ organs affected** | **OMIM numbers** | **Inheritance/Nucleotide or Protein change** | **Type of mutation** | **Altered protein function** | **Silico modeling prediction/ACMG classification** | **MRI results** | **Biochemical signs of mitochondrial/ neurotransmitter abnormalities** | **Severity of the GDD/ID** | **Disease course** | **Reference** |
| *CACNA1D* | ID and ASD | Primary aldosteronism | 114204 | De novo  p.Ala749Gly | Missense | GOF | Disease causing | Unknown | Not clear | ID | Not clear | ^50^ |
| *CACNA1D* | ID and ASD | Primary aldosteronism | 114204 | De novo  Gly407Arg | Missense | GOF | Disease causing | Unknown | Not clear | ID | Not clear | ^50^ |
| *CACNA1D* | GDD | Hyperinsulinaemic hypoglycaemia, heart defects, and severe  hypotonia | 114204 | De novo  p.Gly403Asp | Missense | Increased current densities and a hyperpolarizing shift of voltage-dependence of activation and inactivation | Disease causing | Unknown | Not clear | GDD | Not clear | ^51^ |
| *CACNA1D* | GDD, ASD, EP and ADHD | None | 114204 | De novo  p. Ser652Leu | Missense | GOF | Disease causing | Unknown | Not clear | Severe GDD | **Not clear** | ^52^ |
| *CACNA1D* | GDD and ASD | None | 114204 | De novo  p. Ser652Leu | Missense | GOF | Disease causing | Unknown | Not clear | Severe GDD | **Not clear** | ^52^ |
| *CACNA1E* | DEE | Spastic quadriplegia, hypotonia | 601013 | De novo  p.Leu228Pro | Missense | Unknown | Disease causing | White matter  volume loss | Not clear | Profound GDD | Not clear | ^53^ |
| *CACNA1E* | DEE | Severe truncal hypotonia, appendicular hypertonia and dystonia | 601013 | De novo  p.Gly348Arg | Missense | Unknown | Disease causing | Normal | Not clear | Profound GDD | Not clear | ^53^ |
| **Names of the gene** | **Syndrome/**  **phenotype** | **Other clinical features/ organs affected** | **OMIM numbers** | **Inheritance/Nucleotide or Protein change** | **Type of mutation** | **Altered protein function** | **Silico modeling prediction/ACMG classification** | **MRI results** | **Biochemical signs of mitochondrial/ neurotransmitter abnormalities** | **Severity of the GDD/ID** | **Disease course** | **Reference** |
| *CACNA1E* | DEE | Severe axial hypotonia, appendicular hypertonia and dystonia | 601013 | De novo  p.Gly352Arg | Missense | Unknown | Disease causing | Cortical atrophy | Not clear | Profound GDD | Not clear | ^53^ |
| *CACNA1E* | DEE | Spastic quadriplegia, hypotonia, congenital contractures, macrocephaly and dystonia | 601013 | De novo  p.Gly352Arg | Missense | Unknown | Disease causing | Normal | Not clear | Profound GDD | Not clear | ^53^ |
| *CACNA1E* | DEE | Spastic dystonic quadriplegia, hypotonia, macrocephaly and dystonia | 601013 | De novo  p.Gly352Arg | Missense | Unknown | Disease causing | Normal | Not clear | Profound GDD | Not clear | ^53^ |
| *CACNA1E* | DEE | Hypotonia, appendicular, hypertonia, macrocephaly and dystonia | 601013 | De novo  p.Gly352Arg | Missense | Unknown | Disease causing | Normal | Not clear | Profound GDD | Not clear | ^53^ |
| *CACNA1E* | DEE | Spastic dystonic quadriplegia and dystonia | 601013 | De novo  p.Gly352Arg | Missense | Unknown | Disease causing | Cortical atrophy | Not clear | Profound GDD | Not clear | ^53^ |
| *CACNA1E* | DEE | Hypotonia, dystonia and congenital contractures | 601013 | De novo  p.Gly352Arg | Missense | Unknown | Disease causing | White matter volume loss | Not clear | Profound GDD | Not clear | ^53^ |
| **Names of the gene** | **Syndrome/**  **phenotype** | **Other clinical features/ organs affected** | **OMIM numbers** | **Inheritance/Nucleotide or Protein change** | **Type of mutation** | **Altered protein function** | **Silico modeling prediction/ACMG classification** | **MRI results** | **Biochemical signs of mitochondrial/ neurotransmitter abnormalities** | **Severity of the GDD/ID** | **Disease course** | **Reference** |
| *CACNA1E* | DEE | Severe hypotonia and  hyperreflexia | 601013 | De novo  p.Ile603Leu | Missense | Facilitated activation and increased current density | Disease causing | Normal | Not clear | Profound GDD | Not clear | ^53^ |
|  |  |  |  |  |  |  |  |  |  |  |  |  |
| *CACNA1E* | DEE | Severe axial hypotonia, neck dystonia, chorea, macrocephaly and congenital contractures | 601013 | De novo  p.Gly352Arg | Missense | Unknown | Disease causing | Hyperintense T2 signal in parietooccipital region | Not clear | Profound GDD | Not clear | ^53^ |
| *CACNA1E* | DEE | Severe axial hypotonia, appendicular hypertonia, dystonia, dyskinesia, myoclonus and congenital contractures | 601013 | De novo  p.Gly352Arg | Missense | Unknown | Disease causing | Normal | Not clear | Profound GDD | Not clear | ^53^ |
| *CACNA1E* | DEE | Severe axial hypotonia, appendicular hypertonia, dystonia, congenital contractures and macrocephaly | 601013 | De novo  p.Gly352Arg | Missense | Unknown | Disease causing | Normal | Not clear | Profound GDD | Not clear | ^53^ |
| **Names of the gene** | **Syndrome/**  **phenotype** | **Other clinical features/ organs affected** | **OMIM numbers** | **Inheritance/Nucleotide or Protein change** | **Type of mutation** | **Altered protein function** | **Silico modeling prediction/ACMG classification** | **MRI results** | **Biochemical signs of mitochondrial/ neurotransmitter abnormalities** | **Severity of the GDD/ID** | **Disease course** | **Reference** |
| *CACNA1E* | DEE | Severe diffuse hypotonia. dystonia and hyperkinetic | 601013 | De novo  p.Gly690Asp | Missense | Unknown | Disease causing | Normal | Not clear | Profound GDD | Not clear | ^53^ |
| *CACNA1E* | DEE | Profound hypotonia and congenital contractures | 601013 | De novo  p.Phe698Ser | Missense | Facilitated voltage-dependent activation and slowed inactivation | Disease causing | Normal | Not clear | Profound GDD | Not clear | ^53^ |
| *CACNA1E* | DEE | Severe diffuse hypotonia | 601013 | De novo  p.Ala700Thr | Missense | Facilitated voltage-dependent activation and slowed inactivation | Disease causing | Normal | Not clear | Profound GDD | Not clear | ^53^ |
| *CACNA1E* | DEE | Hypotonia, appendicular hypertonia, dystonia and chorea | 601013 | De novo  p.Ile701Val | Missense | Facilitated voltage-dependent activation and slowed inactivation | Disease causing | Normal | Not clear | Profound GDD | Not clear | ^53^ |
| *CACNA1E* | DEE | Hypotonia and macrocephaly | 601013 | De novo  p.Ile701Val | Missense | Facilitated voltage-dependent activation and slowed inactivation | Disease causing | Cortical atrophy | Not clear | Profound GDD | Not clear | ^53^ |
| **Names of the gene** | **Syndrome/**  **phenotype** | **Other clinical features/ organs affected** | **OMIM numbers** | **Inheritance/Nucleotide or Protein change** | **Type of mutation** | **Altered protein function** | **Silico modeling prediction/ACMG classification** | **MRI results** | **Biochemical signs of mitochondrial/ neurotransmitter abnormalities** | **Severity of the GDD/ID** | **Disease course** | **Reference** |
| *CACNA1E* | DEE | Severe axial hypotonia, appendicular hypertonia, chorea and macrocephaly | 601013 | De novo  p.Ile701Val | Missense | Facilitated voltage-dependent activation and slowed inactivation | Disease causing | Normal | Not clear | Profound GDD | Not clear | ^53^ |
| *CACNA1E* | DEE | Severe axial hypotonia, appendicular hypertonia and macrocephaly | 601013 | De novo  p.Ala702Thr | Missense | Facilitated voltage-dependent activation and slowed inactivation | Disease causing | Normal | Not clear | Profound GDD | Not clear | ^53^ |
| *CACNA1E* | DEE | Severe axial hypotonia, appendicular hypertonia, congenital contractures and macrocephaly | 601013 | De novo  p.Ala702Thr | Missense | Facilitated voltage-dependent activation and slowed inactivation | Disease causing | Normal | Not clear | Profound GDD | Not clear | ^53^ |
| *CACNA1E* | DEE | Severe hypotonia, congenital contractures and macrocephaly | 601013 | De novo  p.Ala702Thr | Missense | Facilitated voltage-dependent activation and slowed inactivation | Disease causing | Cortical atrophy | Not clear | Profound GDD | Not clear | ^53^ |
| *CACNA1E* | DEE | Profound hypotonia, spastic quadriplegia and congenital contractures | 601013 | De novo  p.Ala702Thr | Missense | Facilitated voltage-dependent activation and slowed inactivation | Disease causing | Hyperintensity in basal ganglia | Not clear | Profound GDD | Not clear | ^53^ |
| **Names of the gene** | **Syndrome/**  **phenotype** | **Other clinical features/ organs affected** | **OMIM numbers** | **Inheritance/Nucleotide or Protein change** | **Type of mutation** | **Altered protein function** | **Silico modeling prediction/ACMG classification** | **MRI results** | **Biochemical signs of mitochondrial/ neurotransmitter abnormalities** | **Severity of the GDD/ID** | **Disease course** | **Reference** |
| *CACNA1E* | DEE | Diffuse hypotonia, congenital contractures and macrocephaly | 601013 | De novo  p.Ala702Thr | Missense | Facilitated voltage-dependent activation and slowed inactivation | Disease causing | Normal | Not clear | Profound GDD | Not clear | ^53^ |
| *CACNA1E* | DEE | Severe axial hypotonia, congenital contractures and macrocephaly | 601013 | De novo  p.Ala702Thr | Missense | Facilitated voltage-dependent activation and slowed inactivation | Disease causing | Unknown | Not clear | Profound GDD | Not clear | ^53^ |
| *CACNA1E* | DEE | Hyptonia, spastic  quadriplegia | 601013 | De novo  p.Ile1422Phe | Missense | Unknown | Disease causing | Normal | Not clear | Profound GDD | Not clear | ^53^ |
| *CACNA1E* | DEE | Axial hypotonia | 601013 | De novo  p.Thr1425Asn | Missense | Unknown | Disease causing | Normal | Not clear | Severe GDD | Not clear | ^53^ |
| *CACNA1E* | DEE | Mild hypotonia | 601013 | De novo  p.Thr1425Asn | Missense | Unknown | Disease causing | Normal | Not clear | Severe GDD | Not clear | ^53^ |
| *CACNA1E* | DEE | Hypotonia | 601013 | De novo  p.Gly1430Arg | Missense | Unknown | Disease causing | Delayed myelination | Not clear | Severe GDD | Not clear | ^53^ |
| *CACNA1E* | DEE | Profound appendicular  hypotonia | 601013 | De novo  p.Ala1720Gly | Missense | Unknown | Disease causing | Thin corpus callosum | Not clear | Profound GDD | Not clear | ^53^ |
| **Names of the gene** | **Syndrome/**  **phenotype** | **Other clinical features/ organs affected** | **OMIM numbers** | **Inheritance/Nucleotide or Protein change** | **Type of mutation** | **Altered protein function** | **Silico modeling prediction/ACMG classification** | **MRI results** | **Biochemical signs of mitochondrial/ neurotransmitter abnormalities** | **Severity of the GDD/ID** | **Disease course** | **Reference** |
| *CACNA1G* | GDD, EP, ataxia, and motor impairment | Hypotonia, oculomotor apraxia, strabismus and multiple congenital anomalies | 604065 | De novo  p.Ala961Thr | Missense | Impaired channel inactivation properties with significantly slower kinetics and negatively shifted potential for half-inactivation. | Damaging | Cerebellar atrophy | Not clear | Severe ID | Progressive | ^54^ |
| *CACNA1G* | GDD, EP, ataxia and motor impairment | Hypotonia, oculomotor apraxia, strabismus and multiple congenital anomalies | 604065 | De novo  p.Ala961Thr | Missense | Impaired channel inactivation properties with significantly slower kinetics and negatively shifted potential for half-inactivation. | Damaging | Cerebellar atrophy | Not clear | Profound ID | Progressive | ^54^ |
| **Names of the gene** | **Syndrome/**  **phenotype** | **Other clinical features/ organs affected** | **OMIM numbers** | **Inheritance/Nucleotide or Protein change** | **Type of mutation** | **Altered protein function** | **Silico modeling prediction/ACMG classification** | **MRI results** | **Biochemical signs of mitochondrial/ neurotransmitter abnormalities** | **Severity of the GDD/ID** | **Disease course** | **Reference** |
| *CACNA1G* | GDD, EP, ataxia and motor impairment | Hyperopia | 604065 | De novo  p.Ala961Thr | Missense | Impaired channel inactivation properties with significantly slower kinetics and negatively shifted potential for half-inactivation. | Damaging | Cerebellar atrophy | Not clear | Severe ID | Progressive | ^54^ |
| *CACNA1G* | GDD, EP, ataxia and motor impairment | Hypotonia, oculomotor apraxia, strabismus and multiple congenital anomalies | 604065 | De novo  p.Met1531Val | Missense | Impaired channel inactivation properties with significantly slower kinetics and negatively shifted potential for half-inactivation. | Damaging | Cerebellar atrophy | Not clear | Profound ID | Progressive | ^54^ |
| **Names of the gene** | **Syndrome/**  **phenotype** | **Other clinical features/ organs affected** | **OMIM numbers** | **Inheritance/Nucleotide or Protein change** | **Type of mutation** | **Altered protein function** | **Silico modeling prediction/ACMG classification** | **MRI results** | **Biochemical signs of mitochondrial/ neurotransmitter abnormalities** | **Severity of the GDD/ID** | **Disease course** | **Reference** |
| *CACNA1G* | ID and ataxia | Spastic paaraplegia dysmorphic features, abnormal behavior, nystagmus, esotropia, Doll’s eyes, slightly pale papillae, atrial septal defect and other multiple congenital anomalies | 604065 | De novo  p.Ala961Thr | Missense | Impaired channel inactivation properties with significantly slower kinetics and negatively shifted potential for half-inactivation. | Disease causing | Cerebellar atrophy | Negative | Severe ID | Progressive | ^55^ |
| *CACNA1G* | ID and ataxia | Dysmorphic features, abnormal behavior, exotropia and other multiple congenital anomalies | 604065 | De novo  p.Ala961Thr | Missense | Impaired channel inactivation properties with significantly slower kinetics and negatively shifted potential for half-inactivation. | Disease causing | Cerebellar atrophy | Negative | Severe ID | Progressive | ^55^ |
| **Names of the gene** | **Syndrome/**  **phenotype** | **Other clinical features/ organs affected** | **OMIM numbers** | **Inheritance/Nucleotide or Protein change** | **Type of mutation** | **Altered protein function** | **Silico modeling prediction/ACMG classification** | **MRI results** | **Biochemical signs of mitochondrial/ neurotransmitter abnormalities** | **Severity of the GDD/ID** | **Disease course** | **Reference** |
| *CACNA1G* | ID and ataxia | Dysmorphic features, abnormal behavior, strabismus and other multiple congenital anomalies | 604065 | De novo  p.Ala961Thr | Missense | Impaired channel inactivation properties with significantly slower kinetics and negatively shifted potential for half-inactivation. | Disease causing | Cerebellar atrophy | Negative | Severe ID | Progressive | ^55^ |
| *CACNA1G* | ID and ataxia | Dysmorphic features, abnormal behavior, strabismus and other multiple congenital anomalies | 604065 | De novo  p.Met1531Val | Missense | Impaired channel inactivation properties with significantly slower kinetics and negatively shifted potential for half-inactivation. | Disease causing | Cerebellar atrophy | Negative | Severe ID | Progressive | ^55^ |
| *CACNA1F* | ID and EP | Spastic quadriplegia and cortical blindness | 300110 | Inherited  p.Ile745Thr | Missense | Unknown | Disease causing | Unknown | Not clear | Profound ID | Not clear | ^56^ |
| *CACNA1F* | ID and ASD | Lebers congenital amaurosis | 300110 | Inherited  p.Ile745Thr | Missense | Unknown | Disease causing | Unknown | Not clear | Profound ID | Not clear | ^56^ |
| **Names of the gene** | **Syndrome/**  **phenotype** | **Other clinical features/ organs affected** | **OMIM numbers** | **Inheritance/Nucleotide or Protein change** | **Type of mutation** | **Altered protein function** | **Silico modeling prediction/ACMG classification** | **MRI results** | **Biochemical signs of mitochondrial/ neurotransmitter abnormalities** | **Severity of the GDD/ID** | **Disease course** | **Reference** |
| *CACNA1F* | ID and ASD | Retinitis pigmentosa | 300110 | Inherited  p.Ile745Thr | Missense | Unknown | Disease causing | Unknown | Not clear | Profound ID | Not clear | ^56^ |
| *CACNA1F* | ID and EP | Klinefelters and retinitis pigmentosa | 300110 | Inherited  p.Ile745Thr | Missense | Unknown | Disease causing | Unknown | Not clear | Profound ID | Not clear | ^56^ |
| *CACNA1F* | ID | Congenital nystagmus | 300110 | Inherited  p.Ile745Thr | Missense | Unknown | Disease causing | Unknown | Not clear | Mild ID | Not clear | ^56^ |
| *CACNA1F* | ID | Congenital nystagmus | 300110 | Inherited  p.Ile745Thr | Missense | Unknown | Disease causing | Unknown | Not clear | Profound ID | Not clear | ^56^ |
| *CACNA1F* | ID and ASD | Rod cone dystrophy | 300110 | Inherited  p.Ile745Thr | Missense | Unknown | Disease causing | Unknown | Not clear | Profound ID | Not clear | ^56^ |
| *CACNA1F* | ID and EP | Myopia | 300110 | Inherited  p.Ile745Thr | Missense | Unknown | Disease causing | Unknown | Not clear | Mild ID | Not clear | ^56^ |
| *CACNA1I* | GDD and EP | Cortical blindness, severe proximal muscular hypotonia and distal muscular hypertonia | 607904 | De novo p.Ile860Asn | Missense | GOF | Deleterious | Frontal brain atrophy, flattened brainstem and delayed myelination | Not clear | Severe | Not clear | ^57^ |
| **Names of the gene** | **Syndrome/**  **phenotype** | **Other clinical features/ organs affected** | **OMIM numbers** | **Inheritance/Nucleotide or Protein change** | **Type of mutation** | **Altered protein function** | **Silico modeling prediction/ACMG classification** | **MRI results** | **Biochemical signs of mitochondrial/ neurotransmitter abnormalities** | **Severity of the GDD/ID** | **Disease course** | **Reference** |
| *CACNA1I* | GDD and EP | Hypotonia, severe speech impairment, feeding difficulties, and visual impairment | 607904 | De novo  p.Ile1306Thr | Missense | GOF | Deleterious | Normal | Not clear | Severe | Not clear | ^57^ |
| *CACNA1I* | GDD and EP | Hypotonia, severe speech impairment, feeding difficulties and visual impairment | 607904 | Unknown  p.Met1425Ile | Missense | GOF | Deleterious | Several brain malformations | Not clear | Severe | Not clear | ^57^ |
| *CACNA1I* | ID and EP | None | 607904 | Inherited  p.Ile860Met | Missense | GOF | Deleterious | Unknown | Not clear | Mild | Not clear | ^57^ |
| *CACNA1I* | ID | None | 607904 | Inherited  p.Ile860Met | Missense | GOF | Deleterious | Unknown | Not clear | Mild | Not clear | ^57^ |
| *CACNA1I* | ID | Speech retardation | 607904 | Inherited  p.Ile860Met | Missense | GOF | Deleterious | Unknown | Not clear | Moderate | Not clear | ^57^ |
| *CACNA1H* | ID and EP | Ventral septal defect | 607904 | De novo  p.Arg1892His | Missense | Unknown | Disease causing | Unknown | Not clear | Mild | Not clear | ^58^ |
| *CACNA1H* | ID and EP | Ventral septal defect | 607904 | De novo  p.Arg1892His | Missense | Unknown | Pathogenic | Encephalomalacic change of right temporal areas | Not clear | Mild | Not clear | ^59^ |

**Abbreviations**: ADHD; attention deficit hyperactive disorder, ASD; autism spectrum disorder, DEE; developmental and epileptic encephalopathy, EE; epileptic encephalopathy, EP; epilepsy, EIEE; early infantile epileptic encephalopathy, EOEE; early onset epileptic encephalopathy, GDD; global developmental delay, GOF: gain-of-function, ID; intellectual disability, LGS; Lennox-gastaut syndrome, LOF: loss-of-function, MRI; magnetic resonance imaging, TS; Timothy Syndrome.

**References**

1. Guerin AA, Feigenbaum A, Donner EJ, Yoon G. Stepwise developmental regression associated with novel CACNA1A mutation. *Pediatr Neurol*. 2008;39(5):363-364. doi:10.1016/j.pediatrneurol.2008.07.030

2. Yamamoto T, Imaizumi T, Yamamoto-Shimojima K, et al. Genomic backgrounds of Japanese patients with undiagnosed neurodevelopmental disorders. *Brain Dev*. 2019;41(9):776-782. doi:10.1016/j.braindev.2019.05.007

3. Mantuano E, Romano S, Veneziano L, et al. Identification of novel and recurrent CACNA1A gene mutations in fifteen patients with episodic ataxia type 2. *J Neurol Sci*. 2010;291(1-2):30-36. doi:10.1016/j.jns.2010.01.010

4. Freilinger T, Ackl N, Ebert A, et al. A novel mutation in CACNA1A associated with hemiplegic migraine, cerebellar dysfunction and late-onset cognitive decline. *J Neurol Sci*. 2011;300(1-2):160-163. doi:10.1016/j.jns.2010.09.032

5. Garcia Segarra N, Gautschi I, Mittaz-Crettol L, et al. Congenital ataxia and hemiplegic migraine with cerebral edema associated with a novel gain of function mutation in the calcium channel CACNA1A. *J Neurol Sci*. 2014;342(1-2):69-78. doi:10.1016/j.jns.2014.04.027

6. Vahedi K, Denier C, Ducros A, et al. CACNA1A gene de novo mutation causing hemiplegic migraine, coma, and cerebellar atrophy. *Neurology*. 2000;55(7):1040-1042. doi:10.1212/wnl.55.7.1040

7. Kashimada A, Hasegawa S, Nomura T, et al. Genetic analysis of undiagnosed ataxia-telangiectasia-like disorders. *Brain Dev*. 2019;41(2):150-157. doi:10.1016/j.braindev.2018.09.007

8. Naik S, Pohl K, Malik M, Siddiqui A, Josifova D. Early-onset cerebellar atrophy associated with mutation in the CACNA1A gene. *Pediatr Neurol*. 2011;45(5):328-330. doi:10.1016/j.pediatrneurol.2011.08.002

9. Weyhrauch DL, Ye D, Boczek NJ, et al. Whole Exome Sequencing and Heterologous Cellular Electrophysiology Studies Elucidate a Novel Loss-of-Function Mutation in the CACNA1A-Encoded Neuronal P/Q-Type Calcium Channel in a Child With Congenital Hypotonia and Developmental Delay. *Pediatr Neurol*. 2016;55:46-51. doi:10.1016/j.pediatrneurol.2015.10.014

10. Kothur K, Holman K, Farnsworth E, et al. Diagnostic yield of targeted massively parallel sequencing in children with epileptic encephalopathy. *Seizure*. 2018;59:132-140. doi:10.1016/j.seizure.2018.05.005

11. Angelini C, Van Gils J, Bigourdan A, et al. Major intra-familial phenotypic heterogeneity and incomplete penetrance due to a CACNA1A pathogenic variant. *Eur J Med Genet*. 2019;62(6):103530. doi:10.1016/j.ejmg.2018.08.011

12. Reinson K, Oiglane-Shlik E, Talvik I, et al. Biallelic CACNA1A mutations cause early onset epileptic encephalopathy with progressive cerebral, cerebellar, and optic nerve atrophy. *Am J Med Genet A*. 2016;170(8):2173-2176. doi:10.1002/ajmg.a.37678

13. Balck A, Hanssen H, Hellenbroich Y, Lohmann K, Munchau A. Adult-onset ataxia or developmental disorder with seizures: two sides of missense changes in CACNA1A. *J Neurol*. 2017;264(7):1520-1522. doi:10.1007/s00415-017-8494-z

14. Tantsis EM, Gill D, Griffiths L, et al. Eye movement disorders are an early manifestation of CACNA1A mutations in children. *Dev Med Child Neurol*. 2016;58(6):639-644. doi:10.1111/dmcn.13033

15. Humbertclaude V, Riant F, Krams B, et al. Cognitive impairment in children with CACNA1A mutations. *Dev Med Child Neurol*. 2020;62(3):330-337. doi:10.1111/dmcn.14261

16. Blumkin L, Michelson M, Leshinsky-Silver E, Kivity S, Lev D, Lerman-Sagie T. Congenital ataxia, mental retardation, and dyskinesia associated with a novel CACNA1A mutation. *J Child Neurol*. 2010;25(7):892-897. doi:10.1177/0883073809351316

17. Fitzsimons RB, Wolfenden WH. Migraine coma. Meningitic migraine with cerebral oedema associated with a new form of autosomal dominant cerebellar ataxia. *Brain*. 1985;108 ( Pt 3:555-577. doi:10.1093/brain/108.3.555-a

18. Kors EE, Terwindt GM, Vermeulen FL, et al. Delayed cerebral edema and fatal coma after minor head trauma: role of the CACNA1A calcium channel subunit gene and relationship with familial hemiplegic migraine. *Ann Neurol*. 2001;49(6):753-760. doi:10.1002/ana.1031

19. Wada T, Kobayashi N, Takahashi Y, Aoki T, Watanabe T, Saitoh S. Wide clinical variability in a family with a CACNA1A T666m mutation: hemiplegic migraine, coma, and progressive ataxia. *Pediatr Neurol*. 2002;26(1):47-50. doi:10.1016/s0887-8994(01)00371-x

20. de Vries B, Stam AH, Beker F, et al. CACNA1A mutation linking hemiplegic migraine and alternating hemiplegia of childhood. *Cephalalgia*. 2008;28(8):887-891. doi:10.1111/j.1468-2982.2008.01596.x

21. Damaj L, Lupien-Meilleur A, Lortie A, et al. CACNA1A haploinsufficiency causes cognitive impairment, autism and epileptic encephalopathy with mild cerebellar symptoms. *Eur J Hum Genet*. 2015;23(11):1505-1512. doi:10.1038/ejhg.2015.21

22. Indelicato E, Nachbauer W, Karner E, et al. The neuropsychiatric phenotype in CACNA1A mutations: a retrospective single center study and review of the literature. *Eur J Neurol*. 2019;26(1):66-e7. doi:10.1111/ene.13765

23. Jiang X, Raju PK, D’Avanzo N, et al. Both gain-of-function and loss-of-function de novo CACNA1A mutations cause severe developmental epileptic encephalopathies in the spectrum of Lennox-Gastaut syndrome. *Epilepsia*. 2019;60(9):1881-1894. doi:10.1111/epi.16316

24. Jung J, Testard H, Tournier-Lasserve E, et al. Phenotypic variability of episodic ataxia type 2 mutations: a family study. *Eur Neurol*. 2010;64(2):114-116. doi:10.1159/000315145

25. Luo X, Rosenfeld JA, Yamamoto S, et al. Clinically severe CACNA1A alleles affect synaptic function and neurodegeneration differentially. *PLoS Genet*. 2017;13(7):e1006905. doi:10.1371/journal.pgen.1006905

26. Tyagi S, Bendrick TR, Filipova D, Papadopoulos S, Bannister RA. A mutation in Ca(V)2.1 linked to a severe neurodevelopmental disorder impairs channel gating. *J Gen Physiol*. 2019;151(6):850-859. doi:10.1085/jgp.201812237

27. Meloche J, Brunet V, Gagnon P-A, et al. Exome sequencing study of partial agenesis of the corpus callosum in men with developmental delay, epilepsy, and microcephaly. *Mol Genet genomic Med*. 2020;8(1):e992. doi:10.1002/mgg3.992

28. Epperson M V, Haws ME, Standridge SM, Gilbert DL. An Atypical Rett Syndrome Phenotype Due to a Novel Missense Mutation in CACNA1A. *J Child Neurol*. 2018;33(4):286-289. doi:10.1177/0883073818754987

29. Ohba C, Osaka H, Iai M, et al. Diagnostic utility of whole exome sequencing in patients showing cerebellar and/or vermis atrophy in childhood. *Neurogenetics*. 2013;14(3-4):225-232. doi:10.1007/s10048-013-0375-8

30. Bertholon P, Chabrier S, Riant F, Tournier-Lasserve E, Peyron R. Episodic ataxia type 2: unusual aspects in clinical and genetic presentation. Special emphasis in childhood. *J Neurol Neurosurg Psychiatry*. 2009;80(11):1289-1292. doi:10.1136/jnnp.2008.159103

31. De Novo Mutations in SLC1A2 and CACNA1A Are Important Causes of Epileptic Encephalopathies. *Am J Hum Genet*. 2016;99(2):287-298. doi:10.1016/j.ajhg.2016.06.003

32. Bahamonde MI, Serra SA, Drechsel O, et al. A Single Amino Acid Deletion (ΔF1502) in the S6 Segment of CaV2.1 Domain III Associated with Congenital Ataxia Increases Channel Activity and Promotes Ca2+ Influx. *PLoS One*. 2015;10(12):e0146035. doi:10.1371/journal.pone.0146035

33. Dufendach KA, Timothy K, Ackerman MJ, et al. Clinical Outcomes and Modes of Death in Timothy Syndrome: A Multicenter International Study of a Rare Disorder. *JACC Clin Electrophysiol*. 2018;4(4):459-466. doi:10.1016/j.jacep.2017.08.007

34. Splawski I, Timothy KW, Decher N, et al. Severe arrhythmia disorder caused by cardiac L-type calcium channel mutations. *Proc Natl Acad Sci U S A*. 2005;102(23):8088-8089. doi:10.1073/pnas.0502506102

35. Landstrom AP, Boczek NJ, Ye D, et al. Novel long QT syndrome-associated missense mutation, L762F, in CACNA1C-encoded L-type calcium channel imparts a slower inactivation tau and increased sustained and window current. *Int J Cardiol*. 2016;220:290-298. doi:10.1016/j.ijcard.2016.06.081

36. Boczek NJ, Miller EM, Ye D, et al. Novel Timothy syndrome mutation leading to increase in CACNA1C window current. *Hear Rhythm*. 2015;12(1):211-219. doi:10.1016/j.hrthm.2014.09.051

37. Splawski I, Timothy KW, Sharpe LM, et al. Ca(V)1.2 calcium channel dysfunction causes a multisystem disorder including arrhythmia and autism. *Cell*. 2004;119(1):19-31. doi:10.1016/j.cell.2004.09.011

38. Diep V, Seaver LH. Long QT syndrome with craniofacial, digital, and neurologic features: Is it useful to distinguish between Timothy syndrome types 1 and 2? *Am J Med Genet A*. 2015;167A(11):2780-2785. doi:10.1002/ajmg.a.37258

39. Kosaki R, Ono H, Terashima H, Kosaki K. Timothy syndrome-like condition with syndactyly but without prolongation of the QT interval. *Am J Med Genet A*. 2018;176(7):1657-1661. doi:10.1002/ajmg.a.38833

40. Bozarth X, Dines JN, Cong Q, et al. Expanding clinical phenotype in CACNA1C related disorders: From neonatal onset severe epileptic encephalopathy to late-onset epilepsy. *Am J Med Genet A*. 2018;176(12):2733-2739. doi:10.1002/ajmg.a.40657

41. Hennessey JA, Boczek NJ, Jiang Y-H, et al. A CACNA1C variant associated with reduced voltage-dependent inactivation, increased CaV1.2 channel window current, and arrhythmogenesis. *PLoS One*. 2014;9(9):e106982. doi:10.1371/journal.pone.0106982

42. Edvardson S, Oz S, Abulhijaa FA, et al. Early infantile epileptic encephalopathy associated with a high voltage gated calcium channelopathy. *J Med Genet*. 2013;50(2):118-123. doi:10.1136/jmedgenet-2012-101223

43. Butler KM, Holt PJ, Milla SS, da Silva C, Alexander JJ, Escayg A. Epileptic Encephalopathy and Cerebellar Atrophy Resulting from Compound Heterozygous CACNA2D2 Variants. *Case Rep Genet*. 2018;2018:6308283. doi:10.1155/2018/6308283

44. Pippucci T, Parmeggiani A, Palombo F, et al. A novel null homozygous mutation confirms CACNA2D2 as a gene mutated in epileptic encephalopathy. *PLoS One*. 2013;8(12):e82154. doi:10.1371/journal.pone.0082154

45. Punetha J, Karaca E, Gezdirici A, et al. Biallelic CACNA2D2 variants in epileptic encephalopathy and cerebellar atrophy. *Ann Clin Transl Neurol*. 2019;6(8):1395-1406. doi:10.1002/acn3.50824

46. Vergult S, Dheedene A, Meurs A, et al. Genomic aberrations of the CACNA2D1 gene in three patients with epilepsy and intellectual disability. *Eur J Hum Genet*. 2015;23(5):628-632. doi:10.1038/ejhg.2014.141

47. Siddique A, Willoughby J, McNeill A. A 7q21.11 microdeletion presenting with apparent intellectual disability without epilepsy. *Am J Med Genet A*. 2017;173(4):1128-1130. doi:10.1002/ajmg.a.38136

48. Garza-Lopez E, Lopez JA, Hagen J, Sheffer R, Meiner V, Lee A. Role of a conserved glutamine in the function of voltage-gated Ca(2+) channels revealed by a mutation in human CACNA1D. *J Biol Chem*. 2018;293(37):14444-14454. doi:10.1074/jbc.RA118.003681

49. Pinggera A, Mackenroth L, Rump A, et al. New gain-of-function mutation shows CACNA1D as recurrently mutated gene in autism spectrum disorders and epilepsy. *Hum Mol Genet*. 2017;26(15):2923-2932. doi:10.1093/hmg/ddx175

50. Pinggera A, Lieb A, Benedetti B, et al. CACNA1D de novo mutations in autism spectrum disorders activate Cav1.3 L-type calcium channels. *Biol Psychiatry*. 2015;77(9):816-822. doi:10.1016/j.biopsych.2014.11.020

51. Flanagan SE, Vairo F, Johnson MB, et al. A CACNA1D mutation in a patient with persistent hyperinsulinaemic hypoglycaemia, heart defects, and severe hypotonia. *Pediatr Diabetes*. 2017;18(4):320-323. doi:10.1111/pedi.12512

52. Hofer NT, Tuluc P, Ortner NJ, et al. Biophysical classification of a CACNA1D de novo mutation as a high-risk mutation for a severe neurodevelopmental disorder. *Mol Autism*. 2020;11(1):4. doi:10.1186/s13229-019-0310-4

53. Helbig KL, Lauerer RJ, Bahr JC, et al. De Novo Pathogenic Variants in CACNA1E Cause Developmental and Epileptic Encephalopathy with Contractures, Macrocephaly, and Dyskinesias. *Am J Hum Genet*. 2018;103(5):666-678. doi:10.1016/j.ajhg.2018.09.006

54. Chemin J, Siquier-Pernet K, Nicouleau M, et al. De novo mutation screening in childhood-onset cerebellar atrophy identifies gain-of-function mutations in the CACNA1G calcium channel gene. *Brain*. 2018;141(7):1998-2013. doi:10.1093/brain/awy145

55. Barresi S, Dentici ML, Manzoni F, et al. Infantile-Onset Syndromic Cerebellar Ataxia and CACNA1G Mutations. *Pediatr Neurol*. 2020;104:40-45. doi:10.1016/j.pediatrneurol.2019.09.005

56. Hope CI, Sharp DM, Hemara-Wahanui A, et al. Clinical manifestations of a unique X-linked retinal disorder in a large New Zealand family with a novel mutation in CACNA1F, the gene responsible for CSNB2. *Clin Experiment Ophthalmol*. 2005;33(2):129-136. doi:10.1111/j.1442-9071.2005.00987.x

57. El Ghaleb Y, Schneeberger PE, Fernández-Quintero ML, et al. CACNA1I gain-of-function mutations differentially affect channel gating and cause neurodevelopmental disorders. *Brain*. March 2021. doi:10.1093/brain/awab101

58. Han JY, Jang JH, Park J, Lee IG. Targeted Next-Generation Sequencing of Korean Patients With Developmental Delay and/or Intellectual Disability. *Front Pediatr*. 2018;6:391. doi:10.3389/fped.2018.00391

59. Han JY, Jang W, Park J, Kim M, Kim Y, Lee IG. Diagnostic approach with genetic tests for global developmental delay and/or intellectual disability: Single tertiary center experience. *Ann Hum Genet*. 2019;83(3):115-123. doi:10.1111/ahg.12294
