# Supplementary material for: Calcium channelopathies and intellectual disability: a systematic review
Source: Orphanet J Rare Dis. 2021 May 13;16:219. doi: 10.1186/s13023-021-01850-0 (PMC8120735; doi:10.1186/s13023-021-01850-0)
Supplement: Supplementary file 3 — Additional file 3. [file 13023_2021_1850_MOESM3_ESM.docx]

**1 Genotype-phenotype list**

**1.1 *CACNA1A***

*CACNA1A* (calcium voltage-gated channel subunit alpha1 A) encodes the alpha-1 subunit of a calcium channel called Cav2.1 (P/Q-type)(1). P/Q-type channels are distributed widely throughout the CNS, with high expression levels in the cerebral cortex, hippocampus, and cerebellum (2–4). Four domains of alpha 1A subunit form the conducting pore, while β and α2δ subunits (accessory) moderate channel kinetics and expression levels. They are found in the pre-synaptic area, where they stimulate the secretion of neurotransmitters and thus accelerate synaptic transmission (5). P/Q-type current facilitates short-term synaptic plasticity through the activation of neuronal Ca (2+) sensor proteins (CaS) and markedly reduce synaptic transmission by deleting the calmodulin-binding domain (inhibitor of CaS-dependent inactivation) (6).

*CACNA1A* variants are associated with a wide range of phenotypes including ID, epilepsy, migraine, ataxia, cerebellar atrophy, episodic ataxia (7,8,17–26,9,27–30,10–16) atypical Rett syndrome (31), and early infantile epileptic encephalopathy (8,14,27,32,33). The reported mutations have different underlying mechanisms. *De novo* p.Phe1502del showed gain-of-function property (it was activated at lower voltage threshold than the wild type) (34). *De novo* p.Ala713Thr revealed gain-of-function effect with facilitated current activation, increased whole-cell currents and slower current decay (27). Likewise, p.Val1396Met and p.Gly2314Ser resulted to gain-of-function (increased whole-cell currents and facilitated current activation) (27). *De* novo p.Pro1353Leu showed loss-of-function effect (95% reduction in peak current density) (15). p.Arg158Thrfs*6 which was found in two siblings led to truncation of the protein in the exon three. The electrophysiological studies for *de novo* p.Gly230Val and *de novo* p.Ile1357Ser showed reduced whole-cell current densities and decreased channel expression at the cell membrane)(27). *De novo* p.Arg1673Pro was suggested to cause gain-of-function property in *Drosophila cacophony* larvae, however, it was later confirmed to produce loss-of-function effect (25-mV depolarizing shift in the voltage dependence of activation) (35). This variant led to minimal calcium entrance in the cell of which could be rescued by GV-58 (35). Therefore, both gain- and loss-of-function variants (**Figure 1**) of this gene are associated with ID, leading to a range of mild-to-severe phenotypes depending on the variant (**Additional file 2:** **Table S1**).

Calcium channel blockers (verapamil) showed partial efficacy in the treatment of severe hemiplegic migraine in cases with gain-of function variants, however, no comment was given regarding its effects on ID (17). A conditional (forebrain specific) Cav2.1 knock-out mouse model reportedly exhibited an impairment of synaptic transmission at hippocampal glutamatergic synapses as well as deficits in spatial learning and memory (36). Knock-in mice expressing Arg192Gln (gain-of-function mutation), which is associated with migraine and ID, revealed enhanced hippocampal excitatory transmission and long-term potentiation; by contrast, learning and memory were impaired (37). This study demonstrated how unexpectedly changes in plasticity can affect learning and how heightened neuronal excitability may lead to ID (37). A Cav2.1-channel mutant, the heterozygous leaner mouse *(tg(la)/+)* demonstrated cognitive and motor deficits (38). Ifenprodil (a selective blocker of NMDAR) can block P/Q type calcium channels, leading to the reduction of presynaptic excitatory synaptic transmission (39). The blockage of P/Q type calcium channels by D1-like dopamine receptors leads to low glutamate release into the cholinergic basal forebrain neurons of immature mice (40,41). P/Q-type Ca (2+) channels are also involved in the cAMP/protein kinase pathway that facilitates the release of GABA from striatal terminals following the facilitation of depolarization by a dopamine D1 agonist (42). The Cav2.1 mutation impairs GABAergic inhibition, resulting in abnormal discharges in the hippocampi of epileptic mice without *tg* (43). Considered together, the aforementioned findings from human and animal studies implicate both gain- and loss-of- function variants in the development of ID. Future research should consider how both gain- and loss-of-function variants can result in ID.

**1.2 *CACNA1C***

*CACNA1C* (calcium voltage-gated channel subunit alpha1 C) encodes the alpha-1 subunit of a calcium channel called Cav1.2 (L-type), which is characterized by slow voltage-dependent inactivation and can be blocked by calcium antagonists (44). Found in neuronal cell bodies and proximal dendritic spines,(45). Cav1.2 regulates gene expression by activating the cAMP response element-binding protein (CREB) and gene transcription by activating brain-derived neurotrophic factor (BDNF), which is essential to long-term potentiation (46,47).

Cav1.2 knockdown mice models demonstrated abridged CREB transcription and long-term potentiation in their hippocampi (48,49). Blockers of this channel induced the reduction of long-term potentiation in the CA1 of the mouse hippocampus (50). Moreover, as the conditional deletion of Cav1.2 impairs remyelination in mice (51,52), it is crucial for the remyelination of neurons. The postnatal rescue of abnormal corticogenesis in immature mouse neurons by correcting L-type signal aberrations demonstrates the importance of this channel to neuronal growth and cortical migration (53). Cav1.2 channels are regulated by phosphoprotein phosphatases and protein kinases (54), and beta 2-adrenergic receptors interact with Cav1.2 channels to control their activity and consequently affect long-term postsynaptic plasticity (55,56).The protein densin-180 augments the cell surface trafficking and postsynaptic localization of Cav1.2 which in turn aid in activity-dependent gene transcription (57).

*CACNA1C* variants have been associated with Timothy Syndrome, which is characterized by GDD, prolonged QT interval, epilepsy, hypoglycemia, abnormal behaviours, syndactyly, and immunodeficiency (58–63). Moreover, they have been linked to the neonatal onset epileptic encephalopathy and ID with late-onset epilepsy (64). *De novo* p.Gly406Arg and *de novo* Gly402Ser variants caused a complete loss of voltage-dependent channel inactivation thus leading to calcium overload inside the cells (58,65). *De novo* p.Ile1166Thr led to loss of current density and a gain-of-function shift in activation, hence an increased window current (62). Thus, it seems Timothy syndrome occurs due to slowing of channel inactivation (**Figure 2**).

*Cacna1c−/−* knock-out mice models exhibit impaired learning and memory (48,66,67). While gain-of-function variants seem to explain the onset of ID, there is no knock-in mouse at present. The transgenic mouse model (BACHD mice) of Huntington’s disease demonstrated high Cav1.2 protein levels in the cortex as well as increased amount of L-type calcium currents as compared with wild-type mice (68). Besides, neuronal death was observed *in vitro* and could be rescued by Cav1.2 blockers (68). Over expression of Cav1.2 in hypothyroid adult mice resulted to memory deficits of which could be ameliorated by Omega-3 supplementation suggesting that Omega-3 has neuroprotective effect (69). On the other hand, loss-of-function variants could also be implicated in ID because *Cacna1c−/−* knock-out mice exhibited impaired learning and memory (48,66,67). The forebrain Cav1.2 knockout mice demonstrated heightened cell death of young hippocampal neurons and could be prevented by P7C3-A20 (neuroprotective agent) implying that Cav1.2 plays a critical role in the development of hippocampal neurons (70). The mouse with an inactivation of the Cav1.2 gene in the hippocampus and frontal cortex (Cav1.2 (HCKO)) exhibited severe deficiency of hippocampus-dependent spatial memory (48). However, the recent assessment of spatial learning, reversal learning and object recognition memory in heterozygous *Cacna1c*+/- rats and *Cacna1c*+/+ littermate controls revealed *Cacna1c* haploinsufficiency has a minor effect on spatial memory functions in mice (71). Similarly, *Cacna1c* deletion model for autism spectrum disorder did not exhibit cognitive deficits (72). Another study revealed that *Cacna1c* knockout does not affect learning universally (73). Noteworthy, one study showed that embryonic deletion of *Cacna1c* in cortical glutamatergic neurons led to cognitive decline, defects in synaptic plasticity, hyperactivity, anxiety and reduced sociability, nevertheless, these abnormalities were not noticed in adult mice suggesting that *CACNA1C* is important for neurodevelopment (74).

**1. 3 *CACNA1D***

Also known as the L-type calcium channel, *CACNA1D* (calcium voltage-gated channel subunit alpha1 D) provides instructions for making one part (the alpha-1 subunit) of a calcium channel called Cav1.3. They are found in neuronal cell bodies and proximal dendritic spines (45). They are characterized by slow voltage-dependent inactivation and can be blocked by calcium antagonists(44). *CACNA1D* variants are reportedly related with ID, epilepsy, autism spectrum disorder, and congenital hyperinsulinaemic hypoglycemia (75,76). All reported variants including Val401Leu, Ala749Gly, Ser652Leu, Gly407Arg, and Gly403Asp,Gln567His (75–77) induced mild-to-profound ID (**Additional file 2: Table S1)**. They exhibited gain-of function effects (Figure 3).

The expression of L-type channels increases with aging in mice and correlates inversely with memory and learning (78). Likewise, young mice with the 50% overexpression of Cav1.3 in the forebrain displayed cognitive deficits (79). Cav1.3 channels regulate D2-autoreceptor responses through neuronal calcium sensor-1 in substantia nigra dopamine neurons, and Cav1.3 blockers have a protective effect against Parkinson’s disease (80,81). Excessive Cav1.3 calcium channel activation in rat hippocampal and cortical cultures reportedly results in cell death caused by mitochondrial dysfunction (82).

**1.4 *CACNA1E***

*CACNA1E* (calcium voltage-gated channel subunit alpha1 E) encodes the alpha-1E subunit of the R-type calcium channel, which is also called Cav2.3, (83) found in presynaptic terminals. Cav2.3 interacts with N-ethylmaleimide-sensitive factor-activating protein receptor (SNARE) proteins to induce neurotransmitter release and is involved in long-term potentiation (83,84) . R-type calcium channels are characterized by faster voltage-dependent inactivation (54). The coupling of Cav2.3 and BK potassium channels regulates short-term plasticity in the **mouse hippocampus,** (85) and the presynaptic Cav2.3 channels lead to fast excitatory synaptic (glutamatergic) transmission in the rat’s hippocampus (86).

Variants of this gene have been identified in cases of severe-to-profound ID and epileptic encephalopathy (87). Reported alleles include Leu228Pro, Gly348Arg, Gly352Arg, Ile603Leu, Gly690Asp, Phe698Ser, Ala700Thr, Ile701Val, Ala702Thr, Ile1422Phe, Thr1425Asn, Gly1430Arg, and Ala1720Gly: all of which induced gain-of-function effects (87).These variants facilitate voltage-dependent activation and slow inactivation, indicating their critical role in regulating calcium channel gating (87). **Figure 4** depicts their locations.

Over expression of Cav2.3 in FMRP KO mice leads to Fragile X syndrome (FXS) (88). Cav2.3 plays a critical role in hippocampal synchronization associated with theta oscillation (key elements in cognitive processes) (43,89). Cav2.3-deficient mice evince abnormal thalamocortical rhythmicity and exhibit augmented absence seizure susceptibility (90). The upregulation of Cav2.3 channels results in the co-activation of group I metabotropic glutamate receptors (mGluRs) and muscarinic acetylcholine receptors (mAChRs), which in turn surges the excitability of hippocampal CA1 pyramidal neurons in cognitive processes (91). We speculate that the excessive expression of Cav2.3 channels may induce ID.

**1.5 *CACNA1F***

*CACNA1F* (calcium voltage-gated channel subunit alpha1 F) encodes the alpha-1 subunit of voltage-gated calcium channels (also known as Cav1.4 or L-type) (92,93). They are chiefly expressed in the presynaptic membrane of photoreceptors, where they facilitate the release of glutamate (92–95). In addition, Cav1.4 channels are expressed in hippocampus and cerebellum. (96,97).They are found in neuronal cell bodies and proximal dendritic spines (45) and are characterized by slow voltage-dependent inactivation. They can be blocked with calcium antagonists, including phenylalkylamines and dihydropyridines (44).

*CACNA1F* alleles are associated with X-linked cone rod dystrophy and incomplete X-linked congenital stationary night blindness type 2 (98). Hope et al. (2005) documented eight patients who presented with ID, epilepsy, autism spectrum disorders, and different eye anomalies (cortical blindness, Lebers congenital, retinitis pigmentosa, Klinefelters and retinitis pigmentosa, congenital nystagmus, rod cone dystrophy and myopia). Of those eight patients, six presented with profound ID (99). p.Ile745Thr was found in all eight cases, nevertheless, no functional study was carried out (99). While the Cav1.4 knock-out mouse retina salvaged synaptic development, no subsequently performed cognition tests were reported (100). Surprisingly, IT mouse model carrying gain-of-function mutation for the congenital stationary night blindness type 2 did not show deficits in learning and memory (101). Nevertheless, the development of a knock-in-mice model carrying mutation associated with ID is needed to study how gain-of-function variants contribute to ID development.

**1.6 *CACNA1G***

*CACNA1G* (calcium voltage-gated channel subunit alpha1) encodes low-voltage activated calcium channels (also called Cav3.1 or T-type channels). They are highly expressed in the cerebellum, hippocampus, and thalamus, where they are responsible for postsynaptic calcium signaling and thus contribute to long-term potentiation (102,103). In addition, T-type channels are found in the dendrites, spines, and soma of neurons throughout the central nervous system. They can be blocked with Mibefradil and TTA-P2 (103). T-type current may enhance dendritic depolarization (increase neuronal excitability) or, on the other hand, can stimulate calcium-activated potassium currents, resulting in membrane hyperpolarisations (104).

*CACNA1G* alleles are reportedly associated with ID, epileptic, ataxic, dysmorphic, and ophthalmic features (105,106). Eight patients who presented with severe-to-profound ID (Ala961Thr and Met1531Val) have been documented. These variants impaired channel inactivation with significantly slower kinetics and negatively shifted potential for half-inactivation (**Figure 5**). In addition, 3 cases with ID and cataracts carrying Ser1346fs have been reported (107). This variant was predicted to abolish the function of the gene.

Cav3.1 knock-out mice exhibit impaired cerebellum-dependent motor learning and synaptic plasticity, suggesting that Cav3.1 current is important for long-term potentiation (102). Yabuki et al. (2017) demonstrated that Cav3.1 channels enhance hippocampal acetylcholine release, which improves cognition (108). Deletion of Cav3.1 channel suppresses long-term potentiation in the CA1-subiculum (109). Altogether, the aforementioned results indicate the importance of Cav3.1 channels in learning and memory and that loss-of-function variants can impair cognition. Nevertheless, each of the eight reported cases involved gain-of-function variants, implying that even excessive neuronal excitability can induce ID.

**1.7 *CACNA1H***

*CACNA1H* (calcium voltage-gated channel subunit alpha1 H) encodes for low voltage-gated T-type calcium channel alpha subunit Cav3.2. They are more expressed in the thalamus, hippocampus, cortex and cerebellum(110). They regulate neuronal excitability, neuronal and axonal firing, hormonal secretion, neurotransmitter release, sleeping cycles, feeding behaviour, peace-making in the heart, the renin-angiotensin system, relaxation of cerebral arteries and gene transcription(110). Two cases with mild ID, epilepsy and ventral septal defect have been reported(111). They both carried *de novo* p.Arg1892His which was predicted to be pathogenic(111). Cav3.2 knockout mice exhibited impaired bursting and synaptic plasticity(112). Studies from mice have shown that retrieval of memory depends on Cav3.2 channels(113). Besides, they play a major role in short term plasticity(114).

**1.8 *CACNA1I***

CACNA1I (calcium voltage-gated channel subunit alpha1 I) encodes for low-voltage activated calcium channels (also called Cav3.3 or T-type channels). These channels are highly expressed in the brain(115) especially in cerebellum, thalamus, and cerebral cortex(116). They regulate neuronal excitability, neurotransmitter, and hormone release(117). In the past, *CACNA1I* was reported to associate with schizophrenia (115,118). Nevertheless, three cases with mild to severe ID, epilepsy and multiple congenital anomalies were reported recently (119). Responsible variants were (p.Ile860Asn, p.Ile1306Thr, and p.Met1425Ile) with gain-of-function effects (**Figure 6**). Electrophysiological studies revealed increased window currents leading to augmented calcium influx(119). As a result, authors concluded that calcium toxicity in neurons is likely to be the cause of the ID(119). The co-activation of Cav3.3 and GluN2B-containing NMDA receptors mediated long-term potentiation at thalamoreticular inputs in mice(120).

**1.9 *CACNA2D1***

*CACNA2D1* (calcium voltage-gated channel auxiliary subunit alpha2delta 1) encodes a preproprotein than can be cleaved into the alpha-2 and delta 1 subunits of the voltage-dependent calcium channel complex. This gene is highly expressed in the frontal cortex (121) especially in areas involved in learning, memory, and cortical processing (122). *CACNA2D1* augments channel trafficking, increase the expression and function of the alpha 1 subunit, and contributes to synaptogenesis (123,124). In addition, alpha-2 and delta 1 subunit can interact with NMDA receptor for long-term potentiation, learning and memory(125).

Three patients with ID and epilepsy carried deletions of different sizes at 7q21.11, which spans the *CACNA2D1* and *PCLO* (Piccolo Presynaptic Cytomatrix Protein) genes (126). Of the three cases, two had malformations of cortical development (cortical atrophy and polymicrogyria). Likewise, one case with ID alone had a deletion at 7q21.11 that spanned the *CACNA2D1* and *PCLO* genes (127). *CACNA2D1* and *PCLO* genes regulate neurotransmitter release and are thus likely to account for ID. Nevertheless, *PCLO* is expressed presynaptically only, while *CACNA2D1* is expressed both presynaptically and postsynaptically (127). The overexpression of alpha-2 and delta 1 subunits in mice causes behavioral arrest, cortical hyperexcitability, epileptic discharges, cell death, and synaptogenesis that can be inhibited by gabapentin (128,129). Therefore, the deletion of the *CACNA2D1* gene can affect synaptogenesis and neurotransmitter release, both of which are crucial for learning and memory. These findings suggest that some calcium blockers (gabapentin) used to treat epilepsy and other conditions might inhibit synaptogenesis and synaptic plasticity (128,129). Hence, loss-of-function variants affecting *CACNA2D1* may account for the onset of ID. Noteworthy, ID may not be related to *CACNA2D1* but only to *PLCO*.

**1.10 *CACNA2D2***

*CACNA2D2* (calcium voltage-gated channel auxiliary subunit alpha2delta 2) encodes the alpha-2/delta subunit of the voltage-dependent calcium channel complex. They feature relatively heightened expression in the cerebellar cortex and areas that regulate the activities of the cortex, hippocampus, and thalamus (122,130). They are found in the pre-synaptic membrane, where they help synapses to make more effective use of calcium influx for the activation of neurotransmitter release by enhancing the currents produced by the alpha-1 subunit, mainly of the Cav2.1 calcium channel (131,132).

*CACNA2D2* variants have been noticed in cases of ID/GDD, epileptic encephalopathy, ataxia, and cerebellar atrophy and induced loss-of-function (p.Leu1040Pro, p.Asn432Thrfs∗35 and p.Tyr162*)(121,133–135). These mutations lead to reduced channel current density and slow inactivation (**Figure 7**). The disruption of *CACNA2D2* (tm1NCIF) in mice induced ataxia, seizure, cardiac abnormalities, premature death (132,136) and cerebellar atrophy (130,132,137). Nevertheless, no cognition tests were performed in the mice.

References

1. Bourinet E, Soong TW, Sutton K, Slaymaker S, Mathews E, Monteil A, et al. Splicing of alpha 1A subunit gene generates phenotypic variants of P- and Q-type calcium channels. Nat Neurosci. 1999 May;2(5):407–15.

2. Mintz IM, Adams ME, Bean BP. P-type calcium channels in rat central and peripheral neurons. Neuron. 1992 Jul;9(1):85–95.

3. Westenbroek RE, Sakurai T, Elliott EM, Hell JW, Starr T V, Snutch TP, et al. Immunochemical identification and subcellular distribution of the alpha 1A subunits of brain calcium channels. J Neurosci. 1995 Oct;15(10):6403–18.

4. Fletcher CF, Lutz CM, O’Sullivan TN, Shaughnessy JDJ, Hawkes R, Frankel WN, et al. Absence epilepsy in tottering mutant mice is associated with calcium channel defects. Cell. 1996 Nov;87(4):607–17.

5. Ishikawa T, Kaneko M, Shin H-S, Takahashi T. Presynaptic N-type and P/Q-type Ca2+ channels mediating synaptic transmission at the calyx of Held of mice. J Physiol. 2005 Oct;568(Pt 1):199–209.

6. Mochida S, Few AP, Scheuer T, Catterall WA. Regulation of presynaptic Ca(V)2.1 channels by Ca2+ sensor proteins mediates short-term synaptic plasticity. Neuron. 2008 Jan;57(2):210–6.

7. Guerin AA, Feigenbaum A, Donner EJ, Yoon G. Stepwise developmental regression associated with novel CACNA1A mutation. Pediatr Neurol. 2008 Nov;39(5):363–4.

8. Yamamoto T, Imaizumi T, Yamamoto-Shimojima K, Lu Y, Yanagishita T, Shimada S, et al. Genomic backgrounds of Japanese patients with undiagnosed neurodevelopmental disorders. Brain Dev. 2019 Oct;41(9):776–82.

9. Freilinger T, Ackl N, Ebert A, Schmidt C, Rautenstrauss B, Dichgans M, et al. A novel mutation in CACNA1A associated with hemiplegic migraine, cerebellar dysfunction and late-onset cognitive decline. J Neurol Sci. 2011 Jan;300(1–2):160–3.

10. Garcia Segarra N, Gautschi I, Mittaz-Crettol L, Kallay Zetchi C, Al-Qusairi L, Van Bemmelen MX, et al. Congenital ataxia and hemiplegic migraine with cerebral edema associated with a novel gain of function mutation in the calcium channel CACNA1A. J Neurol Sci. 2014 Jul;342(1–2):69–78.

11. Mantuano E, Romano S, Veneziano L, Gellera C, Castellotti B, Caimi S, et al. Identification of novel and recurrent CACNA1A gene mutations in fifteen patients with episodic ataxia type 2. J Neurol Sci. 2010 Apr;291(1–2):30–6.

12. Vahedi K, Denier C, Ducros A, Bousson V, Levy C, Chabriat H, et al. CACNA1A gene de novo mutation causing hemiplegic migraine, coma, and cerebellar atrophy. Neurology. 2000 Oct;55(7):1040–2.

13. Kashimada A, Hasegawa S, Nomura T, Shiraku H, Moriyama K, Suzuki T, et al. Genetic analysis of undiagnosed ataxia-telangiectasia-like disorders. Brain Dev. 2019 Feb;41(2):150–7.

14. Angelini C, Van Gils J, Bigourdan A, Jouk P-S, Lacombe D, Menegon P, et al. Major intra-familial phenotypic heterogeneity and incomplete penetrance due to a CACNA1A pathogenic variant. Eur J Med Genet. 2019 Jun;62(6):103530.

15. Weyhrauch DL, Ye D, Boczek NJ, Tester DJ, Gavrilova RH, Patterson MC, et al. Whole Exome Sequencing and Heterologous Cellular Electrophysiology Studies Elucidate a Novel Loss-of-Function Mutation in the CACNA1A-Encoded Neuronal P/Q-Type Calcium Channel in a Child With Congenital Hypotonia and Developmental Delay. Pediatr Neurol. 2016 Feb;55:46–51.

16. Naik S, Pohl K, Malik M, Siddiqui A, Josifova D. Early-onset cerebellar atrophy associated with mutation in the CACNA1A gene. Pediatr Neurol. 2011 Nov;45(5):328–30.

17. Tantsis EM, Gill D, Griffiths L, Gupta S, Lawson J, Maksemous N, et al. Eye movement disorders are an early manifestation of CACNA1A mutations in children. Dev Med Child Neurol. 2016 Jun;58(6):639–44.

18. Balck A, Hanssen H, Hellenbroich Y, Lohmann K, Munchau A. Adult-onset ataxia or developmental disorder with seizures: two sides of missense changes in CACNA1A. Vol. 264, Journal of neurology. Germany; 2017. p. 1520–2.

19. Humbertclaude V, Riant F, Krams B, Zimmermann V, Nagot N, Annequin D, et al. Cognitive impairment in children with CACNA1A mutations. Dev Med Child Neurol. 2020 Mar;62(3):330–7.

20. de Vries B, Stam AH, Beker F, van den Maagdenberg AMJM, Vanmolkot KRJ, Laan L, et al. CACNA1A mutation linking hemiplegic migraine and alternating hemiplegia of childhood. Cephalalgia. 2008 Aug;28(8):887–91.

21. Wada T, Kobayashi N, Takahashi Y, Aoki T, Watanabe T, Saitoh S. Wide clinical variability in a family with a CACNA1A T666m mutation: hemiplegic migraine, coma, and progressive ataxia. Pediatr Neurol. 2002 Jan;26(1):47–50.

22. Kors EE, Terwindt GM, Vermeulen FL, Fitzsimons RB, Jardine PE, Heywood P, et al. Delayed cerebral edema and fatal coma after minor head trauma: role of the CACNA1A calcium channel subunit gene and relationship with familial hemiplegic migraine. Ann Neurol. 2001 Jun;49(6):753–60.

23. Fitzsimons RB, Wolfenden WH. Migraine coma. Meningitic migraine with cerebral oedema associated with a new form of autosomal dominant cerebellar ataxia. Brain. 1985 Sep;108 ( Pt 3:555–77.

24. Blumkin L, Michelson M, Leshinsky-Silver E, Kivity S, Lev D, Lerman-Sagie T. Congenital ataxia, mental retardation, and dyskinesia associated with a novel CACNA1A mutation. J Child Neurol. 2010 Jul;25(7):892–7.

25. Damaj L, Lupien-Meilleur A, Lortie A, Riou E, Ospina LH, Gagnon L, et al. CACNA1A haploinsufficiency causes cognitive impairment, autism and epileptic encephalopathy with mild cerebellar symptoms. Eur J Hum Genet. 2015 Nov;23(11):1505–12.

26. Indelicato E, Nachbauer W, Karner E, Eigentler A, Wagner M, Unterberger I, et al. The neuropsychiatric phenotype in CACNA1A mutations: a retrospective single center study and review of the literature. Eur J Neurol. 2019 Jan;26(1):66-e7.

27. Jiang X, Raju PK, D’Avanzo N, Lachance M, Pepin J, Dubeau F, et al. Both gain-of-function and loss-of-function de novo CACNA1A mutations cause severe developmental epileptic encephalopathies in the spectrum of Lennox-Gastaut syndrome. Epilepsia. 2019 Sep;60(9):1881–94.

28. Luo X, Rosenfeld JA, Yamamoto S, Harel T, Zuo Z, Hall M, et al. Clinically severe CACNA1A alleles affect synaptic function and neurodegeneration differentially. PLoS Genet. 2017 Jul;13(7):e1006905.

29. Meloche J, Brunet V, Gagnon P-A, Lavoie M-E, Bouchard J-B, Nadaf J, et al. Exome sequencing study of partial agenesis of the corpus callosum in men with developmental delay, epilepsy, and microcephaly. Mol Genet genomic Med. 2020 Jan;8(1):e992.

30. Ohba C, Osaka H, Iai M, Yamashita S, Suzuki Y, Aida N, et al. Diagnostic utility of whole exome sequencing in patients showing cerebellar and/or vermis atrophy in childhood. Neurogenetics. 2013 Nov;14(3–4):225–32.

31. Epperson M V, Haws ME, Standridge SM, Gilbert DL. An Atypical Rett Syndrome Phenotype Due to a Novel Missense Mutation in CACNA1A. J Child Neurol. 2018 Mar;33(4):286–9.

32. Kothur K, Holman K, Farnsworth E, Ho G, Lorentzos M, Troedson C, et al. Diagnostic yield of targeted massively parallel sequencing in children with epileptic encephalopathy. Seizure. 2018 Jul;59:132–40.

33. Reinson K, Oiglane-Shlik E, Talvik I, Vaher U, Ounapuu A, Ennok M, et al. Biallelic CACNA1A mutations cause early onset epileptic encephalopathy with progressive cerebral, cerebellar, and optic nerve atrophy. Am J Med Genet A. 2016 Aug;170(8):2173–6.

34. García Segarra N, Gautschi I, Mittaz-Crettol L, Kallay Zetchi C, Al-Qusairi L, Van Bemmelen MX, et al. Congenital ataxia and hemiplegic migraine with cerebral edema associated with a novel gain of function mutation in the calcium channel CACNA1A. J Neurol Sci. 2014 Jul;342(1–2):69–78.

35. Tyagi S, Bendrick TR, Filipova D, Papadopoulos S, Bannister RA. A mutation in Ca(V)2.1 linked to a severe neurodevelopmental disorder impairs channel gating. J Gen Physiol. 2019 Jun;151(6):850–9.

36. Mallmann RT, Elgueta C, Sleman F, Castonguay J, Wilmes T, van den Maagdenberg A, et al. Ablation of Ca(V)2.1 voltage-gated Ca(2)(+) channels in mouse forebrain generates multiple cognitive impairments. PLoS One. 2013;8(10):e78598.

37. Dilekoz E, Houben T, Eikermann-Haerter K, Balkaya M, Lenselink AM, Whalen MJ, et al. Migraine mutations impair hippocampal learning despite enhanced long-term potentiation. J Neurosci. 2015 Feb;35(8):3397–402.

38. Alonso I, Marques JM, Sousa N, Sequeiros J, Olsson IAS, Silveira I. Motor and cognitive deficits in the heterozygous leaner mouse, a Cav2.1 voltage-gated Ca2+ channel mutant. Neurobiol Aging. 2008 Nov;29(11):1733–43.

39. Delaney AJ, Power JM, Sah P. Ifenprodil reduces excitatory synaptic transmission by blocking presynaptic P/Q type calcium channels. J Neurophysiol. 2012 Mar;107(6):1571–5.

40. Momiyama T, Fukazawa Y. D1-like dopamine receptors selectively block P/Q-type calcium channels to reduce glutamate release onto cholinergic basal forebrain neurones of immature rats. J Physiol. 2007 Apr;580(Pt 1):103–17.

41. Momiyama T. Developmental increase in D1-like dopamine receptor-mediated inhibition of glutamatergic transmission through P/Q-type channel regulation in the basal forebrain of rats. Eur J Neurosci. 2010 Aug;32(4):579–90.

42. Arias-Montano J-A, Floran B, Floran L, Aceves J, Young JM. Dopamine D(1) receptor facilitation of depolarization-induced release of gamma-amino-butyric acid in rat striatum is mediated by the cAMP/PKA pathway and involves P/Q-type calcium channels. Synapse. 2007 May;61(5):310–9.

43. Nakao A, Miki T, Shimono K, Oka H, Numata T, Kiyonaka S, et al. Compromised maturation of GABAergic inhibition underlies abnormal network activity in the hippocampus of epileptic Ca2+ channel mutant mice, tottering. Pflugers Arch. 2015 Apr;467(4):737–52.

44. Reuter H. Calcium channel modulation by neurotransmitters, enzymes and drugs. Nature. 1983 Feb;301(5901):569–74.

45. Hell JW, Westenbroek RE, Warner C, Ahlijanian MK, Prystay W, Gilbert MM, et al. Identification and differential subcellular localization of the neuronal class C and class D L-type calcium channel alpha 1 subunits. J Cell Biol [Internet]. 1993 Nov 15;123(4):949–62. Available from: https://doi.org/10.1083/jcb.123.4.949

46. Moon AL, Haan N, Wilkinson LS, Thomas KL, Hall J. CACNA1C: Association With Psychiatric Disorders, Behavior, and Neurogenesis. Schizophr Bull. 2018 Aug;44(5):958–65.

47. Lu B, Nagappan G, Lu Y. BDNF and synaptic plasticity, cognitive function, and dysfunction. Handb Exp Pharmacol. 2014;220:223–50.

48. Moosmang S, Haider N, Klugbauer N, Adelsberger H, Langwieser N, Muller J, et al. Role of hippocampal Cav1.2 Ca2+ channels in NMDA receptor-independent synaptic plasticity and spatial memory. J Neurosci. 2005 Oct;25(43):9883–92.

49. Striessnig J, Koschak A, Sinnegger-Brauns MJ, Hetzenauer A, Nguyen NK, Busquet P, et al. Role of voltage-gated L-type Ca2+ channel isoforms for brain function. Biochem Soc Trans. 2006 Nov;34(Pt 5):903–9.

50. Freir DB, Herron CE. Inhibition of L-type voltage dependent calcium channels causes impairment of long-term potentiation in the hippocampal CA1 region in vivo. Brain Res. 2003 Mar;967(1–2):27–36.

51. Santiago Gonzalez DA, Cheli VT, Zamora NN, Lama TN, Spreuer V, Murphy GG, et al. Conditional Deletion of the L-Type Calcium Channel Cav1.2 in NG2-Positive Cells Impairs Remyelination in Mice. J Neurosci. 2017 Oct;37(42):10038–51.

52. Cheli VT, Santiago Gonzalez DA, Namgyal Lama T, Spreuer V, Handley V, Murphy GG, et al. Conditional Deletion of the L-Type Calcium Channel Cav1.2 in Oligodendrocyte Progenitor Cells Affects Postnatal Myelination in Mice. J Neurosci. 2016 Oct;36(42):10853–69.

53. Kamijo S, Ishii Y, Horigane S-I, Suzuki K, Ohkura M, Nakai J, et al. A Critical Neurodevelopmental Role for L-Type Voltage-Gated Calcium Channels in Neurite Extension and Radial Migration. J Neurosci. 2018 Jun;38(24):5551–66.

54. Nanou E, Catterall WA. Calcium Channels, Synaptic Plasticity, and Neuropsychiatric Disease. Neuron. 2018 May;98(3):466–81.

55. Patriarchi T, Qian H, Di Biase V, Malik ZA, Chowdhury D, Price JL, et al. Phosphorylation of Cav1.2 on S1928 uncouples the L-type Ca2+ channel from the beta2 adrenergic receptor. EMBO J. 2016 Jun;35(12):1330–45.

56. Qian H, Patriarchi T, Price JL, Matt L, Lee B, Nieves-Cintron M, et al. Phosphorylation of Ser1928 mediates the enhanced activity of the L-type Ca2+ channel Cav1.2 by the beta2-adrenergic receptor in neurons. Sci Signal. 2017 Jan;10(463).

57. Wang S, Stanika RI, Wang X, Hagen J, Kennedy MB, Obermair GJ, et al. Densin-180 Controls the Trafficking and Signaling of L-Type Voltage-Gated Ca(v)1.2 Ca(2+) Channels at Excitatory Synapses. J Neurosci. 2017 May;37(18):4679–91.

58. Dufendach KA, Timothy K, Ackerman MJ, Blevins B, Pflaumer A, Etheridge S, et al. Clinical Outcomes and Modes of Death in Timothy Syndrome: A Multicenter International Study of a Rare Disorder. JACC Clin Electrophysiol. 2018 Apr;4(4):459–66.

59. Landstrom AP, Boczek NJ, Ye D, Miyake CY, De la Uz CM, Allen HD, et al. Novel long QT syndrome-associated missense mutation, L762F, in CACNA1C-encoded L-type calcium channel imparts a slower inactivation tau and increased sustained and window current. Int J Cardiol. 2016 Oct;220:290–8.

60. Boczek NJ, Miller EM, Ye D, Nesterenko V V, Tester DJ, Antzelevitch C, et al. Novel Timothy syndrome mutation leading to increase in CACNA1C window current. Hear Rhythm. 2015 Jan;12(1):211–9.

61. Splawski I, Timothy KW, Sharpe LM, Decher N, Kumar P, Bloise R, et al. Ca(V)1.2 calcium channel dysfunction causes a multisystem disorder including arrhythmia and autism. Cell. 2004 Oct;119(1):19–31.

62. Diep V, Seaver LH. Long QT syndrome with craniofacial, digital, and neurologic features: Is it useful to distinguish between Timothy syndrome types 1 and 2? Am J Med Genet A. 2015 Nov;167A(11):2780–5.

63. Kosaki R, Ono H, Terashima H, Kosaki K. Timothy syndrome-like condition with syndactyly but without prolongation of the QT interval. Am J Med Genet A. 2018 Jul;176(7):1657–61.

64. Bozarth X, Dines JN, Cong Q, Mirzaa GM, Foss K, Lawrence Merritt J 2nd, et al. Expanding clinical phenotype in CACNA1C related disorders: From neonatal onset severe epileptic encephalopathy to late-onset epilepsy. Am J Med Genet A. 2018 Dec;176(12):2733–9.

65. Splawski I, Timothy KW, Decher N, Kumar P, Sachse FB, Beggs AH, et al. Severe arrhythmia disorder caused by cardiac L-type calcium channel mutations. Proc Natl Acad Sci U S A. 2005 Jun;102(23):8088–9.

66. White JA, McKinney BC, John MC, Powers PA, Kamp TJ, Murphy GG. Conditional forebrain deletion of the L-type calcium channel Ca V 1.2 disrupts remote spatial memories in mice. Learn Mem. 2008 Jan;15(1):1–5.

67. Temme SJ, Bell RZ, Fisher GL, Murphy GG. Deletion of the Mouse Homolog of CACNA1C Disrupts Discrete Forms of Hippocampal-Dependent Memory and Neurogenesis within the Dentate Gyrus. eNeuro. 2016;3(6).

68. Miranda AS, Cardozo PL, Silva FR, de Souza JM, Olmo IG, Cruz JS, et al. Alterations of Calcium Channels in a Mouse Model of Huntington’s Disease and Neuroprotection by Blockage of Ca(V)1 Channels. ASN Neuro. 2019;11:1759091419856811.

69. Abd Allah ESH, Gomaa AMS, Sayed MM. The effect of omega-3 on cognition in hypothyroid adult male rats. Acta Physiol Hung. 2014 Sep;101(3):362–76.

70. Lee AS, De Jesús-Cortés H, Kabir ZD, Knobbe W, Orr M, Burgdorf C, et al. The Neuropsychiatric Disease-Associated Gene cacna1c Mediates Survival of Young Hippocampal Neurons. eNeuro. 2016;3(2).

71. Braun MD, Kisko TM, Vecchia DD, Andreatini R, Schwarting RKW, Wöhr M. Sex-specific effects of Cacna1c haploinsufficiency on object recognition, spatial memory, and reversal learning capabilities in rats. Neurobiol Learn Mem. 2018 Nov;155:543–55.

72. Kabitzke PA, Brunner D, He D, Fazio PA, Cox K, Sutphen J, et al. Comprehensive analysis of two Shank3 and the Cacna1c mouse models of autism spectrum disorder. Genes Brain Behav. 2018 Jan;17(1):4–22.

73. Koppe G, Mallien AS, Berger S, Bartsch D, Gass P, Vollmayr B, et al. CACNA1C gene regulates behavioral strategies in operant rule learning. PLoS Biol. 2017 Jun;15(6):e2000936.

74. Dedic N, Pöhlmann ML, Richter JS, Mehta D, Czamara D, Metzger MW, et al. Cross-disorder risk gene CACNA1C differentially modulates susceptibility to psychiatric disorders during development and adulthood. Mol Psychiatry. 2018 Mar;23(3):533–43.

75. Pinggera A, Mackenroth L, Rump A, Schallner J, Beleggia F, Wollnik B, et al. New gain-of-function mutation shows CACNA1D as recurrently mutated gene in autism spectrum disorders and epilepsy. Hum Mol Genet. 2017 Aug;26(15):2923–32.

76. Flanagan SE, Vairo F, Johnson MB, Caswell R, Laver TW, Lango Allen H, et al. A CACNA1D mutation in a patient with persistent hyperinsulinaemic hypoglycaemia, heart defects, and severe hypotonia. Vol. 18, Pediatric diabetes. Denmark; 2017. p. 320–3.

77. Hofer NT, Tuluc P, Ortner NJ, Nikonishyna Y V, Fernándes-Quintero ML, Liedl KR, et al. Biophysical classification of a CACNA1D de novo mutation as a high-risk mutation for a severe neurodevelopmental disorder. Mol Autism. 2020;11(1):4.

78. Thibault O, Landfield PW. Increase in single L-type calcium channels in hippocampal neurons during aging. Science. 1996 May;272(5264):1017–20.

79. Krueger JN, Moore SJ, Parent R, McKinney BC, Lee A, Murphy GG. A novel mouse model of the aged brain: Over-expression of the L-type voltage-gated calcium channel CaV1.3. Behav Brain Res. 2017 Mar;322(Pt B):241–9.

80. Dragicevic E, Poetschke C, Duda J, Schlaudraff F, Lammel S, Schiemann J, et al. Cav1.3 channels control D2-autoreceptor responses via NCS-1 in substantia nigra dopamine neurons. Brain. 2014 Aug;137(Pt 8):2287–302.

81. Ortner NJ, Bock G, Dougalis A, Kharitonova M, Duda J, Hess S, et al. Lower Affinity of Isradipine for L-Type Ca(2+) Channels during Substantia Nigra Dopamine Neuron-Like Activity: Implications for Neuroprotection in Parkinson’s Disease. J Neurosci. 2017 Jul;37(28):6761–77.

82. Stanika RI, Villanueva I, Kazanina G, Andrews SB, Pivovarova NB. Comparative impact of voltage-gated calcium channels and NMDA receptors on mitochondria-mediated neuronal injury. J Neurosci. 2012 May;32(19):6642–50.

83. Kamp MA, Krieger A, Henry M, Hescheler J, Weiergraber M, Schneider T. Presynaptic “Ca2.3-containing” E-type Ca channels share dual roles during neurotransmitter release. Eur J Neurosci. 2005 Mar;21(6):1617–25.

84. Newcomb R, Szoke B, Palma A, Wang G, Chen X h, Hopkins W, et al. Selective peptide antagonist of the class E calcium channel from the venom of the tarantula Hysterocrates gigas. Biochemistry. 1998 Nov;37(44):15353–62.

85. Gutzmann JJ, Lin L, Hoffman DA. Functional Coupling of Cav2.3 and BK Potassium Channels Regulates Action Potential Repolarization and Short-Term Plasticity in the Mouse Hippocampus. Front Cell Neurosci [Internet]. 2019 Feb 21;13:27. Available from: https://pubmed.ncbi.nlm.nih.gov/30846929

86. Gasparini S, Kasyanov AM, Pietrobon D, Voronin LL, Cherubini E. Presynaptic R-type calcium channels contribute to fast excitatory synaptic transmission in the rat hippocampus. J Neurosci. 2001 Nov;21(22):8715–21.

87. Helbig KL, Lauerer RJ, Bahr JC, Souza IA, Myers CT, Uysal B, et al. De Novo Pathogenic Variants in CACNA1E Cause Developmental and Epileptic Encephalopathy with Contractures, Macrocephaly, and Dyskinesias. Am J Hum Genet. 2018 Nov;103(5):666–78.

88. Gray EE, Murphy JG, Liu Y, Trang I, Tabor GT, Lin L, et al. Disruption of GpI mGluR-Dependent Cav2.3 Translation in a Mouse Model of Fragile X Syndrome. J Neurosci. 2019 Sep;39(38):7453–64.

89. Jansen R, Timmerman J, Loos M, Spijker S, van Ooyen A, Brussaard AB, et al. Novel candidate genes associated with hippocampal oscillations. PLoS One. 2011;6(10):e26586.

90. Weiergraber M, Henry M, Ho MSP, Struck H, Hescheler J, Schneider T. Altered thalamocortical rhythmicity in Ca(v)2.3-deficient mice. Mol Cell Neurosci. 2008 Dec;39(4):605–18.

91. Park J-Y, Spruston N. Synergistic actions of metabotropic acetylcholine and glutamate receptors on the excitability of hippocampal CA1 pyramidal neurons. J Neurosci. 2012 May;32(18):6081–91.

92. Morgans CW. Localization of the alpha(1F) calcium channel subunit in the rat retina. Invest Ophthalmol Vis Sci. 2001 Sep;42(10):2414–8.

93. Morgans CW, Gaughwin P, Maleszka R. Expression of the alpha1F calcium channel subunit by photoreceptors in the rat retina. Mol Vis. 2001 Aug;7:202–9.

94. Bech-Hansen NT, Naylor MJ, Maybaum TA, Pearce WG, Koop B, Fishman GA, et al. Loss-of-function mutations in a calcium-channel alpha1-subunit gene in Xp11.23 cause incomplete X-linked congenital stationary night blindness. Nat Genet. 1998 Jul;19(3):264–7.

95. Strom TM, Nyakatura G, Apfelstedt-Sylla E, Hellebrand H, Lorenz B, Weber BH, et al. An L-type calcium-channel gene mutated in incomplete X-linked congenital stationary night blindness. Nat Genet. 1998 Jul;19(3):260–3.

96. Xiao H, Chen X, Steele ECJ. Abundant L-type calcium channel Ca(v)1.3 (alpha1D) subunit mRNA is detected in rod photoreceptors of the mouse retina via in situ hybridization. Mol Vis. 2007 May;13:764–71.

97. Doering CJ, Hamid J, Simms B, McRory JE, Zamponi GW. Cav1.4 encodes a calcium channel with low open probability and unitary conductance. Biophys J. 2005 Nov;89(5):3042–8.

98. Doering CJ, Peloquin JB, McRory JE. The Ca(v)1.4 calcium channel: more than meets the eye. Channels (Austin). 2007;1(1):3–10.

99. Hope CI, Sharp DM, Hemara-Wahanui A, Sissingh JI, Lundon P, Mitchell EA, et al. Clinical manifestations of a unique X-linked retinal disorder in a large New Zealand family with a novel mutation in CACNA1F, the gene responsible for CSNB2. Clin Experiment Ophthalmol. 2005 Apr;33(2):129–36.

100. Laird JG, Gardner SH, Kopel AJ, Kerov V, Lee A, Baker SA. Rescue of Rod Synapses by Induction of Cav Alpha 1F in the Mature Cav1.4 Knock-Out Mouse Retina. Invest Ophthalmol Vis Sci. 2019 Jul;60(8):3150–61.

101. Knoflach D, Kerov V, Sartori SB, Obermair GJ, Schmuckermair C, Liu X, et al. Cav1.4 IT mouse as model for vision impairment in human congenital stationary night blindness type 2. Channels (Austin). 2013;7(6):503–13.

102. Ly R, Bouvier G, Schonewille M, Arabo A, Rondi-Reig L, Léna C, et al. T-type channel blockade impairs long-term potentiation at the parallel fiber-Purkinje cell synapse and cerebellar learning. Proc Natl Acad Sci U S A. 2013 Dec;110(50):20302–7.

103. Isope P, Hildebrand ME, Snutch TP. Contributions of T-type voltage-gated calcium channels to postsynaptic calcium signaling within Purkinje neurons. Cerebellum. 2012 Sep;11(3):651–65.

104. Wolfart J, Roeper J. Selective coupling of T-type calcium channels to SK potassium channels prevents intrinsic bursting in dopaminergic midbrain neurons. J Neurosci. 2002 May;22(9):3404–13.

105. Chemin J, Siquier-Pernet K, Nicouleau M, Barcia G, Ahmad A, Medina-Cano D, et al. De novo mutation screening in childhood-onset cerebellar atrophy identifies gain-of-function mutations in the CACNA1G calcium channel gene. Brain. 2018 Jul;141(7):1998–2013.

106. Barresi S, Dentici ML, Manzoni F, Bellacchio E, Agolini E, Pizzi S, et al. Infantile-Onset Syndromic Cerebellar Ataxia and CACNA1G Mutations. Pediatr Neurol. 2020 Mar;104:40–5.

107. Najmabadi H, Hu H, Garshasbi M, Zemojtel T, Abedini SS, Chen W, et al. Deep sequencing reveals 50 novel genes for recessive cognitive disorders. Nature. 2011 Sep;478(7367):57–63.

108. Yabuki Y, Matsuo K, Izumi H, Haga H, Yoshida T, Wakamori M, et al. Pharmacological properties of SAK3, a novel T-type voltage-gated Ca(2+) channel enhancer. Neuropharmacology. 2017 May;117:1–13.

109. Joksimovic SM, Eggan P, Izumi Y, Joksimovic SL, Tesic V, Dietz RM, et al. The role of T-type calcium channels in the subiculum: to burst or not to burst? J Physiol. 2017 Oct;595(19):6327–48.

110. Zamponi GW, Striessnig J, Koschak A, Dolphin AC. The Physiology, Pathology, and Pharmacology of Voltage-Gated Calcium Channels and Their Future Therapeutic Potential. Pharmacol Rev. 2015 Oct;67(4):821–70.

111. Han JY, Jang W, Park J, Kim M, Kim Y, Lee IG. Diagnostic approach with genetic tests for global developmental delay and/or intellectual disability: Single tertiary center experience. Ann Hum Genet. 2019 May;83(3):115–23.

112. Dumenieu M, Senkov O, Mironov A, Bourinet E, Kreutz MR, Dityatev A, et al. The Low-Threshold Calcium Channel Cav3.2 Mediates Burst Firing of Mature Dentate Granule Cells. Cereb Cortex. 2018 Jul;28(7):2594–609.

113. Chen C-C, Shen J-W, Chung N-C, Min M-Y, Cheng S-J, Liu IY. Retrieval of context-associated memory is dependent on the Ca(v)3.2 T-type calcium channel. PLoS One. 2012;7(1):e29384.

114. Uebachs M, Schaub C, Perez-Reyes E, Beck H. T-type Ca2+ channels encode prior neuronal activity as modulated recovery rates. J Physiol. 2006 Mar;571(Pt 3):519–36.

115. Xie Y, Huang D, Wei L, Luo X-J. Further evidence for the genetic association between CACNA1I and schizophrenia. Hereditas. 2018;155:16.

116. Perez-Reyes E. Molecular physiology of low-voltage-activated t-type calcium channels. Physiol Rev. 2003 Jan;83(1):117–61.

117. Cain SM, Snutch TP. Contributions of T-type calcium channel isoforms to neuronal firing. Channels (Austin). 2010;4(6):475–82.

118. Andrade A, Hope J, Allen A, Yorgan V, Lipscombe D, Pan JQ. A rare schizophrenia risk variant of CACNA1I disrupts Ca(V)3.3 channel activity. Sci Rep. 2016 Oct;6:34233.

119. El Ghaleb Y, Schneeberger PE, Fernández-Quintero ML, Geisler SM, Pelizzari S, Polstra AM, et al. CACNA1I gain-of-function mutations differentially affect channel gating and cause neurodevelopmental disorders. Brain. 2021 Mar;

120. Astori S, Lüthi A. Synaptic plasticity at intrathalamic connections via CaV3.3 T-type Ca2+ channels and GluN2B-containing NMDA receptors. J Neurosci. 2013 Jan;33(2):624–30.

121. Punetha J, Karaca E, Gezdirici A, Lamont RE, Pehlivan D, Marafi D, et al. Biallelic CACNA2D2 variants in epileptic encephalopathy and cerebellar atrophy. Ann Clin Transl Neurol. 2019 Aug;6(8):1395–406.

122. Cole RL, Lechner SM, Williams ME, Prodanovich P, Bleicher L, Varney MA, et al. Differential distribution of voltage-gated calcium channel alpha-2 delta (alpha2delta) subunit mRNA-containing cells in the rat central nervous system and the dorsal root ganglia. J Comp Neurol. 2005 Oct;491(3):246–69.

123. Dolphin AC. Calcium channel auxiliary alpha2delta and beta subunits: trafficking and one step beyond. Nat Rev Neurosci. 2012 Jul;13(8):542–55.

124. Geisler S, Schopf CL, Obermair GJ. Emerging evidence for specific neuronal functions of auxiliary calcium channel alpha(2)delta subunits. Gen Physiol Biophys. 2015 Apr;34(2):105–18.

125. Zhou J-J, Li D-P, Chen S-R, Luo Y, Pan H-L. The α2δ-1-NMDA receptor coupling is essential for corticostriatal long-term potentiation and is involved in learning and memory. J Biol Chem. 2018 Dec;293(50):19354–64.

126. Vergult S, Dheedene A, Meurs A, Faes F, Isidor B, Janssens S, et al. Genomic aberrations of the CACNA2D1 gene in three patients with epilepsy and intellectual disability. Eur J Hum Genet. 2015 May;23(5):628–32.

127. Siddique A, Willoughby J, McNeill A. A 7q21.11 microdeletion presenting with apparent intellectual disability without epilepsy. Vol. 173, American journal of medical genetics. Part A. United States; 2017. p. 1128–30.

128. Lau LA, Noubary F, Wang D, Dulla CG. alpha2delta-1 Signaling Drives Cell Death, Synaptogenesis, Circuit Reorganization, and Gabapentin-Mediated Neuroprotection in a Model of Insult-Induced Cortical Malformation. eNeuro. 2017;4(5).

129. Faria LC, Gu F, Parada I, Barres B, Luo ZD, Prince DA. Epileptiform activity and behavioral arrests in mice overexpressing the calcium channel subunit alpha2delta-1. Neurobiol Dis. 2017 Jun;102:70–80.

130. Gong HC, Hang J, Kohler W, Li L, Su TZ. Tissue-specific expression and gabapentin-binding properties of calcium channel alpha2delta subunit subtypes. J Membr Biol. 2001 Nov;184(1):35–43.

131. Hoppa MB, Lana B, Margas W, Dolphin AC, Ryan TA. alpha2delta expression sets presynaptic calcium channel abundance and release probability. Nature. 2012 May;486(7401):122–5.

132. Ivanov S V, Ward JM, Tessarollo L, McAreavey D, Sachdev V, Fananapazir L, et al. Cerebellar ataxia, seizures, premature death, and cardiac abnormalities in mice with targeted disruption of the Cacna2d2 gene. Am J Pathol. 2004 Sep;165(3):1007–18.

133. Edvardson S, Oz S, Abulhijaa FA, Taher FB, Shaag A, Zenvirt S, et al. Early infantile epileptic encephalopathy associated with a high voltage gated calcium channelopathy. J Med Genet. 2013 Feb;50(2):118–23.

134. Butler KM, Holt PJ, Milla SS, da Silva C, Alexander JJ, Escayg A. Epileptic Encephalopathy and Cerebellar Atrophy Resulting from Compound Heterozygous CACNA2D2 Variants. Vol. 2018, Case reports in genetics. United States; 2018. p. 6308283.

135. Pippucci T, Parmeggiani A, Palombo F, Maresca A, Angius A, Crisponi L, et al. A novel null homozygous mutation confirms CACNA2D2 as a gene mutated in epileptic encephalopathy. PLoS One. 2013;8(12):e82154.

136. Barclay J, Balaguero N, Mione M, Ackerman SL, Letts VA, Brodbeck J, et al. Ducky mouse phenotype of epilepsy and ataxia is associated with mutations in the Cacna2d2 gene and decreased calcium channel current in cerebellar Purkinje cells. J Neurosci. 2001 Aug;21(16):6095–104.

137. Meier H. The neuropathology of ducky, a neurological mutation of the mouse. A pathological and preliminary histochemical study. Acta Neuropathol. 1968 Jul;11(1):15–28.
